# The tumour microenvironment harbours ontogenically distinct dendritic cell populations with opposing effects on tumour immunity

**DOI:** 10.1038/ncomms13720

**Published:** 2016-12-23

**Authors:** Damya Laoui, Jiri Keirsse, Yannick Morias, Eva Van Overmeire, Xenia Geeraerts, Yvon Elkrim, Mate Kiss, Evangelia Bolli, Qods Lahmar, Dorine Sichien, Jens Serneels, Charlotte L. Scott, Louis Boon, Patrick De Baetselier, Massimiliano Mazzone, Martin Guilliams, Jo A. Van Ginderachter

**Affiliations:** 1 Laboratory of Myeloid Cell Immunology, VIB Inflammation Research Center, 9000 Ghent, Belgium; 2 Laboratory of Cellular and Molecular Immunology, Vrije Universiteit Brussel, 1050 Brussels, Belgium; 3 Unit of Immunoregulation and Mucosal Immunology, VIB Inflammation Research Center, 9000 Ghent, Belgium; 4 Department of Biomedical Molecular Biology, Ghent University, 9000 Ghent, Belgium; 5 Laboratory of Molecular Oncology and Angiogenesis, Vesalius Research Center, VIB, 3000 Leuven, Belgium; 6 Laboratory of Molecular Oncology and Angiogenesis, Vesalius Research Center, K.U. Leuven, 3000 Leuven, Belgium; 7 Epirus Biopharmaceuticals NL, 3584 CM Utrecht, The Netherlands

## Abstract

Various steady state and inflamed tissues have been shown to contain a heterogeneous DC population consisting of developmentally distinct subsets, including cDC1s, cDC2s and monocyte-derived DCs, displaying differential functional specializations. The identification of functionally distinct tumour-associated DC (TADC) subpopulations could prove essential for the understanding of basic TADC biology and for envisaging targeted immunotherapies. We demonstrate that multiple mouse tumours as well as human tumours harbour ontogenically discrete TADC subsets. Monocyte-derived TADCs are prominent in tumour antigen uptake, but lack strong T-cell stimulatory capacity due to NO-mediated immunosuppression. Pre-cDC-derived TADCs have lymph node migratory potential, whereby cDC1s efficiently activate CD8^+^ T cells and cDC2s induce Th17 cells. Mice vaccinated with cDC2s displayed a reduced tumour growth accompanied by a reprogramming of pro-tumoural TAMs and a reduction of MDSCs, while cDC1 vaccination strongly induces anti-tumour CTLs. Our data might prove important for therapeutic interventions targeted at specific TADC subsets or their precursors.

## Abstract (web summary)

Dendritic cells are antigen-presenting cells consisting of distinct subsets originating from different lineages. Here, the authors identify the subsets of dendritic cells populating the tumour tissue in both mice and humans and find they have opposing functions in regulating the anti-tumour immune response.

Dendritic cells (DCs) are specialized antigen-presenting cells, present in all tissues, that play a major role in orchestrating immune responses^1^. The presence of mature DCs in tumours has been correlated with a positive prognosis in several tumour types^2^,^3^. However, multiple clinical studies have indicated a defective functionality and scarcity of mature DCs in tumours^4^,^5^,^6^. In addition, DCs seem to switch from an immunostimulatory activation state driving anti-tumour immunity in early stage tumours to an immunosuppressive activation state at later stages^7^. The secretion of immunosuppressive factors by cancer cells has been proposed to be implicated in the control of DC differentiation, maturation and function^4^,^8^. In addition, tumour-associated DCs (TADCs) may favour tumour progression by mediating genomic damage, supporting neovascularization and stimulating cancerous cell growth and spreading^4^,^9^,^10^, features that may be attributed to the existence of distinct TADC populations^10^.

Although not much is known about DC heterogeneity in tumours, DCs isolated from various steady-state and inflamed tissues have been shown to represent a heterogeneous population consisting of developmentally distinct DC subsets^11^,^12^,^13^, including cDC1s (CD8α^+^-like or CD103^+^ conventional DCs), cDC2s (CD11b^+^-like cDCs), plasmacytoid DCs (pDCs) and so-called monocyte-derived DCs (Mo-DCs)^12^,^14^,^15^. Notably, distinct DC classification systems and nomenclatures have been used. Throughout this manuscript, we employ the ontogeny-based classification/nomenclature as proposed by Guilliams *et al*^15^. cDCs arise from bone marrow-derived pre-cDC precursors in an Flt3L-dependent fashion^16^, are maintained under homeostatic conditions by (granulocyte–macrophage colony-stimulating factor receptor) GM-CSFR signalling^17^ and differentiate into cDC1s and cDC2s under the control of BATF3, ID2 and IRF8 or RELB, ZEB2 and IRF4, respectively, while Mo-DCs differentiate from Ly6C^hi^ monocytes which exit the bone marrow in a CCR2-dependent manner and were reported not to require GM-CSFR signalling for their *in vivo* differentiation^17^,^18^,^19^. Importantly, transcriptomic analysis of mouse and human DC subsets revealed that human CD141 (BDCA3)^+^ DCs are related to mouse cDC1s, whereas human CD1c (BDCA1)^+^ DCs are more related to mouse cDC2s (ref. 20). Human CD141^+^ DCs express Batf3 and IRF8 and lack expression of IRF4, akin to mouse cDC1s. Moreover, the differentiation of human haematopoietic progenitors into CD141^+^ DCs occurs only when Flt3L is added to the cultures, and inhibition of Batf3 in these cultures abolishes the differentiation of CD141^+^ DCs but not of CD1c^+^ DCs, suggesting that CD141^+^ DCs are indeed developmentally related to mouse cDC1s.

Importantly, DCs of distinct cellular origin have been shown to display a differential functional specialization. While cDC1s are specialized in the induction of cytotoxic T-cell (CTL) responses, cDC2s have been shown to excel at the induction of Th17 or Th2 responses^13^,^21^,^22^,^23^. Although the migratory potential of Mo-DCs is debated, they have been proposed to reactivate effector T cells in inflamed tissues^13^. Whether the various functions ascribed to TADCs are in fact performed by distinct DC subsets is unknown, but the recent elegant report of cDC1 presence in tumours^24^ emphasizes that the tumour tissue may, like any other tissue, be populated by DCs with distinct developmental origin and possibly a differential functional specialization. As a matter of fact, subpopulations of tumour-associated macrophages (TAMs) with distinct functions have been identified^25^,^26^. Here, we aimed to investigate the generation and function of ontogenically distinct DC populations and to assess their potential for inducing anti-tumour responses. Our data unveil the complexity of the TADC compartment, which is for the first time demonstrated to consist of both pre-cDC and monocyte-derived DC subsets in tumours, and might prove important for therapeutic interventions targeted at specific TADC subsets or their precursors.

## Results

### Distinct TADC subsets derive from different precursors

**Figure 1:**
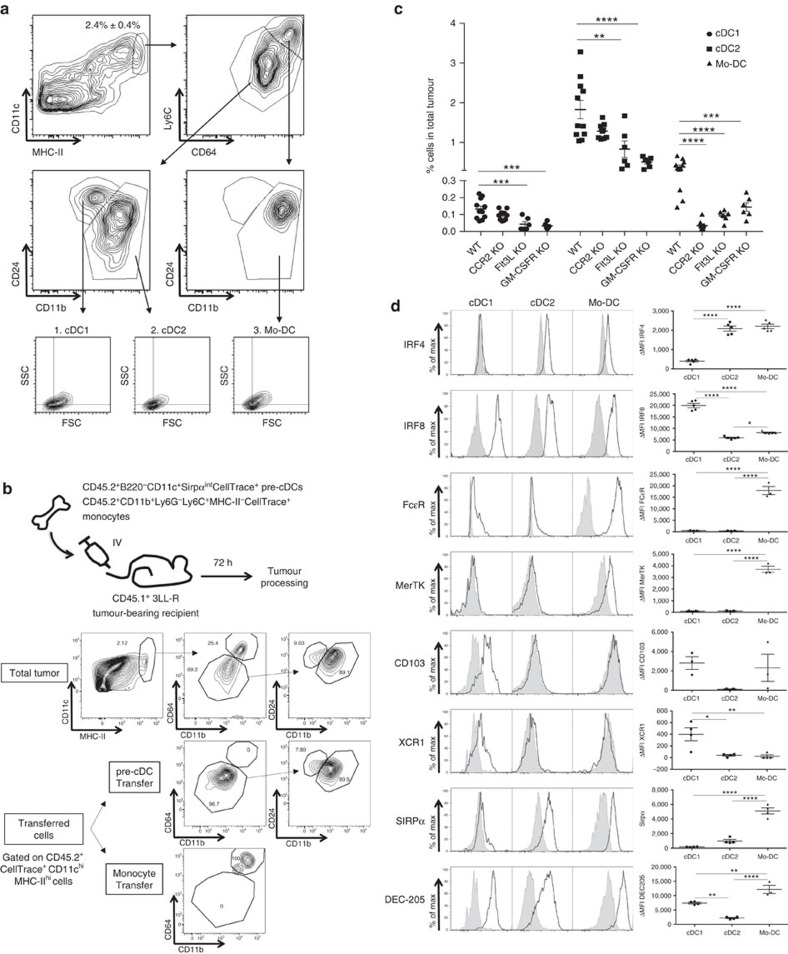
Origin of different TADC subpopulations. (**a**) TADCs of 12-day-old 3LL-R tumours were subdivided into (1). CD64^neg^ CD24^pos^ CD11b^lo^ cDC1s, (2). CD64^neg^ CD24^neg^ CD11b^pos^ Ly6C^lo^ cDC2s and (3). CD64^pos^ CD24^int^ CD11b^pos^ Ly6C^hi^ Mo-DCs. For each subset, forward scatter versus side scatter plots are shown. Results are representative of four independent experiments with *n*≥4. (**b**) Pre-cDCs (B220^−^CD11c^+^Sirpα^int^) and monocyte precursors (CD11b^+^Ly6G^−^Ly6C^+^MHC-II^−^) were sorted from CD45.2^+^ bone marrow and labelled with CellTrace. Either 4 × 10^5^ pre-cDCs or 1 × 10^6^ monocytes were adoptively transferred (IV) to CD45.1 7-day-old 3LL-R tumour-bearing recipient mice. Three days later, tumours were processed and transferred cells were gated based on their CD45.1^−^CD45.2^+^CellTrace^+^ phenotype. Results are representative of two independent experiments with *n*=2–4. (**c**) 3LL-R tumours were grown for 12 days in WT, CCR2-KO, Flt3L-KO and GM-CSFR KO mice. The percentage of each TADC subpopulation within the total tumour single-cell suspension was determined. Results are representative of two independent experiments with *n*=6. Statistical analysis by two-way ANOVA. *P<0.05; **P<0.01; ***P<0.001; ****P<0.0001. (**d**) Single-cell suspensions of 12-day-old 3LL-R tumours were stained for the indicated markers and histogram overlays are shown for the TADC subsets. Black line=expression of the indicated marker; shaded histogram=isotype control. ΔMFI±s.e.m. are indicated and represent (MFI marker−MFI control). Results are representative of two independent experiments with *n*≥4. Statistical analysis by one-way ANOVA. *P<0.05; **P<0.01; ***P<0.001; ****P<0.0001.

To delineate the relative abundance of distinct tumour-associated DC (TADC) populations in solid tumours, we first employed the 3LL-R Lewis Lung Carcinoma model, which is known to be strongly infiltrated by myeloid cells^26^. These tumours contain a sizeable population of CD3^neg^ CD19^neg^ Ly6G^neg^ CD11c^hi^ MHC-II^hi^ TADCs (Fig. 1a). Earlier studies characterized distinct DC populations based on their differential expression of CD24, CD11b, Ly6C and CD64 (ref. 27). Using this approach, three discrete TADC subsets were clearly distinguishable (Fig. 1a): Ly6C^lo^ CD64^lo^ CD24^+^ CD11b^lo^ conventional TADCs (cDC1s, gate 1), Ly6C^lo^ CD64^lo^ CD24^int-lo^ CD11b^+^ conventional TADCs (cDC2s, gate 2) and Ly6C^hi^ CD64^hi^ CD24^int^ CD11b^+^ monocyte-derived TADCs (Mo-DCs, gate 3). This situation is similar to what has been reported in several non-cancerous tissues^12^.

We further assessed the origin of TADC subsets by adoptive transfer of pre-cDCs and monocytes in 3LL-R tumour-bearing mice. When adoptively transferring sorted CD45.2^+^ B220^−^ CD11c^+^ Sirpα^int^ CellTrace^+^ bone marrow pre-cDC precursors^28^ in 3LL-R tumour-bearing CD45.1^+^ recipient mice, only Ly6C^lo^ CD64^lo^ CD24^+^ CD11b^lo^ and Ly6C^lo^ CD64^lo^ CD24^int-lo^ CD11b^+^ cells could be retrieved from tumours after 72 h (Fig. 1b). Importantly, none of the transferred pre-cDC precursors differentiated into CD64^hi^ CD11b^+^ cells during this time span. In contrast, transferred CD45.2^+^ CD11b^+^ Ly6G^−^ Ly6C^+^ MHC-II^−^ CellTrace^+^ bone marrow monocytes were all retrieved as CD64^hi^ CD11b^+^ cells within the TADC gate. Note that relatively low amounts of CD45.2^+^ Mo-DCs were retrieved from tumours since most transferred monocytes gave rise to MHC-II^low^ and MHC-II^high^ TAMs, which make up the bulk of monocyte-derived cells in 3LL-R tumours (Supplementary Fig. 1). Altogether these data indicate that Ly6C^lo^ CD64^lo^ CD24^+^ CD11b^lo^, Ly6C^lo^ CD64^lo^ CD24^int-lo^ CD11b^+^ and CD64^hi^ CD11b^+^ cells represent cDC1, cDC2 and Mo-DCs, respectively.

Corroborating the adoptive transfer experiments, 3LL-R tumours grown in CCR2-deficient mice, in which the egression of monocytes from the bone marrow is strongly reduced^18^, showed almost complete absence of the Mo-DC subset while cDC1s and cDC2s were unaffected (Fig. 1c). In line with their cDC ontogeny, the amounts of tumour-associated cDC1s and cDC2s were strongly reduced in both Flt3L-KO and GM-CSFR-KO mice, showing their dependence on both Flt3L and GM-CSF (Fig. 1c). The residual presence of cDC2s in tumours in both KO strains suggests that their generation and/or survival may depend on the simultaneous functioning of (other) haematopoietic growth factors in the tumour microenvironment^29^. Importantly, also Mo-DCs were heavily affected by the absence of Flt3L and the inability to respond to GM-CSF. Whether this drop in Mo-DCs reflects a direct effect of Flt3L and/or GM-CSF on Mo-DC differentiation/maintenance, or whether the loss of cDC subsets in these KO tumours influences the development of Mo-DCs, is not clear at present.

Following the fate mapping experiments, we further characterized the TADC subsets for markers that were reported to associate with different DC populations. In this respect, the need for IRF8 and IRF4 in the development of cDC1s and cDC2s, respectively, has been established^30^. Intracellular staining for these transcription factors confirmed the higher expression of IRF4 in cDC2s and of IRF8 in cDC1s (Fig. 1d). Interestingly, Mo-DCs appeared to contain relatively high levels of IRF4 and low levels of IRF8. In agreement with their monocytic origin, the Mo-DCs expressed high levels of FcɛR (Fig. 1d), previously associated with monocyte-derived DCs^13^. Furthermore, the Mo-DC population was also the only subset expressing the macrophage-related marker MerTK^27^,^31^. Both CD103 and the cross-presentation related marker XCR1 seem to be uniquely expressed on the cDC1 subset, whereas SIRPα expression was limited to the Mo-DC and cDC2 subsets (Fig. 1d). Finally, DEC205 is expressed on all TADC subsets with Mo-DC>cDC1>cDC2 (Fig. 1d).

### Distinct TADC subsets are present in multiple tumour types

**Figure 2:**
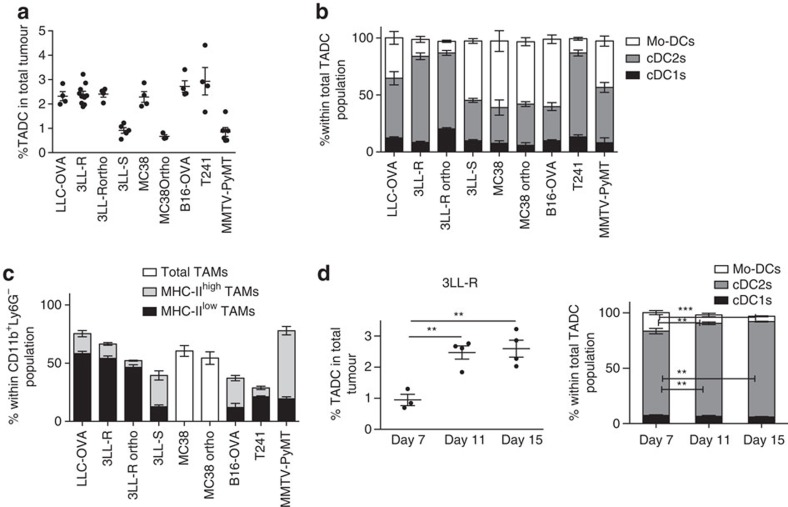
Several tumour types are infiltrated with distinct TADC subsets. (**a**) TADCs were gated as doublets^neg^ live (AQUA^neg^) Ly6G^neg^ CD3^neg^ CD19^neg^ CD11c^pos^ MHC-II^pos^ cells in single-cell suspensions of 20-day-old subcutaneous (s.c.) LLC-OVA lung carcinoma, 12-day-old s.c. 3LL-R lung carcinoma, 6-day-old 3LL-R orthotopically injected lung carcinoma, 35-day-old s.c. 3LL-S lung carcinoma, 17-day-old s.c. MC38 colon carcinoma, 28-day-old MC38 orthotopically injected colon carcinoma, 20-day-old B16-OVA s.c. melanoma, 20-day-old T241 s.c. fibrosarcoma and 16-week-old spontaneously grown MMTV-PyMT mammary carcinoma with tumours of similar volumes. (**b**) The percentage of each TADC subset within the total TADC population was determined for indicated tumours of similar volumes. (**c**) The percentage of each TAM subset within the total CD11b^+^ Ly6G^−^ population was determined for indicated tumours of similar volumes. (**a**–**c**) Graphs show mean±s.e.m. Results are representative of two independent experiments with *n*=3–10. (**d**) Amount of total TADCs (**d**, left panel) and TADC subsets (**d**, right panel) was assessed in single-cell suspensions of 7-, 11- and 15-day-old 3LL-R tumours. Graphs show mean±s.e.m. Results are representative of three independent experiments with *n*≥4. Statistical analysis by one-way ANOVA. *P<0.05; **P<0.01; ***P<0.001; ****P<0.0001.

We then assessed the presence of TADC subsets in several transplantable mouse tumour models of various histological origins and distinct genetic backgrounds and in the spontaneous MMTV-PyMT breast carcinoma model. Single-cell suspensions of subcutaneously growing LLC-OVA lung carcinoma tumours, their fast progressing 3LL-R and slowly progressing 3LL-S subclones^32^, MC38 colon carcinoma, B16 melanoma and T241 fibrosarcoma, as well as 3LL-R tumours orthotopically growing in the lung parenchyma and MC38 orthotopically growing in the rectum (all in C57Bl/6 background) contained small, but clearly identifiable CD11c^hi^ MHC-II^hi^ TADC fractions (Fig. 2a). TADCs were also retrieved from MMTV-PyMT mammary tumours in the FVB background (Fig. 2a).

When comparing the DC content of similarly sized tumours, the cDC1s were the rarest subset in all investigated models, whereas the cDC2s were always well represented (between 30.1% and 75.7% of all TADCs depending on the model) (Fig. 2b). The greatest variability was seen among the Mo-DCs, which were only poorly present in 3LL-R and T241 tumours, but form a sizeable population in LLC-OVA, MMTV-PyMT, 3LL-S, MC38 and B16 tumours, being even the dominant TADC population in the latter three models. Interestingly, the TADC content of subcutaneously versus orthotopically grown 3LL-R or MC38 tumours is very comparable, suggesting that the tumour microenvironment rather than the surrounding tissue dictates TADC heterogeneity. Notably, tumour models with a high Mo-DC content also harbour relatively more M1-like MHC-II^high^ TAMs as compared with their M2-like MHC-II^low^ counterparts (high MHC-II^high^/MHC-II^low^ TAM ratio) (Fig. 2c), suggesting that the microenvironment in these tumours favours the differentiation of infiltrating monocytes towards these MHC-II^high^ macrophages and DCs. Of note, MC38 tumours, which also contain a large Mo-DC population, contain a single TAM population displaying a mixed M1-like and M2-like phenotype (Fig. 2c). The correlation between Mo-DCs and MHC-II^high^ TAM presence is maintained within the same tumour model, whereby an underrepresentation of MHC-II^high^ TAMs and a gradual accumulation of MHC-II^low^ TAMs in the course of tumour growth is paralleled by a reduced percentage of Mo-DCs, as illustrated by the 3LL-R model (Fig. 2d and Supplementary Fig. 2A). However, the total percentage of 3LL-R-infiltrating TADCs increases during tumour growth (Fig. 2d). In contrast, 3LL-S tumours are invariably dominated by MHC-II^high^ TAMs (within the TAM compartment) and Mo-DCs (within the TADC compartment) throughout tumour growth and the percentage of TADCs did not change (Supplementary Fig. 2B,C).

### The distinct TADC subsets are also present in human tumours

**Figure 3:**
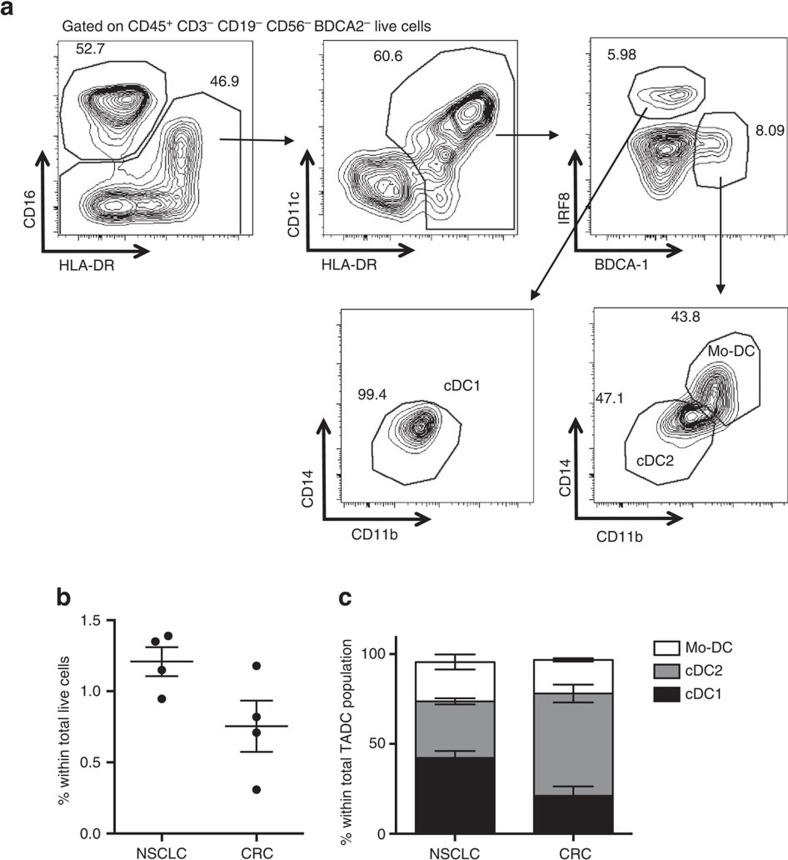
The presence of distinct TADC subsets can be recapitulated in human tumours. (**a**) Human NSCLC tumour biopsies were pre-gated on CD45^+^ CD3^−^ CD19^−^ CD56^−^ BDCA2^−^ live cells and CD16^−^ CD11c^high^ HLA-DR^+^ cells were subdivided into (i) BDCA1^−^ IRF8^+^ CD14^−^ CD11b^low^ cDC1s, (ii) BDCA1^+^ IRF8^−^ CD14^−^ CD11b^+^ cDC2s and (iii) BDCA1^+^ IRF8^−^ CD14^+^ CD11b^high^ Mo-DCs. (**b**,**c**) The total percentage of TADCs (sum of three subsets) (**b**) and the percentage of each TADC subset within the total TADC population (**c**) was determined for (NSCLC) and colorectal (CRC) tumours. For all experiments, graphs show mean±s.e.m. *n*=4 patients per tumour type.

To translate our original findings to the human situation, the presence of distinct TADC subpopulations was assessed in fresh tumour biopsies of lung and colorectal cancer patients. Small amounts of CD16^−^ CD11c^+^ HLA-DR^+^ TADCs were retrieved from human tumours (Fig. 3a,b and Supplementary Fig. 3A). Importantly, this TADC compartment encompassed three distinct subsets, highly reminiscent of the murine TADC subsets. Human TADCs contained a BDCA2^−^ BDCA1^−^ IRF8^+^ CD14^−^ CD11b^low^ cDC1 population, which also expressed the other commonly used cDC1 marker BDCA3 (Supplementary Fig. 3B), and a BDCA2^−^ IRF8^-^ BDCA1^+^ TADC fraction, which consisted of two subsets with differential CD14 and CD11b expression (Fig. 3a,c). As previously suggested^33^, these consist of a CD14^−^ CD11b^+^ cDC2 population and a CD14^+^ CD11b^high^ DC population, analogous to murine Mo-DCs. Of note, no BDCA2^+^ plasmacytoid DCs could be retrieved.

### Mo-DCs have the highest inherent antigen uptake capacity

Although the induction of potent antitumour immune responses has been attributed to DCs in some reports^2^,^34^, TADCs have also been described as tolerogenic or immunosuppressive cells with impaired antigen presenting, T-cell stimulating and migratory capacities, enabling tumour immune escape^4^,^10^,^34^,^35^. Hence, it could be postulated that distinct TADC subsets exert different functions. In first instance, we investigated the antigen uptake, processing and presenting capacities by the three distinct TADC populations.

Their inherent phagocytic capacity was tested *in vitro* by adding fluorescent latex beads to 3LL-R tumour single-cell suspensions at 37 °C (active phagocytosis) or at 4 °C (Supplementary Fig. 4A). All TADC subsets were able to ingest latex beads at 37 °C. However, when compared with the total TADC population, the proportion of cells within the latex^+^ TADC population only increased for the Mo-DC subset, signifying that this population was more phagocytic than both cDC types (Supplementary Fig. 4B). Interestingly, when performing an *in vivo* phagocytosis assay by injecting fluorescent latex beads intravenously into tumour-bearing mice 2 h before removing the tumour, the Mo-DCs were again overrepresented within the latex^+^ population, corroborating their superior phagocytic capacity *in vivo* (Supplementary Fig. 4C).

**Figure 4:**
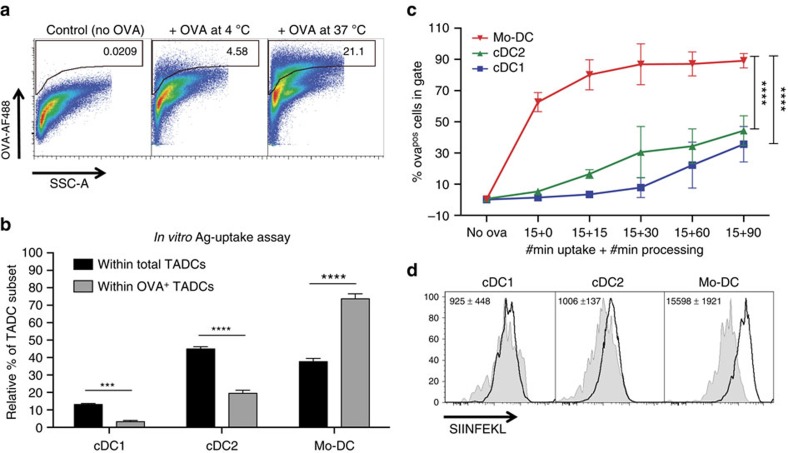
Antigen uptake and presentation differ in the distinct TADC subsets. (**a**,**b**) *In vitro* OVA-uptake assay. (**a**) Single-cell suspensions of 12-day-old 3LL-R tumours were cultured *in vitro*, in the absence (control) or presence of OVA-AF488 for 15 min at 4 or 37 °C. (**b**) The percentage of the distinct TADC subsets within the total TADC gate or within the OVA^+^ TADC gate are given. Results are representative of two independent experiments with *n*≥4. Statistical analysis by one-way ANOVA. ***P<0.001; ****P<0.0001. (**c**) DQ-OVA processing. 12-day-old 3LL-R tumour subsets were allowed to take up and process DQ-OVA for 15′ at 0 or 37 °C. Free DQ-OVA was subsequently removed from the culture medium and cells were given an additional 15, 30, 60 or 90 min to process internalized DQ-OVA. DQ-OVA processing results in the formation of fluorescent peptides and % of DQ-OVA^+^ TADCs are shown for each time point. *n*=3 pools of 4 tumours. Statistical analysis by two-way ANOVA. ****P<0.0001. (**d**) Cross-presentation by the different TADC subsets in LLC-OVA was assessed by staining for the OVA-derived peptide SIINFEKL in association with MHC-I. Black line=SIINFEKL expression of TADCs in LLC-OVA tumours; shaded histograms=SIINFEKL expression of TADCs in LLC tumours (control). ΔMFI are indicated and represent (MFI SIINFEKL in LLC-OVA−MFI SIINFEKL in LLC). Results are representative of two independent experiments with *n*=4.

The uptake of relatively large beads by phagocytosis is mechanistically distinct from the uptake of protein antigens, such as ovalbumin (OVA). Adding fluorescently labelled OVA to 3LL-R tumour single-cell suspensions for 15 min at 4 and 37 °C (active Ag uptake) revealed that all TADC subsets were able to ingest OVA at 37 °C (Fig. 4a), but that Mo-DCs again showed a higher Ag uptake capacity compared with both cDC types (Fig. 4b). To further test OVA processing by the different TADC subsets, 3LL-R tumour single-cell suspensions were incubated with DQ-OVA for 15 min, allowing antigen uptake. Following thorough washing, intracellular processing was assessed at different time intervals using the fluorescence of cleaved DQ-OVA as a readout. The vast majority of Mo-DCs rapidly incorporated and processed DQ-OVA (62.7±3.5% of fluorescent cells after 15 min), while hardly any processing had occurred in cDC1s and cDC2s at this early time point (Fig. 4c). Gradually more cDCs processed DQ-OVA, with a slightly higher efficiency for cDC2s compared with cDC1s (reaching 30.5±11.6% versus 7.8±4.6% fluorescent cells after 45 min, respectively). Together, these data illustrate the competitive advantage of Mo-DCs for antigen uptake, leading to subsequent antigen processing.

Finally, we assessed whether TADCs were capable of processing OVA and cross-presenting its immunodominant CTL epitope (SIINFEKL) *in vivo*, by staining freshly isolated TADC subsets from LLC-OVA tumours with an mAb specific for H-2K^b^/SIINFEKL complexes. Mo-DCs showed the highest expression of these complexes, indicative of a superior antigen uptake and/or processing in the tumour microenvironment (Fig. 4d).

### cDC2s induce a Th17 CD4^+^ T-cell phenotype

We then evaluated the capacity of TADC subsets to activate naive T cells. In this regard, the expression of activating and inhibitory T-cell costimulatory molecules, such as CD80, CD86, PDL1 and PDL2 was very high on all TADC populations (Supplementary Fig. 5A).

First, the intrinsic antigen-presenting capacity of TADC subsets, irrespective of their antigen uptake and processing capacity, was assessed in a classic mixed leukocyte reaction (MLR). All C57Bl/6 TADC subsets could activate naive Balb/c CD4^+^ and CD8^+^ T-cell proliferation, at least to the same extent as the control splenic CD11c^hi^MHC-II^hi^B220^−^Ly6C^−^ cDC population (Supplementary Fig. 5B,C). Interestingly, the cDC2s showed the highest intrinsic antigen-presenting capacity towards both CD4^+^ and CD8^+^ T cells.

**Figure 5:**
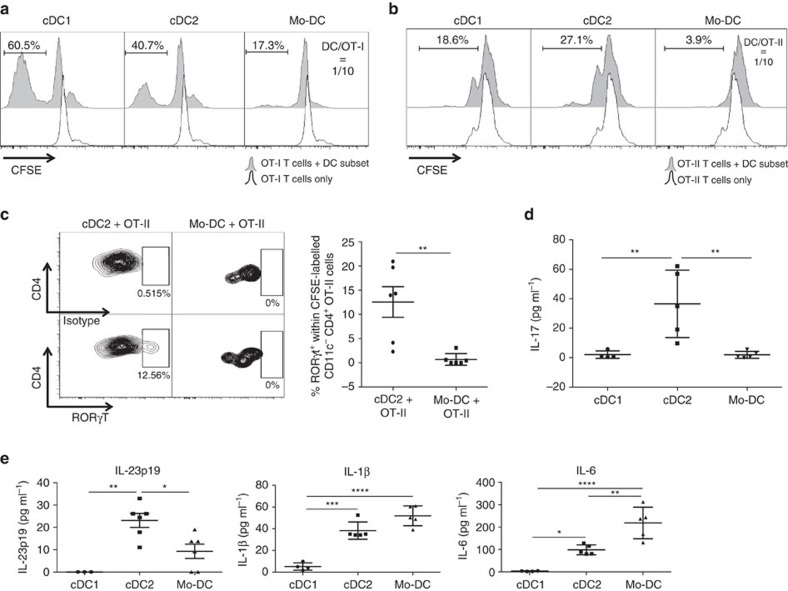
TADC subsets show distinct T-cell proliferative capacities. (**a**,**b**) Sorted TADC subsets were co-cultured with OT-I (**a**) or OT-II (**b**) T cells for 3 days at a DC/T-cell ratio of 1/10. The histograms represent CFSE dilution, indicative for T-cell proliferation. Black line=non-stimulated T cells without TADCs; shaded histogram=T cells in the presence of TADCs. Results are representative of three independent experiments with *n*=pool of 10–12 tumours. (**c**) Intracellular staining on OT-II T cells co-cultured with cDC2s or Mo-DCs for 3 days at a DC/OT-II ratio of 1/5 was performed for the Th-inducing transcription factor RORγt. Isotype control and transcription factor staining are depicted. *n*=6. Statistical analysis by one-way ANOVA. **P<0.01. (**d**) Supernatants of co-cultures of TADC subsets and OT-II T cells (DC/OT-II=1/10) were tested for the presence of IL-17 by luminex. *n*≥4. Statistical analysis by one-way ANOVA. **P<0.01. (**e**) Supernatants of TADC subsets cultured for 48 h were tested for the presence of IL-23p19, IL-6 and IL-1β by luminex. *n*≥4. Statistical analysis by one-way ANOVA. *P<0.05; **P<0.01; ***P<0.001; ****P<0.0001.

To incorporate the effect of a differential *in vivo* antigen uptake and processing capacity in our assay, TADCs were sorted from LLC-OVA tumours and immediately co-cultured with carboxyfluorescein succinimidyl ester (CFSE)-labelled TCR transgenic CD8^+^ OT-I T cells or CD4^+^ OT-II T cells without additional *ex vivo* Ag loading or stimulation. At a DC/OT-I ratio of 1/10, only the two cDC subsets could effectively induce CD8^+^ T-cell proliferation, hence demonstrating their *in vivo* immunostimulatory phenotype, whereby the cDC1s were considerably more potent (Fig. 5a). Also in the case of CD4^+^ T cells, only the cDC subsets were able to induce T-cell proliferation at a DC/OT-II ratio of 1/10, with now cDC2s being most efficient (Fig. 5b). Notably, within the timeframe of the experiment (3 days of stimulation), hardly any interferon-γ (IFN-γ) and interleukin-4 (IL-4) (Supplementary Fig. 5D) and no IL-13 (data not shown) could be detected in the supernatant of OT-II/TADC co-cultures, illustrating the lack of Th1 and Th2 induction by the TADC subsets.

Interestingly however, cDC2s induced the differentiation of a Th17 population, as demonstrated by the upregulation of RORγt—but not T-bet, GATA3 or FoxP3—in a population of OT-II cells, and the secretion of IL-17 in the supernatant (Fig. 5c,d and Supplementary Fig. 5E). Neither RORγt^+^ T cells nor IL-17 production were found in any other condition. The induction of Th17 may result from the inherently high production of Th17-inducing cytokines, such as IL-23, IL-1β and IL-6, by cDC2s (Fig. 5e). It should be noted that Mo-DCs secrete even higher levels of IL-1β and IL-6 but have a significantly lower IL-23 production, possibly explaining their lack of Th17-inducing capacity.

We next compared the T-cell activating capacity of tumour-associated cDC1s and cDC2s with cDC1 and cDC2 subsets from naive spleens (gating strategy in Supplementary Fig. 6A), which also expressed high levels of the costimulatory molecules CD80, CD86, PDL1 and PDL2 (Supplementary Fig. 6B). TADCs sorted from LLC tumours or naive splenic cDCs were co-cultured with CellTrace-labelled TCR transgenic CD8^+^ OT-I T cells or CD4^+^ OT-II T cells in the presence of OVA. At a DC/OT-I ratio of 1/10, tumour-derived cDC subsets induced CD8^+^ OT-I T-cell proliferation to the same extent as their splenic cDC counterparts (Supplementary Fig. 6C). Interestingly, in the case of CD4^+^ OT-II T-cells, the highest proliferation was measured when T cells were co-cultured with tumour-derived cDC2s (Supplementary Fig. 6D). Moreover, tumour-derived cDC2s produced significantly higher levels of the Th17-inducing cytokine IL-23 than splenic cDC2s in these co-cultures (Supplementary Fig. 6E). Consequently, high levels of IL-17 could only be detected in the co-cultures containing tumour-derived cDC2s, further illustrating the unique Th17 inducing capacity of these cells (Supplementary Fig. 6E).

### Mo-DCs display an immune suppressive TIP-DC phenotype

**Figure 6:**
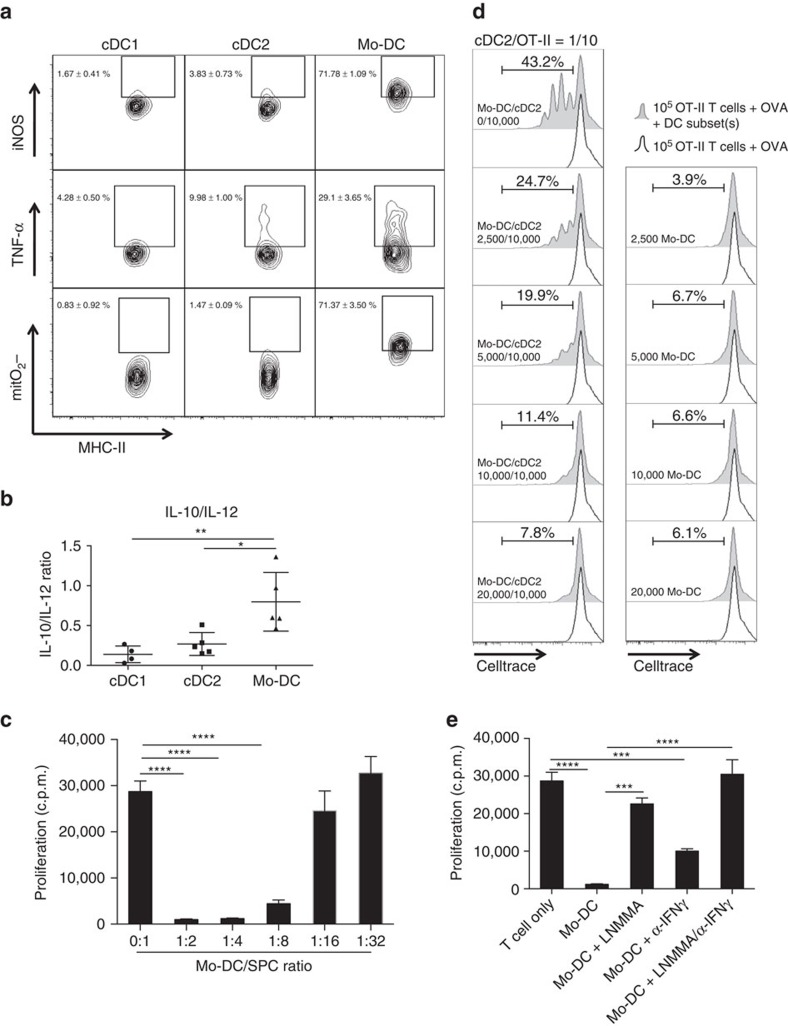
Mo-DCs display an immune suppressive TIP-DC phenotype. (**a**) Intracellular staining for iNOS, TNF-α and mitochondrial superoxide anion was performed on single-cell suspensions of 12-day-old 3LL-R tumours. *n*=4. (**b**) Supernatants of TADC subsets cultured for 48 h were tested for the presence of IL-10 and IL-12 by luminex. The graph depicts the IL-10/IL-12 ratio. *n*≥4. Statistical analysis by one-way ANOVA. *P<0.05; **P<0.01. (**c**) Mo-DCs were sorted from 12-day-old 3LL-R tumour single-cell suspensions and added at different ratios to splenocytes stimulated with anti-CD3 and anti-CD28 during 42 h and the proliferation of T cells was measured via ^3^H-thymidine incorporation (c.p.m.). Results are representative of two independent experiments with *n*=pool of 12 tumours. Statistical analysis by one-way ANOVA, ****P<0.0001. (**d**) Sorted Mo-DCs were added at increasing concentrations to 10^5^ OVA-stimulated OT-II T cells in the presence (left panels) or absence (right panels) of sorted cDC2s at a cDC2/OT-II ratio of 1/10. The histograms represent CellTrace dilution, indicative for T-cell proliferation. Black line=OVA-stimulated T cells without TADCs; shaded histogram=OVA-stimulated T cells in the presence of TADCs. *n*=pool of 12 tumours. (**e**) Sorted Mo-DCs were co-cultured with anti-CD3/CD28-stimulated splenocytes at a Mo-DC/SPC ratio of 1/4 with or without iNOS inhibitor (LNMMA) and/or α-IFN-γ. T-cell proliferation was measured via ^3^H-thymidine incorporation after 42 h (c.p.m.). *n*=pool of 12 tumours. Statistical analysis by one-way ANOVA, ***P<0.001; ****P<0.0001.

Tumour-associated Mo-DCs were consistently less efficient in activating naive antigen-specific T cells, in spite of their higher antigen uptake and processing capacity. Therefore, we wondered whether the Mo-DCs displayed features that could annihilate their T-cell stimulatory functions. We noted that Mo-DCs co-expressed high levels of tumour necrosis factor-α (TNF-α) and inducible nitric oxide synthase (iNOS) compared with both cDC subsets and hence displayed a phenotype reminiscent of inflammatory TNF-α and iNOS producing DCs (TIP-DCs) (Fig. 6a and Supplementary Fig. 7A). Moreover, these cells produced the highest levels of the inflammatory cytokines IL-6 and IL-1β, the monocyte and neutrophil attracting chemokines CCL2, CCL4 and CXCL1 and reactive oxygen species, such as the mitochondrial superoxide anion, of all TADC populations (Figs 5e and 6a and Supplementary Fig. 7B). In addition, they displayed the highest IL-10/IL-12 balance (Fig. 6b), a feature that is linked with a less immunogenic DC phenotype.

To directly assess whether tumour-derived Mo-DCs have the capacity to suppress T-cell responses, we in first instance polyclonally stimulated naive syngeneic splenocytes in the presence of increasing amounts of Mo-DCs. Mo-DCs dose-dependently suppressed T-cell proliferation (Fig. 6c), while cDC2s sorted from tumours or splenic cDCs did not (Supplementary Fig. 7C). Moreover, Mo-DCs were not able to induce OVA-stimulated OT-II T-cell proliferation at any concentration tested and strongly inhibited, in a concentration dependent way, cDC2-driven OVA-stimulated OT-II T-cell proliferation (Fig. 6d).

Importantly, higher iNOS expression might result in a higher NO production, which is reported to be a potential T-cell suppressive molecule^36^,^37^. Mo-DCs sorted from LLC-OVA tumours were co-cultured with polyclonally activated splenocytes in the presence of the iNOS inhibitor L-NMMA (Fig. 6e). T-cell proliferation was significantly enhanced under these conditions, demonstrating an active NO-mediated T-cell suppressive activity by Mo-DCs. The TIP-DC phenotype, including iNOS expression, was shown before to depend on IFN-γ^38^. The addition of blocking anti-IFN-γ antibodies to the Mo-DC/SPC cultures indeed also increased T-cell proliferation, although to a lesser extent than iNOS inhibition. Similar findings were obtained upon co-culture of LLC-OVA-derived Mo-DC with CFSE-labelled CD8^+^ OT-I T cells (Supplementary Fig. 7D). Hence, Mo-DCs display an immune suppressive TIP-DC phenotype that precludes the potent activation of T cells.

### Both tumour cDC subsets migrate to draining lymph nodes

**Figure 7:**
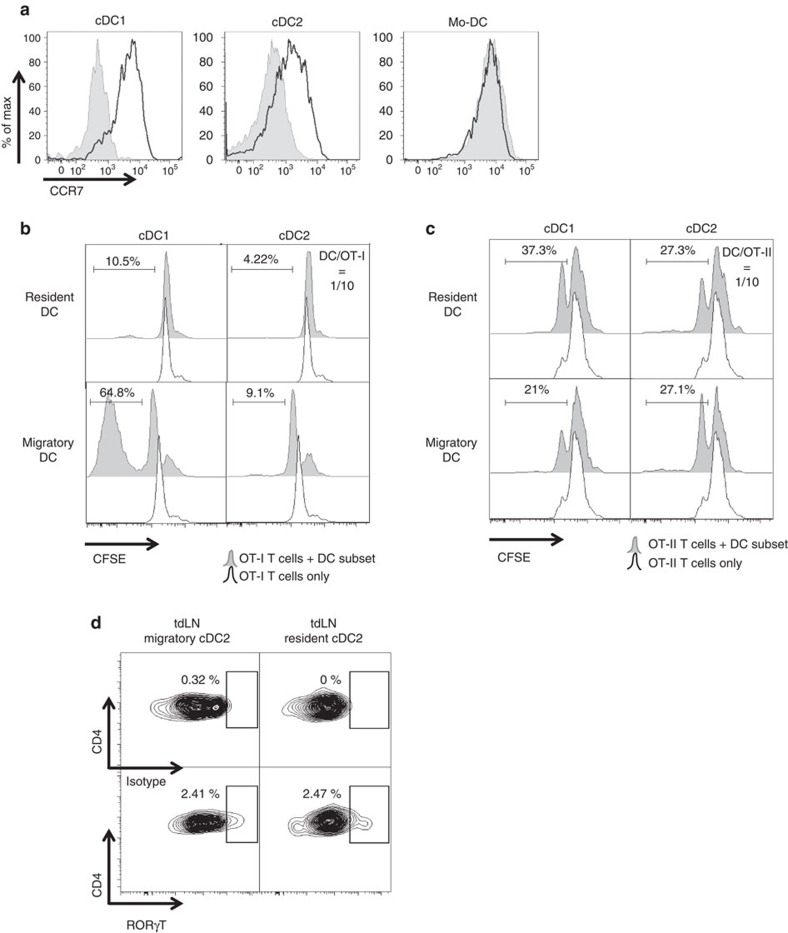
Both tumour-associated cDC subsets migrate to tumour draining lymph nodes and differentially activate CD8^+^ and CD4^+^ T cells. (**a**) Single-cell suspensions of 12-day-old 3LL-R tumours were stained for CCR7 and histogram overlays are shown. Black line=expression of CCR7; shaded histogram=isotype control. Results are representative of three independent experiments with *n*=4. (**b**,**c**) The indicated amount of sorted DC subsets from tumour-draining lymph nodes were co-cultured for 3 days with 10^5^ purified CD8^+^ OT-I T cells (**b**) or CD4^+^ OT-II T cells (**c**). The histograms represent CFSE dilution, indicative for T-cell proliferation. Black line=non-stimulated T cells without TADCs; shaded histogram=T cells in the presence of TADCs. Results are representative of three independent experiments with *n*=pool of 10–12 tumours. (**d**) Intracellular staining on OT-II T cells co-cultered with sorted tumour-draining lymph nodes cDC2 subsets for 3 days at a DC/OT-II ratio of 1/10 was performed for the Th17-inducing transcription factor RORγt. Isotype control and transcription factor staining are depicted. *n*=pool of 10.

Tumour-associated cDCs possessed T-cell stimulating capacity, so we next wondered whether these cells were capable of migrating to the tumour-draining lymph nodes (tdLNs) and present tumour antigen. CCR7 expression, which is a prerequisite for DC migration to lymph nodes (LNs), was only present on the cDC subsets but not on Mo-DCs (Fig. 7a).

In the axillary and inguinal tdLNs (draining LNs for a subcutaneous tumour in the flank of the animal), cDC1 and cDC2 subsets, but not Mo-DCs, were present within the migratory DC population (Supplementary Fig. 8A), confirming the non-migratory character of Mo-DCs. Migratory and resident DCs were discriminated based on CD11c and MHC-II expression, as previously reported^39^,^40^. To assess whether these cDCs present tumour antigen, they were sorted from tdLNs of LLC-OVA tumour-bearing mice and co-cultured with CFSE-labelled CD8^+^ OT-I T cells or CD4^+^ OT-II T cells. Importantly, care was taken that no OVA^+^ cancer cells were present in the tdLN at the time of cDC sorting, as illustrated by the absence of OVA mRNA (Supplementary Fig. 8B). Migratory cDC1s, but not cDC2s, strongly stimulated OT-I proliferation (Fig. 7b), suggesting *in vivo* migration of SIINFEKL-loaded cDC1s from the tumour to the tdLNs. Notably, both resident cDC subsets were largely incapable of inducing OT-I T-cell proliferation (Fig. 7b). By contrast, both migratory cDC1s and cDC2s could stimulate OT-II T-cell proliferation (Fig. 7c). Unexpectedly, also the resident cDC1s and cDC2s induced OT-II proliferation (Fig. 7c), suggesting that migratory tumour-associated cDCs could transfer antigen to LN-resident cDCs populations for effective OT-II priming. Notably, similar to the tumour-associated cDC2s, tdLN migratory and resident cDC2s also induced RORγt in a fraction of the OT-II T cells (Fig. 7d).

Activated T cells may migrate from the tdLNs to the primary tumour where they are confronted with TADCs. To assess whether tumour-resident DCs influence the activity of Ag-experienced T cells, we co-cultured TADCs from LLC-OVA tumours with sorted CD4^+^ or CD8^+^ T cells from the tdLNs of 12-day-old LLC-OVA-bearing mice. Interestingly, cDC2s were significantly better in triggering T-cell proliferation of *in vivo* primed T cells than Mo-DCs (Supplementary Fig. 8C).

### cDC2s have beneficial effects when used for vaccination

**Figure 8:**
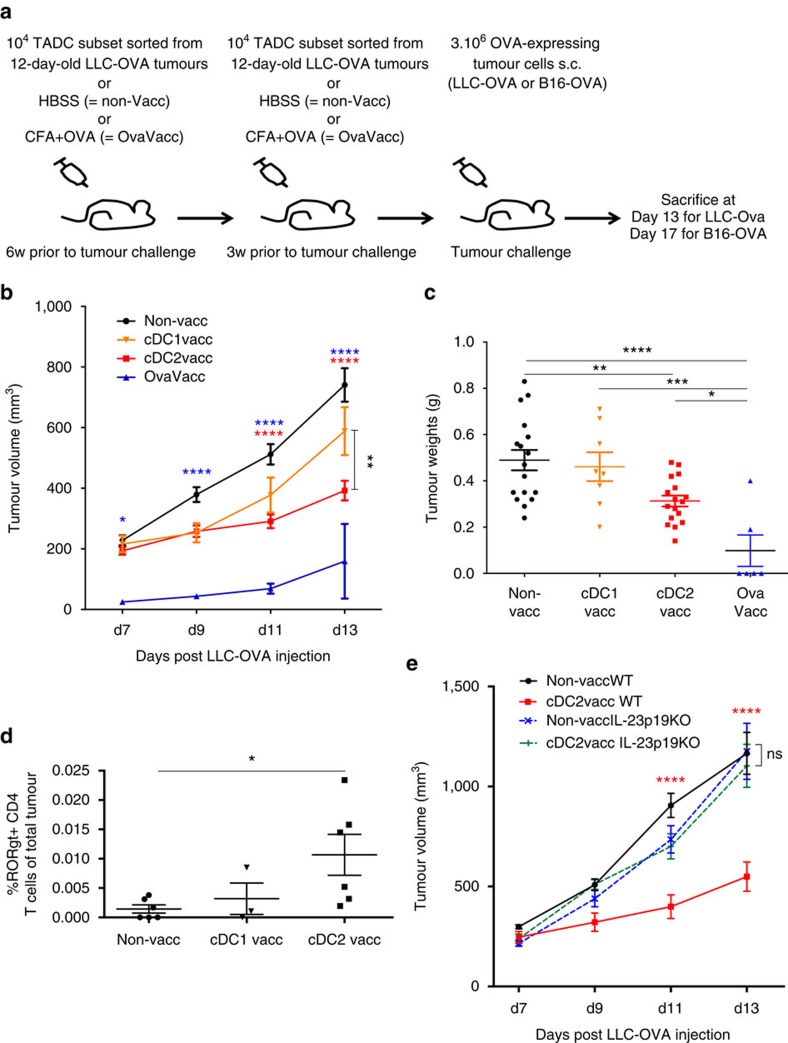
cDC2 vaccination is more beneficial than cDC1 vaccination in LLC-OVA tumour-bearing mice and induces a Th17 phenotype. (**a**) Schematic representation of the vaccination protocol. (**b**,**c**) Growth curve (**b**) and tumour weights (**c**) of LLC-OVA tumours after vaccination with LLC-OVA TADC subsets. (**d**) Percentages RORγt^+^ CD4^+^ T cells in LLC-OVA tumours after vaccination with LLC-OVA TADC subsets following the protocol depicted in (**a**). For all experiments, results are representative of two independent experiments with *n*=4–15 tumours. (**e**) Growth curve of LLC-OVA tumours after vaccination with LLC-OVA derived cDC2s in WT or IL-23p19 KO mice. *n*=8. Statistical analysis by one-way ANOVA. *P<0.05; **P<0.01; ***P<0.001; ****P<0.0001.

Finally, to assess whether tumour-derived DC subsets could be used to elicit relevant immune memory responses in cancer, we set up vaccination experiments as depicted in Fig. 8a. Importantly, the sorted TADC subsets used for the vaccination experiments were not *ex vivo* stimulated with cytokines, nor loaded with tumour antigen. Since Mo-DCs did not display LN migratory capacities and harboured immunosuppressive capacities (Fig. 6c,d), we decided to focus on the potential prophylactic effects of the cDC subsets.

Remarkably, upon challenge with LLC-OVA, only the cDC2-vaccinated mice had a significantly reduced tumour growth rate and weight compared with the non-vaccinated mice (Fig. 8b,c). However, a similar, though non-significant, trend was seen in the cDC1-vaccinated cohort (Fig. 8b). As expected, tumour growth in OVA-vaccinated mice was strongly retarded or inhibited. Slower tumour growth correlated with a slightly increased presence of CD8^+^ T cells in tumours of both vaccinated cohorts, which, however, did not reach the significantly higher levels seen in the OVA-vaccinated mice (Supplementary Fig. 9A). Remarkably, using H2K^b^/SIINFEKL dextramer staining, the proportion of tumour antigen-specific CD8^+^ T cells within the total CD8^+^ T-cell population was significantly increased in OVA-vaccinated mice and a trend was visible in cDC1-vaccinated, but not in cDC2-vaccinated mice (Supplementary Fig. 9B). These data are in line with the superior CTL-stimulatory capacity of cDC1s, and suggest that the stronger anti-tumour effect of cDC2 vaccination is mediated by other changes in the tumour microenvironment.

In this respect, tumour- or tdLN-derived cDC2s, but not cDC1s, were shown to stimulate Th17 cells *in vitro* (Figs 5c and 7d). In line with these data, the percentage of RORγt^+^ CD4^+^ tumour-infiltrating lymphocytes (TILs) was only significantly increased in tumours from cDC2-vaccinated mice, without an increase in the overall proportion of CD4^+^ T cells (Fig. 8d and Supplementary Fig. 9C). No changes could be observed in the amount of Foxp3^+^ Treg, T-bet^+^ Th1 or GATA3^+^ Th2 CD4^+^ TIL after vaccination, in any condition (Supplementary Fig. 9D–F). Th17 cells were crucially involved in the anti-tumour effect of cDC2 vaccination as this effect was totally abrogated in IL-23p19-deficient mice, which lack Th17 cells^41^ (Supplementary Figs 8E and 10B). Accordingly, CD4^+^ T-cell depletion reversed the cDC2-mediated protection (Supplementary Fig. S10A–C). This is remarkable, since CD4^+^ T-cell depletion lowers tumour growth in non-vaccinated mice, indicating that cDC2 vaccination switches the CD4^+^ T-cell pool from mainly pro-tumoral to mainly anti-tumoral. Notably, also CD8^+^ T-cell depletion partly reversed the cDC2 vaccination effect (Supplementary Fig. 10A,B,D), which is in accordance with the enhanced activation state (as shown by enhanced IFNγ production) by these cells in tumours from vaccinated versus non-vaccinated mice (Supplementary Fig. 10E). Depleting NK and NKT cells with an anti-NK1.1 mAb did not influence tumour growth (Supplementary Fig. 11A–C), despite a slight but significant increase in the NK (but not NKT) cell numbers (Supplementary Fig. 11D). However, IFNγ production by NK cells was unaffected by cDC2 vaccination (Supplementary Fig. 11E).

**Figure 9:**
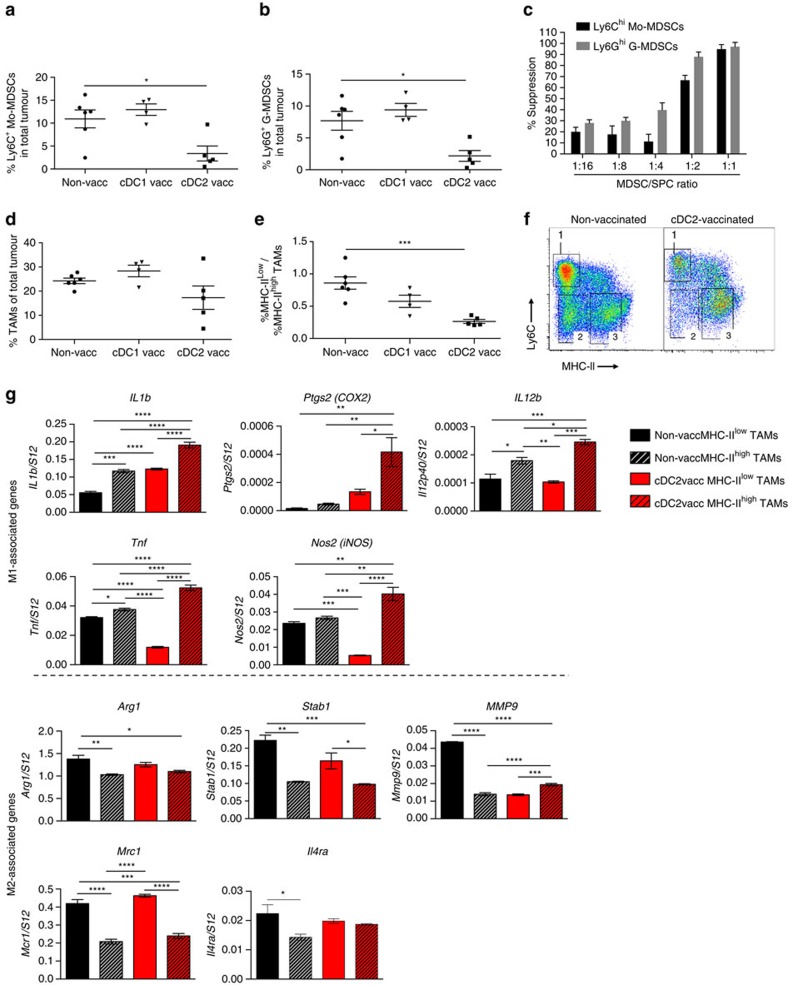
cDC2 vaccination reduces the MDSC infiltrate and reprograms TAMs from a protumoral M2-like to an M1-like phenotype. (**a**,**b**) Percentages of Mo-MDSCs (**a**) and G-MDSCs (**b**) in LLC-OVA tumours after vaccination with LLC-OVA TADC subsets following the protocol depicted in Fig. 8a. Results are representative of two independent experiments with *n*=4–15 tumours. Statistical analysis by one-way ANOVA. *P<0.05. (**c**) CD11b^+^ Ly6C^hi^ Ly6G^−^ Mo-MDSCs and CD11b^+^ Ly6C^int^ Ly6G^+^ G-MDSCs were sorted from 12-day-old 3LL-R tumour single-cell suspensions and added at different ratios to anti-CD3-stimulated splenocytes during 42 h and the proliferation T cells was measured via ^3^H-thymidine incorporation (c.p.m.). Results are representative of three independent experiments with *n*=pool of 6 tumours. (**d**,**e**) Percentages of CD11b^+^ Ly6G^+^ Ly6C^−^ TAMs (**d**) and the ratio of M2-like MHC-II^low^ TAMs/M1-like MHC-II^high^ TAMs (**e**) in LLC-OVA tumours after vaccination with LLC-OVA TADC subsets following the protocol depicted in Fig. 8a. Results are representative of two independent experiments with *n*=4–15 tumours. Statistical analysis by one-way ANOVA. ***P<0.001. (**f**) Representative plot of LLC-OVA tumours of cDC2- or HBSS-vaccinated mice gated on CD11b^+^ Ly6G^-^ single cells, showing (1) Ly6C^+^monocytes, (2) MHC-II^low^ TAMs and (3) MHC-II^high^ TAMs. (**g**) Expression of indicated M1 and M2 associated genes in sorted MHC-II^low^ and MHC-II^high^ TAM subsets of LLC-OVA tumour-bearing mice vaccinated with cDC2 or HBSS was assessed using qRT-PCR. The expression was normalized based on the S12 housekeeping gene. *n*=pool of 6 tumours. Statistical analysis by one-way ANOVA. *P<0.05; **P<0.01; ***P<0.001; ****P<0.0001.

In multiple tumour models, including LLC, tumour growth is not only regulated by TILs, but also by the phenotype of tumour-associated myeloid cells such as myeloid-derived suppressor cells (MDSCs) and TAMs. Interestingly, the presence of CD11b^hi^ Ly6C^hi^ MHC-II^neg^ Ly6G^neg^ monocytic cells and CD11b^hi^ Ly6C^int^ MHC-II^neg^ Ly6G^hi^ granulocytic cells is significantly reduced in tumours of cDC2-vaccinated mice as compared with non-vaccinated and cDC1-vaccinated cohorts (Fig. 9a,b). To assess whether these cells possess T-cell suppressive capacity, which would classify them as monocytic and granulocytic MDSCs, they were FACS sorted from tumour single-cell suspensions and added at different ratios to polyclonally stimulated splenocytes. As shown in Fig. 9c, these cells strongly suppress T-cell proliferation. Hence, cDC2 vaccination strongly reduces the presence of MDSCs in tumours. Also M2-oriented TAMs promote tumour progression. Overall CD11b^hi^ Ly6C^lo^ Ly6G^neg^ TAM numbers only showed a trend towards a reduction in cDC2-vaccinated mice (Fig. 9d). Importantly, within the TAM compartment, cDC2 vaccination caused a shift towards more M1-like MHC-II^high^ TAMs (i.e. a lower MHC-II^low^/MHC-II^high^ TAM ratio) (Fig. 9e,f). In addition, these MHC-II^high^ TAM had a more pronounced M1 phenotype as compared with those from non-vaccinated animals, as evidenced by a further upregulated expression of M1-associated genes, while most M2-associated genes (except for *Mmp9*) did not significantly change (Fig. 9g). Notably, the few remaining MHC-II^low^ TAMs from cDC2-vaccinated mice also altered their M1 gene expression profile, with some genes being upregulated (*Il1b, Ptgs2, Il12p40*) and others downregulated (*Tnf, Nos2*). M2 genes in these cells remained mostly unchanged. Overall, these data show that the myeloid compartment of LLC-OVA tumours from cDC2-vaccinated mice is dominated by strongly M1-oriented TAMs.

**Figure 10:**
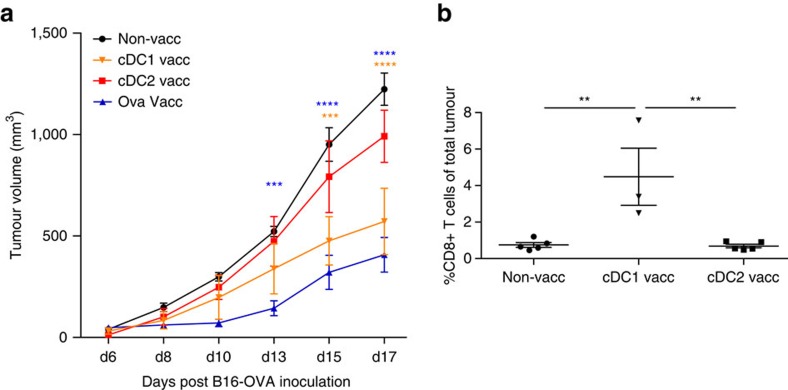
cDC1 vaccination is more effective than cDC2 vaccination in B16 melanoma tumour-bearing mice. (**a**,**b**) Growth curve of B16-OVA tumours (**a**) and percentages of CD8^+^ T cells in B16-OVA tumours (**b**) after vaccination with LLC-OVA TADC subsets following the protocol depicted in Fig. 8a. *n*=4–6 tumours. Statistical analysis by one-way ANOVA. **P<0.01; ***P<0.001; ****P<0.0001.

Finally, we turned to the B16-OVA tumour model, in which TAMs are present in very low numbers and are mainly M1-like MHC-II^high^ polarized (Fig. 2c). In this model, antitumour effects are more likely to be directly mediated by cytotoxic T cells without an overt interference of tumour-infiltrating myeloid cells. Indeed, vaccination with cDC1s isolated from LLC-OVA tumours conferred a better protection than cDC2 vaccination (Fig. 10a), which correlated with a significantly augmented infiltration of CD8^+^ T cells in the former (Fig. 10b). Hence, depending on the role of TAMs or TILs in tumour immunity, different tumour-derived conventional TADC subsets could be exploited to develop personalized DC adoptive immunotherapies.

## Discussion

DCs are a heterogeneous population of cells and the coexistence of developmentally and functionally distinct DC subsets was reported in different organs, such as the skin, intestine and lung^11^,^12^,^13^. Using precursor transfer experiments and Flt3L- and CCR2-deficient mice, we demonstrated for the first time the coexistence of three developmentally distinct TADC subsets: pre-cDC-derived cDC1s, pre-cDC-derived cDC2s and monocyte-derived Mo-DCs. The dissection of MHC-II^high^ CD11c^+^ CD11b^+^ cells in cDC2s and Mo-DCs might explain the slight expression of the monocyte/macrophage related markers CD64 and MerTK in the ontogenically mixed CD11b^+^ TADC population described by Broz *et al*.^24^

Moreover, using markers confirmed by previously published transcriptome analysis defining human inflammatory monocyte derived DCs^33^, the coexistence of cDC1s, cDC2s and Mo-DCs was suggested in freshly isolated tumour biopsies of human lung and colorectal cancer patients, indicating the therapeutic relevance of dissecting the DC compartment in tumours.

Since TAMs make up the bulk of the monocyte-derived intratumoral cells, the question arises which microenvironmental cues instruct a monocyte to become a macrophage-like or a DC-like cell. In this context, it is interesting to note that tumours containing higher amounts of Mo-DCs were also infiltrated with a higher amount of MHC-II^high^ M1-like TAMs, at the expense of MHC-II^low^ M2-like TAMs, suggesting that these differentiation pathways employ overlapping signals^25^,^26^. In the case of Mo-DCs, Flt3L and GM-CSFR appear both instrumental for their differentiation, while MHC-II^high^ TAMs are influenced by GM-CSFR^42^, but not Flt3L. The involvement of GM-CSFR in the generation of tumour-associated Mo-DCs is remarkable, as it disagrees with the GM-CSFR-independent generation of inflammatory monocyte-derived DCs in infectious models of acute inflammation^17^. This may be explained by the more chronic, smoldering type of inflammation in the tumour microenvironment, or, alternatively, Mo-DC generation may depend on the presence of cDCs. In this scenario, Flt3L and GM-CSFR would contribute to cDC generation, which subsequently instruct monocyte-to-Mo-DC differentiation. Finally, the presence of cDC1s and cDC2s seemed dependent on a combination of both Flt3L and GM-CSFR signalling. In this regard, it was previously shown that the combined loss of GM-CSF and Flt3L generates a much greater decrease in cDCs than the loss of Flt3L alone^29^.

Intracellular staining for IRF4 and IRF8 further corroborated the heterogeneity of the TADC compartment. High levels of IRF8 were detected in tumour-associated cDC1s and of IRF4 in tumour-associated cDC2s, suggesting their kinship to cDC1s and cDC2s found in several organs, respectively^30^. IRF8 also regulates the inflammatory profile of M1 macrophages^43^, but inflammatory Mo-DCs only express intermediate levels of IRF8, as well as IRF4, suggestive of other transcription factor(s) stimulating Mo-DC development.

It was previously described that TADCs differ at the functional level at different phases of tumour growth^7^. Here we showed that the relative proportions of distinct TADC subsets evolve during tumour progression, which could at least partly explain a change in function over time. Indeed, although all TADC subsets express high levels of costimulatory molecules and intrinsically possess the capacity to activate naive T cells in an MLR, Mo-DCs outcompeted cDCs for antigen uptake, both *in vitro* and *in vivo*. However, Mo-DCs sorted from LLC-OVA tumours were inefficient inducers of naive OVA-specific CD8^+^ and CD4^+^ T-cell proliferation. In fact, they efficiently suppressed polyclonally- or OVA-stimulated T-cell proliferation. This could be linked to their tolerogenic IL-10^high^ TIP-DC phenotype and high production of immune suppressive molecules, such as NO and the mitochondrial superoxide anion. Moreover, Mo-DCs, unlike both cDC subsets, failed to upregulate CCR7 in tumours. Hence, Mo-DCs efficiently scavenge tumour antigen, but are unable to migrate to the tdLN, and contribute to an immunosuppressive environment at the tumour site.

Conversely, we could show that cDCs sorted from tdLNs of LLC-OVA tumour-bearing mice could stimulate OVA-specific CD8^+^ and CD4^+^ T-cell responses, indicative for *in vivo* migration of antigen-loaded cDCs from the tumour to the tdLNs, in line with the high CCR7 expression of these cells and as recently shown in other models^44^. Notably, cDC2s, either isolated from the tumour site or from tdLNs, were unique in their Th17-inducing capacity, which is likely to be linked with their high production of the Th17-inducing cytokines IL-23, IL-1β and IL-6. The Th17-inducing capacity of this DC subset has also been reported in the mucosa^21^,^23^. Hence, tumours contain both suppressive Mo-DCs and fully functional, mature cDC subsets, the latter being of interest for therapeutic purposes.

Throughout the past decade, the use of patient-derived DCs as a means to elicit therapeutically relevant immune responses in cancer patients has been extensively investigated. However, DCs used for vaccination are commonly generated from peripheral blood monocytes and might thus be reprogrammed by the tumour microenvironment into suppressive Mo-DCs^45^,^46^. To assess whether tumour-associated cDC subsets could be amenable to elicit relevant immune memory responses in cancer, we isolated these cells from LLC-OVA tumours to immunize mice, without *ex vivo* stimulation or antigen loading of the sorted tumour-derived cDCs, prior to a challenge with LLC-OVA or B16-OVA. LLC tumours are heavily infiltrated by M2-oriented TAMs, which are mainly recruited to hypoxic tumour areas and prohibit anti-tumour immunity^25^,^26^. Reprogramming of these macrophages, for example, by preventing their entry into hypoxic sites has been shown to initiate a cascade of anti-tumour events resulting in reduced tumour growth^47^. B16 tumours, on the other hand, are poorly infiltrated by myeloid cells, whose contribution to tumour growth is rather minor^48^. Interestingly, cDC2 vaccination led to a reduced LLC-OVA, but not B16-OVA, tumour growth paralleled by an increased infiltration of RORγt^+^ CD4^+^ T cells, a reduction of monocytic and granulocytic MDSCs and a reprogramming of TAMs from a protumoral M2-like MHC-II^low^ to an antitumoral M1-like MHC-II^high^ phenotype. Th17 cells are crucial for this antitumoral effect of cDC2 vaccination, but the exact downstream mechanisms still need elucidation. The Th17-associated cytokine IL-21 was reported to shift TAMs from M2-like to M1-like cells in breast tumours^49^, but effects of IL-17 on TAM polarization were not reported. Interestingly, GM-CSF was recently shown to regulate the M1-like phenotype of TAMs^50^ and is coming to the forefront as a Th17 signature cytokine^51^,^52^. Notably, cDC2 vaccination does not increase the number of SIINFEKL-specific CTL in tumours compared with non-vaccinated mice, but increases the IFNγ production by these cells, explaining the partial contribution of CD8^+^ T cells to the success of vaccination. Interestingly, cDC2 vaccination does not significantly influence B16-OVA tumour growth, the control of which may be more strictly dependent on OVA-specific CTLs. In agreement with the antitumoral immune responses induced by cDC1s in melanomas^53^, our cDC1 vaccination increases the proportion of intratumoral CTLs resulting in a significant reduction of B16-OVA tumour growth. The lack of effect on LLC-OVA tumours is likely due to the dominance of immunosuppressive cell types, such as M2-like TAMs and MDSCs, in the environment. The proposed vaccination strategy holds promise as a beneficial follow-up therapy, where genuine tumour-derived cDCs could be sorted from postoperative, primary tumour tissue. Of note, the absolute number of TADCs required in our vaccination approach was very low (10^4^ cells) compared with similar reports applying DC vaccination (with 10^5^ to 3 × 10^6^ cells), as either prophylactic or therapeutic strategies^54^,^55^,^56^,^57^. Moreover, unlike most DC-based anti-tumour approaches currently under investigation, knowledge of the immunodominant tumour antigen is no prerequisite for this TADC vaccination strategy.

In conclusion, we unraveled the coexistence of ontogenically and functionally distinct TADC subsets, which can facilitate the design of therapeutic interventions targeted at specific TADC subsets. In particular, cDC1s are needed to boost anti-tumour CTLs, which may not work efficiently in tumours dominated by MDSCs and M2-oriented TAMs. In this case, cDC2s may help in eliminating MDSCs and shifting the TAM phenotype from M2-like to M1-like.

## Methods

### Mouse strains

Female Balb/c, CD45.2 and CD45.1 C57BL/6 mice were from Janvier. Ubiquitin-GFP mice were purchased from Jackson. *Csf2rb*^−/−^, *Flt3l*^−/−^, *Ccr2*^−/−^, *Il23a*^−/−^ and MMTV-PyMT mice were provided by Melanie Greter (University of Zurich, Germany), Bart Lambrecht (VIB-UGent, Belgium), Frank Tacke (Aachen University, Germany), Véronique Flamand (Université Libre de Bruxelles, Belgium) and Massimiliano Mazzone (VIB-KULeuven, Leuven, Belgium) respectively.

All procedures followed the guidelines of the Belgian Council for Laboratory Animal Science and were approved by the Ethical Committee for Animal Experiments of the Vrije Universiteit Brussel (licenses 11-220-3 and 15-220-2).

### Cell cultures and tumour models

LLC was purchased from the ATCC Cell Biology Collection. The 3LL-R variant was generated in house by sequential exposure of C57BL/6 Lewis Lung carcinoma (3LL cells) to LPS/LK-activated peritoneal macrophages *in vitro* and correlated with resistance towards TNF-α and an increased tumorigenicity on s.c. inoculation, while the 3LL-S variant represented a clone from the parental 3LL, which in contrast was highly sensitive for macrophage-mediated cytotoxicity^32^. LLC-OVA, MC38, B16-OVA and T241 cells were kind gifts from Dmitry Gabrilovich (The Wistar Institute, Philadelphia, USA), Massimiliano Mazzone (VIB-KULeuven), Karine Breckpot (Vrije Universiteit Brussel, Brussels, Belgium) and Lena Claesson-Welsh (University of Uppsala, Uppsala, Sweden), respectively.

LLC-OVA, 3LL-R and 3LL-S cell lines were maintained in Roswell Park Memorial Institute-1640 medium (RPMI; Sigma) supplemented with 10% (v/v) heat-inactivated fetal calf serum (FCS; Gibco), 300 μg ml^−1^ L-glutamine (Gibco), 100 units ml^−1^ penicillin and 100 μg ml^−1^ streptomycin (Gibco) and monthly tested for the presence of mycoplasma. For MC38, B16-OVA and T241 cultures, RPMI was replaced by Dulbecco's modified Eagle's medium (Sigma).

LLC, LLC-OVA, 3LL-R, 3LL-S lung carcinoma cells, MC38 colon carcinoma cells, B16-OVA melanoma cells and T241 ficrosarcoma cells were harvested and single-cell suspensions of 3 × 10^6^ in 200 μl of phosphate-buffered saline (PBS) were injected subcutaneously into the right flank of syngeneic 6- to 9-week-old female C57Bl/6 mice. Female MMTV-PyMT mice developed mammary tumours spontaneously.

For *ex vivo* TADC and T-cell cultures, this medium was supplemented with 1 mM non-essential amino acids (Invitrogen), 1 mM sodium pyruvate (Invitrogen) and 0.02 mM 2-mercapto ethanol (Invitrogen).

For intrathoracic 3LL-R injections, 5 × 10^5^ 3LL-R carcinoma cells were harvested and resuspended together with 25 μg Matrigel in 50 μl PBS. Cell suspensions were kept on ice until injection. Mice were anaesthetized and placed in the left lateral decubitus position. One-millilitre tuberculin syringes with 30-gauge hypodermic needles were used to inject the cell inoculum percutaneously into the right lateral thorax, at the lateral dorsal axillary line, approximately 1.5 cm above the lower rib line just below the inferior border of the scapula. The needle was quickly advanced 6 mm into the thorax and was quickly removed after the injection. After tumour injection, the mouse was turned to the right lateral decubitus position. At day 7, mice were killed and lung tissue and lung tumours were removed.

For trans-anal rectal cancer injections, mice were anaesthetized with a 1/10 Nembutal dilution. The anal orifice was gently enlarged with a blunt-tipped forceps. In case of feces present, the colon was rinsed with saline using a flexible catheter. MC38 cells were injected submucosally into the distal posterior rectum at concentrations of 2.5 × 10^5^ per 50 μl PBS with a 29-gauge syringe. After the injection, the syringe was kept in a position for a few seconds, to prevent back flow. After 4 weeks, mice were killed and tumours were carefully removed.

Tumour volumes were determined by caliper measurements and calculated using the formula: *V*=*π* × (*d*^2^ × *D*)/6, where *d* is the minor tumour axis and *D* is the major tumour axis.

### Lung cancer and colorectal cancer patients

We enrolled four non-small-cell lung carcinoma (NSCLC) patients who were not subjected to neo-adjuvant chemotherapy, including two males (68 and 67 years of age) with pT2aN1M0 (stage IIA) spinocellular carcinoma and cT1bN0M0 (stage IA) carcinoma and two females (67 and 59 years of age) with pT2aN0M0 (stage IB) adenocarcinoma and pT2aN1M0 (stage IIA) spinocellular carcinoma. The four colorectal cancer patients enrolled were not subjected to chemotherapy and included three males (71and 62 years of age) with pT3N0 adenocarcinomas, one male (79 years of age) with stage I T2M0N0 adenocarcinoma and one female (49 years of age) with stage IV adenocarcinoma. All protocols were approved by the Ethics Committee of the University Hospitals Gasthuisberg (Leuven, Belgium), and all subjects gave written informed consent before study participation (licenses S50623 for NSCLC and S58730 for CRC).

### *Ex vivo* single-cell preparation

Subcutaneous, orthotopic or MMTV-PyMT tumours were excised, cut in small pieces, treated with 10 U ml^−1^ collagenase I, 400 U ml^−1^ collagenase IV and 30 U ml^−1^ DNaseI (Worthington) for 30 min at 37 °C, squashed and filtered. Red blood cells were removed using erythrocyte lysis buffer and density gradients (Axis-Shield) were used to remove debris and dead cells.

Tumour-draining LNs were cut, dissociated with 10 U ml^−1^ collagenase I, 400 U ml^−1^ collagenase IV and 30 U mL^−1^ DNaseI (Worthington) for 45 min at 37 °C and filtered.

Spleens were flushed with 200 U ml^−1^ collagenase III (Worthington) and left for 30 min at 37 °C. Afterwards, spleens were filtered and red blood cells were removed using erythrocyte lysis buffer.

To purify DC subpopulations from tumour, spleen or LNs, CD11c^+^ cells were MACS-enriched (anti-CD11c microbeads; Miltenyi) and sorted using BD FACSAria II (BD Biosciences) according to the gating strategy in Fig. 1a, Supplementary Fig. 6A or Supplementary Fig. 8A, respectively.

Bone marrow leukocytes were isolated through flushing of tibia and femur. The obtained cell suspensions were filtered, and red blood cells were removed using erythrocyte lysis buffer. To purify bone marrow monocytes, CD11b^+^ cells were MACS-enriched (anti-CD11b microbeads; Miltenyi) before sorting.

### Flow cytometry and cell sorting of mouse samples

Commercial antibodies for cell surface stainings are listed in Supplementary Table 1. To prevent aspecific binding, cells were pre-incubated with rat anti-mouse CD16/CD32 (clone 2.4G2; BD Biosciences).

Normalized delta-Median Fluorescence Intensity (ΔMFI) was calculated as: ((MFI staining)-(MFI isotype staining))/(MFI staining). FACS data were acquired using a BD FACSCanto II or LSRII (both from BD Biosciences) and analysed using FlowJo (Tree Star, Inc.). To purify TADCs, cells were sorted using a BD FACSAria II (BD Biosciences) from 9 to 15 pooled tumours.

### Flow cytometry of lung cancer and colorectal cancer patients

Lung tumour biopsies, healthy lung tissues or colorectal tumour biopsies were minced in RPMI medium containing 0.1% collagenase type I, 0.2% dispase type I and DNase I 100 U ml^−1^ (60 min at 37 °C), passed through a 19-gauge needle and passed through a 70 and 40 μm cell strainer. After erythrocyte lysis, cells were resuspended in FACS buffer (PBS containing 2% FBS and 2 mM EDTA) and counted.

Fc receptors were blocked using Human Fc block reagent (Miltenyi) for 15 min at 4 °C. After washing, samples were stained with biotinylated anti-BDCA-1 antibody (Miltenyi) (1/10) and fixable viability dye eFluor506 (eBioscience) (1/200) for 30' at 4 °C in the dark. Subsequently, samples were washed and stained with FITC-labelled anti-CD3 (eBioscience) (1/50), anti-CD19 (BD Biosciences) (1/20) and anti-CD56 (eBioscience) (1/10), PE-labelled anti-BDCA-2 (Miltenyi) (1/50), PE-TexasRed-labelled anti-CD16 (Invitrogen) (1/100), PeCy7-labelled anti-CD11c (1/10), AF700-labelled anti-CD45 (BD Biosciences) (1/20), APC-Cy7-labelled anti-HLA-DR (eBioscience) (1/100), Pacific Blue-labelled anti-CD14 (Invitrogen) (1/100), BV605-labelled anti-CD11b (BD Biosciences) (1/200), APC-labelled anti-BDCA-3 and BV786-labelled streptavidin (BD Biosciences) (1/200) for 30′ at 4 °C in the dark.

Samples were washed and fixed for 45′ at room temperature (RT) using 200 μl of Fix/Perm per sample (FoxP3/Transcription factor staining buffer set; eBioscience). Cells were washed twice in Perm buffer and stained with PerCP-Cy5.5-labelled anti-IRF8 (eBioscience) (1/400) and unlabelled anti-IRF4 (Santa Cruz) (1/400) for 30′ at RT in the dark in Perm buffer with Human Fc block reagent (Miltenyi) (1/20). After washing with Perm buffer, samples were stained with AF647-conjugated donkey anti-goat (Molecular Probes, Carslbad, CA, USA) (1/1000) for 30′ at RT in the dark. Cells were washed with FACS buffer and measured directly on a BD LSRII flow cytometer (BD Biosciences) or kept overnight at 4 °C. FACS data were analysed using FlowJo (Tree Star, Inc.).

### Adoptive pre-cDCs and monocyte transfers

Bone marrow Ly6C^hi^ monocytes and pre-cDCs were labelled with CellTrace Violet (Molecular Probes) and sorted from CD45.2 mice. 10^6^ Ly6C^hi^ monocytes or 4 × 10^5^ pre-cDCs were intravenously injected in 3LL-R tumour-bearing CD45.1 mice. The fate of the CD45.2^+^ CellTrace^+^ progeny was determined 72 h later.

### Cell depletion experiments

CD4^+^ T cell, CD8^+^ T cell and NK(T) cells were depleted during LLC-OVA tumour growth by intraperitoneal injection of 300 μg of anti-CD4 (GK1.5), 200 μg of anti-CD8 (YTS169) or 300 μg of anti-NK1.1 (PK136), respectively, every 2 to 3 days, starting 1 day prior to tumour inoculation.

### *In vitro* Ag uptake and phagocytosis assays

For *in vitro* Ag uptake, freshly isolated tumour single-cell suspensions were cultured in 96-well plates for 15 min at 4 or 37 °C, in the presence of ovalbumin fluorescently labelled with AF488 (Molecular Probes) at a final concentration of 10 μg ml^−1^. OVA uptake by TADCs was assessed via flow cytometry.

For *in vitro* latex uptake, freshly isolated tumour single-cell suspensions were cultured in 96-well plates for 40 min at 4 or 37 °C, in the presence of latex microspheres (Polysciences) diluted at 1:5,000. Latex uptake by TADCs was assessed via flow cytometry. For measuring *in vivo* latex uptake by TADCs, tumour-bearing mice were injected intravenously with 250 μl of yellow-green latex microspheres (Polysciences) diluted 1:25 in PBS. After 1–2 h, tumour single-cell suspensions were made and latex uptake by TADC subpopulations was assessed via flow cytometry.

### DQ-OVA processing experiments

To assess TADC antigen processing, tumour single-cell suspensions were incubated for 15 min at 0 or 37 °C in the presence of 10 μg ml^−1^ DQ-OVA (Molecular Probes), allowing antigen uptake. After thorough washing, cells could further process DQ-OVA intracellularly during different time intervals, at 0 or 37 °C. Following each time interval, cells were surface labelled and DQ-OVA fluorescence in each subset was measured via flow cytometry.

### Mixed leukocyte reaction

For mixed leukocyte reaction assays, T cells were purified from Balb/c splenocytes, by first immunodepleting CD11c^+^ and CD19^+^ cells using an MACS LD column with anti-CD11c and anti-CD19 microbeads (Miltenyi Biotech) and subsequently positively selecting CD4^+^ or CD8^+^ T cells using anti-CD4 or anti-CD8 microbeads (Miltenyi Biotech). 2 × 10^5^ purified Balb/c T cells were cultured in round-bottom 96-well plates with different concentrations of TAM or TADC sorted from 13-day-old 3LL-R tumours or C57Bl/6 splenic cDCs. Three days later, ^3^H-thymidine was added and cell proliferation was measured after another 18 h culture as counts per minute (c.p.m.) on a Wallac 1450 Liquid Scintillation Counter.

### Suppression assays and OT-I and OT-II T-cell activation

For OT-I and OT-II proliferation assays, MACS sorted CD11c^−^CD19^-^CD11b^−^CD4^−^CD8^+^ OT-I and CD11c^−^CD19^−^CD11b^−^CD8^−^CD4^+^ OT-II T cells were stained with 0.2 μM CFSE or 5 μM CellTrace Violet (Molecular Probes) following the manufacturer's instructions and cultured in flat-bottom 96-well plates. Purified TADCs or splenic cDCs were added to 10^5^ OT-I or OT-II T cells, which were stimulated with 1 μg ml^−1^ anti-CD3 and 2 μg ml^−1^ CD28 as a positive control. In case of antigen pulsing, 250 μg ml^−1^ of OVA was added. To inhibit iNOS the co-cultures were supplemented with 5 μM L-NMMA (*N*^G^-monomethyl-L-arginine; Alexis Biochemicals). After 72 h of co-incubation, proliferation of T cells was measured via CFSE dilution using flow cytometry.

For suppression assays, Mo-DCs or MDSCs were sorted from 12-day-old 3LL-R tumour single-cell suspensions and added at different ratios to splenocytes stimulated 1 day earlier with anti-CD3 (1 μg ml^−1^) and CD28 (2 μg ml^−1^) and cultured in flat-bottom 96-well plates. After 18 h ^3^H-thymidine was added and T-cell proliferation was measured after another 24 h of culture as c.p.m. on a Wallac 1450 Liquid Scintillation Counter.

For restimmulation of T cells sorted from tdLNs, CD4^+^ and CD8^+^ T cells were sorted from tdLN from 12-day-old LLC-OVA tumour-bearing mice. T cells were stained with 5 μM CellTrace Violet (Molecular Probes) following the manufacturer's instructions and were cultured in flat-bottom 96-well plates. 5 × 10^4^ LLC-OVA-purified TADCs were added to 10^5^ CD4^+^ or CD8^+^ T cells. After 18 h, ^3^H-thymidine was added and T-cell proliferation was measured after another 48 h of culture as c.p.m. on a Wallac 1450 Liquid Scintillation Counter.

### Measurement of cytokine and chemokine production

Cytokine and chemokine concentrations were measured by Bio-Plex (Bio-Rad), according to the supplier's protocols.

### TADC vaccination experiments

For vaccination experiments, naive C57Bl/6 mice were subcutaneously injected with 10^4^ TADCs of a specific subset, 6 and 3 weeks prior to subcutaneous LLC-OVA or B16-OVA inoculation. TADCs were sorted from a pool of 10–12 LLC-OVA tumour-bearing mice. Mice vaccinated subcutaneously with 100 μg OVA protein in 100 μl CFA were used as positive controls.

### RNA extraction and cDNA preparation for qPCR

RNA was extracted using TRIzol (Invitrogen) and was reverse-transcribed with oligo(dT) and SuperScript II RT (Invitrogen). Quantitative real-time PCR was performed in an iCycler, with iQ SYBR Green Supermix (Bio-Rad). Primer sequences are listed in Supplementary Table 2. PCR cycles consisted of 1′ 94 °C, 45″ 55 °C, 1′ 72 °C. Gene expression was normalized using ribosomal protein S12 (*Mrps12*) as a housekeeping gene. Primers are listed in Supplementary Table 2.

### Statistics

Significance was determined by the Student's *t*-test or analysis of variance followed by a post test using GraphPad Prism 6.0 software. A *P*-value <0.05 was considered statistically significant. All graphs show mean±s.e.m.

### Data availability

Data supporting the findings of this study are available within the article and its supplementary information files are from the corresponding author upon reasonable request.

## Additional information

**How to cite this article:** Laoui, D. *et al*. The tumour microenvironment harbours ontogenically distinct dendritic cell populations with opposing effects on tumour immunity. *Nat. Commun.* **7,** 13720 doi: 10.1038/ncomms13720 (2016).

**Publisher's note:** Springer Nature remains neutral with regard to jurisdictional claims in published maps and institutional affiliations.

## Supplementary Material

Supplementary Information Supplementary Figures 1-11 and Supplementary Tables 1-2

Peer Review
