# Supplementary material for: The tumour microenvironment harbours ontogenically distinct dendritic cell populations with opposing effects on tumour immunity
Source: Nat Commun. 2016 Dec 23;7:13720. doi: 10.1038/ncomms13720 (PMC5196231; doi:10.1038/ncomms13720)
Supplement: Supplementary Information — Supplementary Figures 1-11 and Supplementary Tables 1-2 [file ncomms13720-s1.pdf]

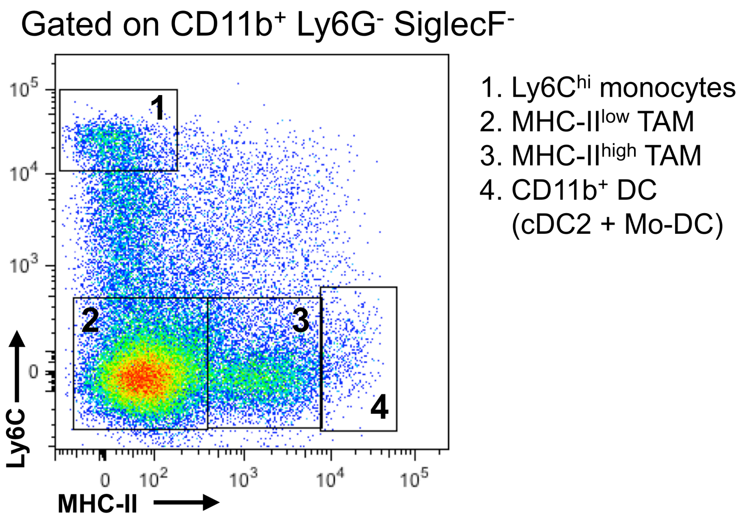

Supplementary figure 1, related to main figure 1. Gating of monocytes and tumor-associated macrophages in 3LL-R lung carcinoma

Representative plot of 12-day old 3LL-R tumor gated on CD11b^{+} Ly6G^{-} SiglecF^{-} cells, showing a high infiltration of (1) Ly6C^{high} monocytes, (2) MHC-II^{low} and (3) MHC-II^{high} TAM.

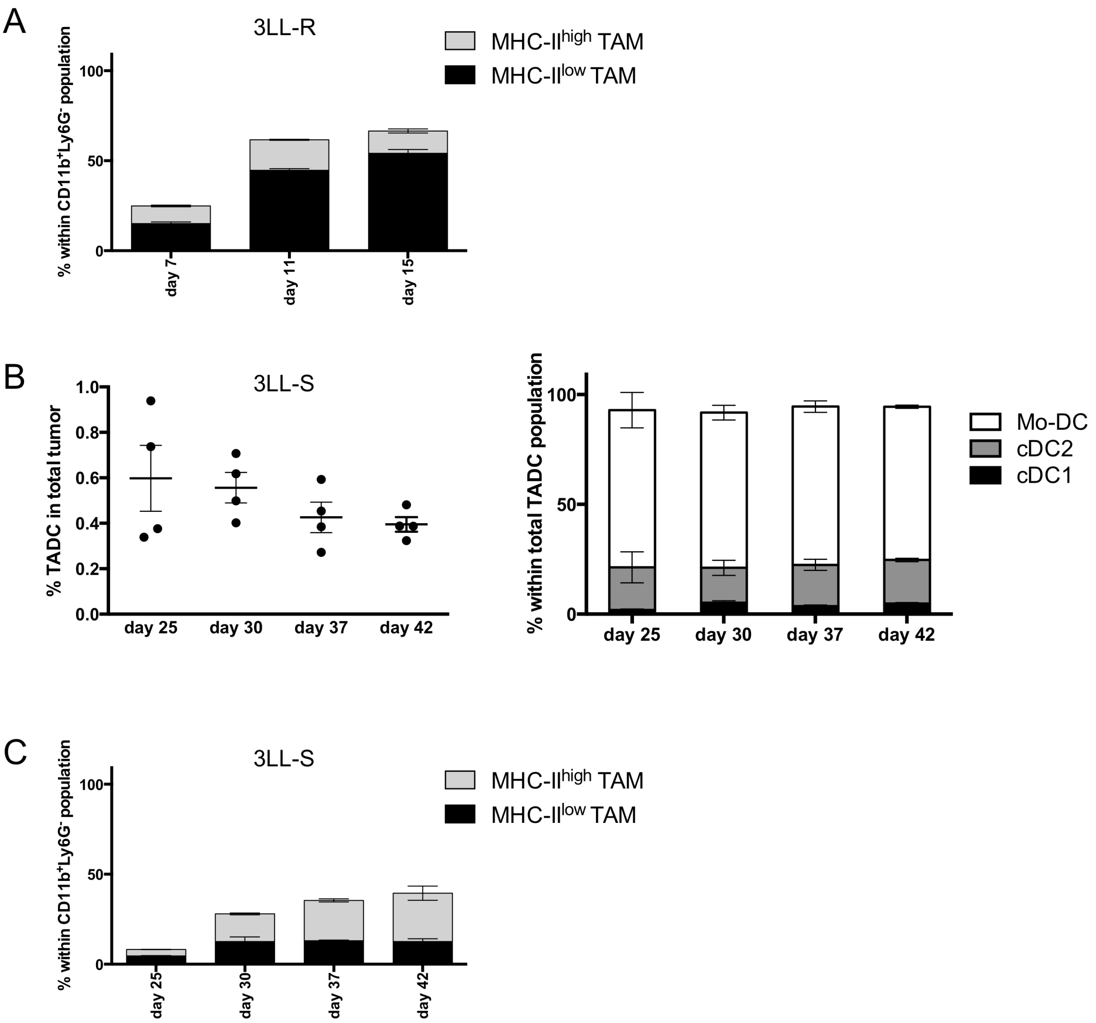

Supplementary figure 2, related to main figure 2. Several tumor types are infiltrated with distinct TADC subsets

(A) The amount of TAM subsets was assessed in single cell suspensions of 7, 11 and 15 day-old 3LL-R tumors. (B-C) The percentages of total TADC (B, left panel),TADC subsets (B, right panel) and TAM subsets (C) were assessed in single cell suspensions of 25, 30, 37, 42 day-old 3LL-S tumors. Graphs show mean ± SEM. Results are representative of 3 independent experiments with n≥4. Statistical analysis by one-way ANOVA. *, $p < 0.05$; **, $p < 0.01$; ***, $p < 0.001$; ****, $p < 0.0001$.

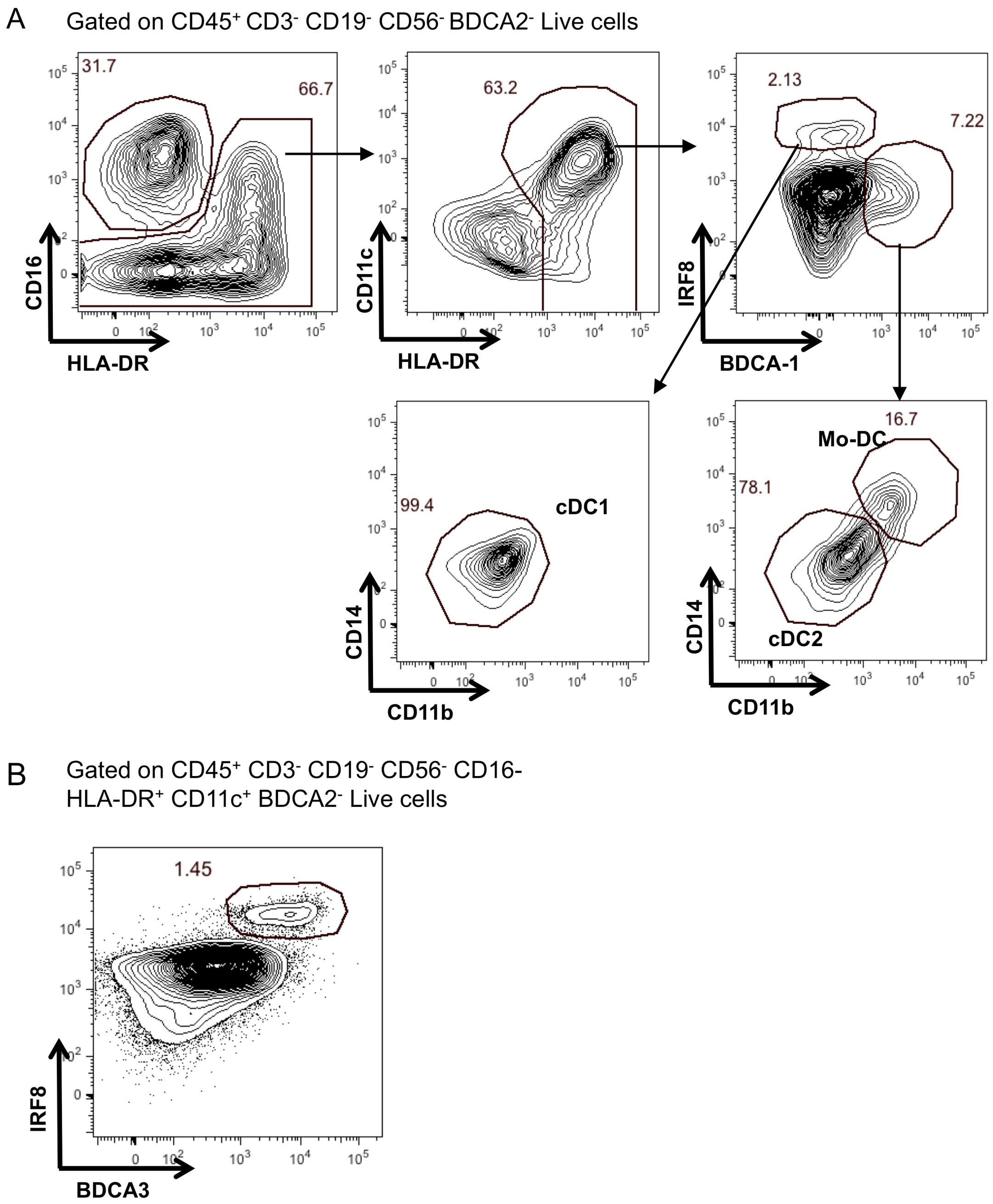

Supplementary figure 3, related to main figure 3. Gating strategy of TADC subsets in human colorectal and non-small cell lung carcinoma

(A) Human colorectal (CRC) tumor biopsies were pregated on CD45^{+} CD3^{-} CD19^{-} CD56^{-} BDCA2^{-} live cells and CD16^{-} CD11c^{high} HLA-DR^{+} cells were subdivided in (i) BDCA1^{-} IRF8^{+} CD14^{-} CD11b^{low} cDC1, (ii) BDCA1^{+} IRF8^{-} CD14^{-} CD11b^{+} cDC2 and (iii) BDCA1^{+} IRF8^{-} CD14^{+} CD11b^{high} Mo-DC. (B) Human non-small cell lung carcinoma (NSCLC) tumor biopsies were pre-

gated on $CD45^+$ $CD3^-$ $CD19^-$ $CD56^-$ $BDCA2^-$ $CD16^-$ $CD11c^{high}$ HLA-DR^+ live cells, containing a distinct $BDCA3^+$ $IRF8^+$ double positive cDC1 subset.

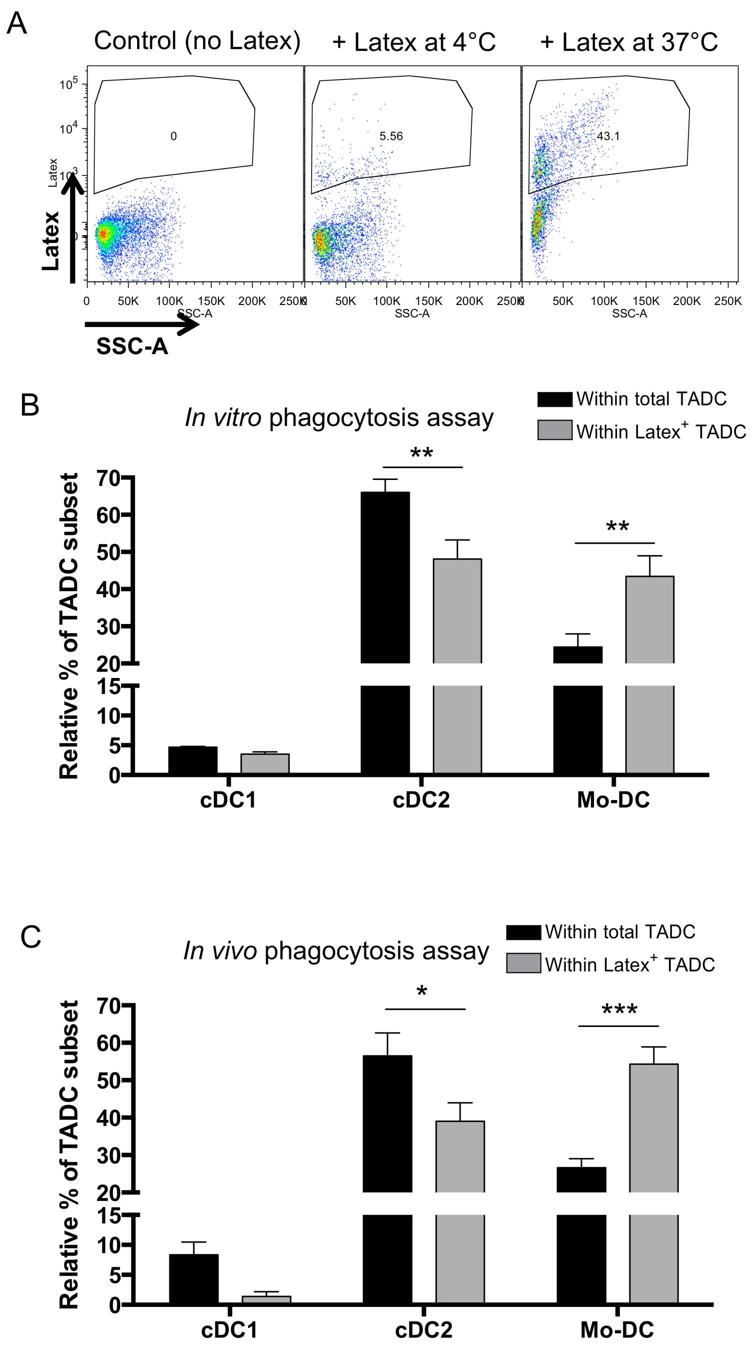

Supplementary figure 4, related to main figure 4. Antigen uptake and processing differ in the distinct TADC subsets

(A-B) *In vitro* phagocytosis assay. (A) Single cell suspensions of 12-day old 3LL-R tumors were cultured *in vitro*, in the absence (control) or presence of latex beads for 40 minutes at 4°C or 37°C. (B) The percentage of the distinct TADC subsets within the total TADC gate or within the Latex^{+} TADC gate are given. n=3 pools of 4 tumors. Statistical analysis by one-way ANOVA. **, $p < 0.01$. (C) *In vivo* phagocytosis assay. 12-day old 3LL-R tumor-bearing mice

were injected intravenously with latex beads 2 hours before sacrifice. The percentage of the distinct TADC subsets within the total TADC gate or within the Latex$^+$ TADC gate are given. n=3 pools of 4 tumors. Statistical analysis by one-way ANOVA. *, $p < 0.05$; ***, $p < 0.001$.

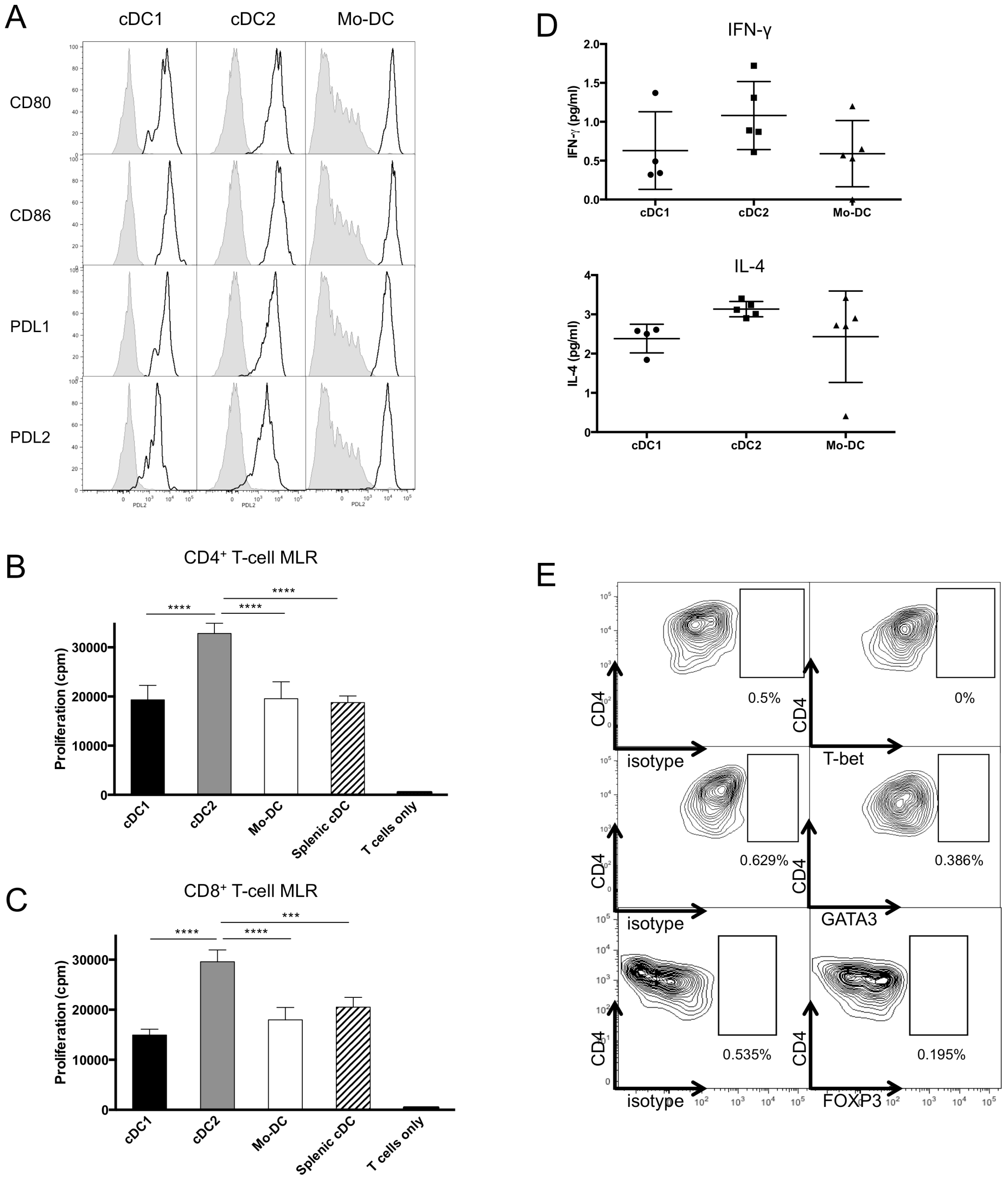

Supplementary figure 5, related to main figure 5. TADC subsets show distinct T-cell proliferative capacities

(A) Single cell suspensions of 12-day old 3LL-R tumors were stained for the indicated markers and histogram overlays are shown. Black line = expression of the indicated marker; shaded histogram = isotype control. Results are representative of 2 independent experiments with n≥4. (B-C) Antigen-presenting activities of TADC subsets from 3LL-R tumors in comparison with splenic $CD11c^{hi}MHC\text{-}II^{hi}B220^{-}Ly6C^{-}$ cDC from naive C57Bl/6 mice. Sorted TADC or splenic cDC were cultured in the presence of purified allogeneic BALB/c $CD4^{+}$ (B) or $CD8^{+}$ T cells (C) during 5 days and the proliferation of responding T cells was measured via ^{3}H-thymidine incorporation (cpm).

Results are representative of 2 independent experiments with n=3 pools of 10-12 tumors. Statistical analysis by one-way ANOVA. ***, $p < 0.001$; ****, $p < 0.0001$. (D) Supernatants of co-cultures of TADC subsets and OT-II T cells (DC/OT-II = 1/10) were tested for the presence of IFN-γ and IL-4 by luminex. n≥4. (E) Intracellular staining on OT-II T cells co-cultered with cDC2 for 3 days at a DC/OT-II ratio of 1/5 was performed for Th-inducing transcription factors T-bet, GATA3 and FOXP3. Isotype control and transcription factor staining are depicted.

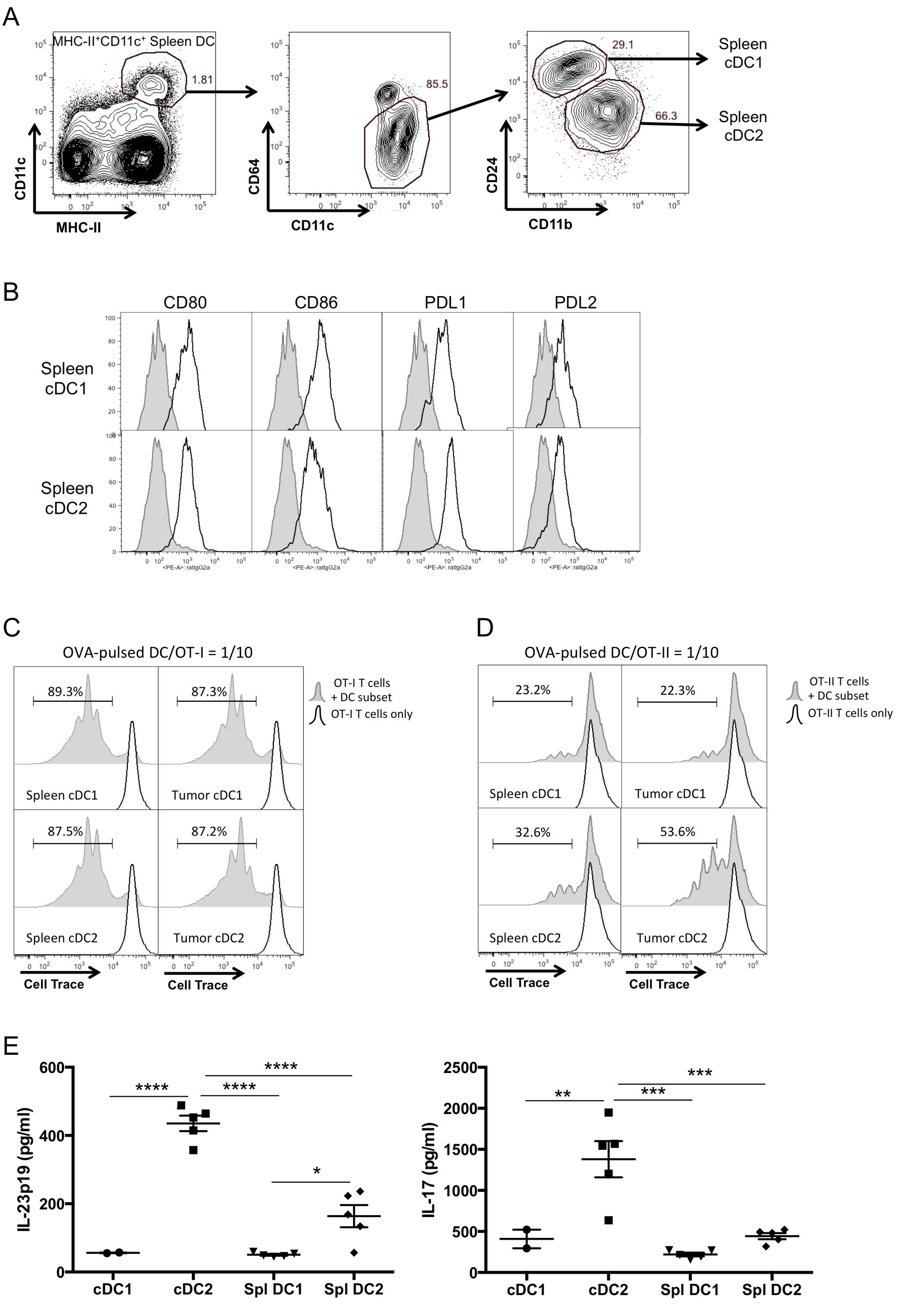

Supplementary figure 6, related to main figure 5. Tumor-derived cDC display equal to higher T-cell activating capacities as compared to splenic cDC

(A) Splenic single cell suspensions of naive mice were gated for CD64^{lo} MHC-II^{hi} CD11c^{hi} cells and subdivided in (i) CD24^{pos} CD11b^{lo} cDC1 and (ii) CD24^{low} CD11b^{pos} cDC2. (B) Single cell suspensions of naive spleens were stained for the indicated markers and histogram overlays are shown. Black line = expression of the indicated marker; shaded histogram = isotype control. n=4.
(C-D) Sorted TADC or splenic cDC subsets were co-cultured with OT-I (C) or OT-II (D) T cells for 3 days at a DC/T-cell ratio of 1/10 in the presence of OVA. The histograms represent CellTrace dilution, indicative for T-cell proliferation. Black line = non-stimulated T cells without DC; shaded histogram = T cells in the presence of DC. Results are representative of 2 independent experiments with n = pool of 12 tumors. (E) Supernatants of co-cultures of TADC or splenic DC subsets and OT-II T cells (DC/OT-II = 1/10) were tested for the presence of IL-23p19 and IL-17 by luminex. n=2 to 5 . Statistical analysis by one-way ANOVA. *, $p < 0.05$; **, $p < 0.01$; ***, $p < 0.001$; ****, $p < 0.0001$.

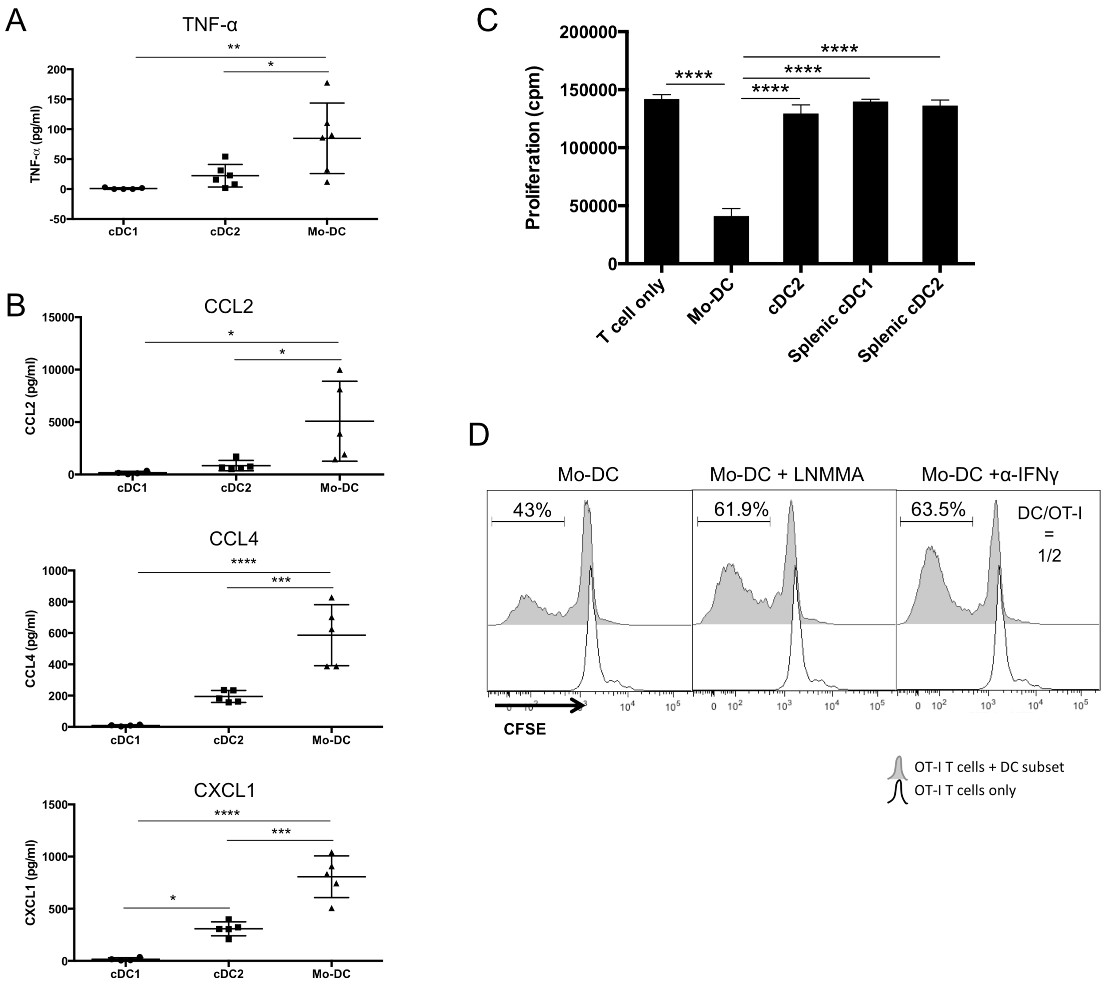

Supplementary figure 7, related to main figure 6. Mo-DC display an immune suppressive TIP-DC phenotype

(A-B) Supernatants of sorted TADC subsets cultured for 48h were tested for presence of (A) TNF-α, (B) CCL2, CCL4 and CXCL1 by luminex. n≥4. Statistical analysis by one-way ANOVA. *, $p < 0.05$; ** , $p < 0.01$, ***, $p < 0.001$; ****, $p < 0.0001$. (C) Mo-DC and cDC2 sorted from 12-day old 3LL-R tumor single cell suspensions and cDC1 and cDC2 sorted from naive spleens were added to anti-CD3/CD28-stimulated splenocytes at a DC/SPC ratio of 1/4 during 42h and the T-cell proliferation was measured via ^{3}H-thymidine incorporation (cpm). n = pool of 12 tumors. Statistical analysis by one-way ANOVA. ****, $p < 0.0001$. (D) Sorted Mo-DC were co-cultured with OT-I T cells for 3 days at a DC/OT-I ratio of 1/2. The histograms represent CFSE dilution, indicative for T-cell proliferation. Conditions compared are: non-stimulated T cells (No TADC) and T cells in the presence of Mo-DC with or without iNOS inhibitor (LNMMA) or α –IFN-γ. n=2.

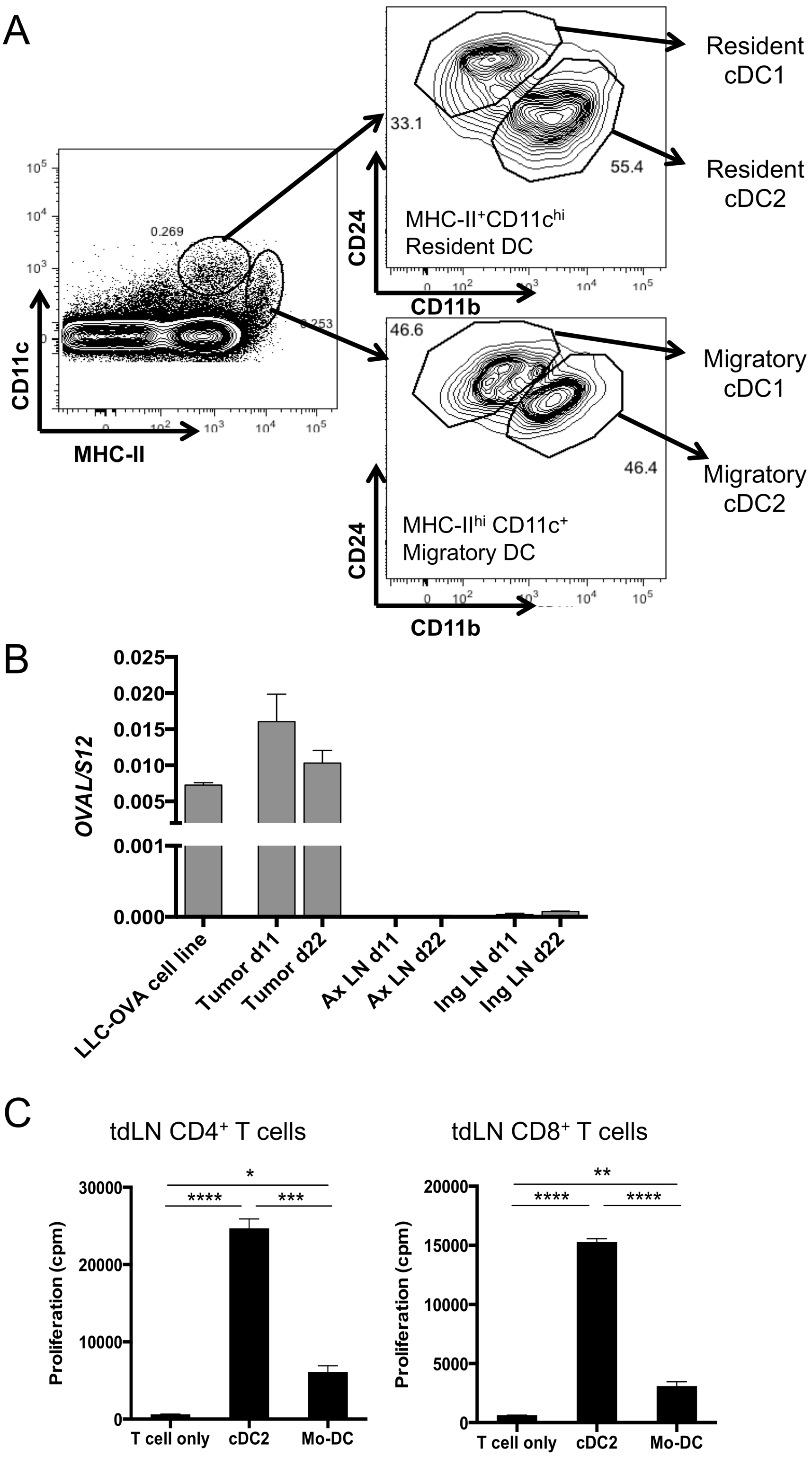

Supplementary figure 8, related to main figure 7. Both tumor-associated cDC subsets migrate to tumor draining lymph nodes and differentially activate CD8^{+} and CD4^{+} T cells

(A) Gating strategy of migratory and resident DCs in tumor draining lymph nodes. Single cell suspension of tumor-draining lymph nodes of 12-day old 3LL-R tumors were subdivided in (i) MHC-II^{pos} CD11c^{hi} CD24^{pos} CD11b^{lo} resident cDC1, (ii) MHC-II^{pos} CD11c^{hi} CD24^{low} CD11b^{pos} resident cDC2, (iii) MHC-II^{hi} CD11c^{pos} CD24^{pos} CD11b^{lo} migratory cDC1 and (iv) MHC-II^{hi} CD11c^{pos} CD24^{low} CD11b^{pos} migratory cDC2. (B) Expression of OVA was assessed in LLC-OVA cancer cells and tumor and tumor-draining lymph nodes (axillary or inguinal) of 11-day and 22-day old LLC-OVA tumor-bearing mice using qRT-PCR. The expression was normalized based on the S12 housekeeping gene. n=3. (C) CD4^{+} or CD8^{+} T cells of tdLN of 12-day old LLC-OVA bearing mice were co-cultured for 72h with TADC sorted from LLC-OVA tumors at a 1/2 TADC/T-cell ratio. T-cell proliferation was measured via ^{3}H-thymidine incorporation (cpm). n= pool of 12 tumors. Statistical analysis by one-way ANOVA. *, $p < 0.05$; **, $p < 0.01$; ***, $p < 0.001$; ****, $p < 0.0001$.

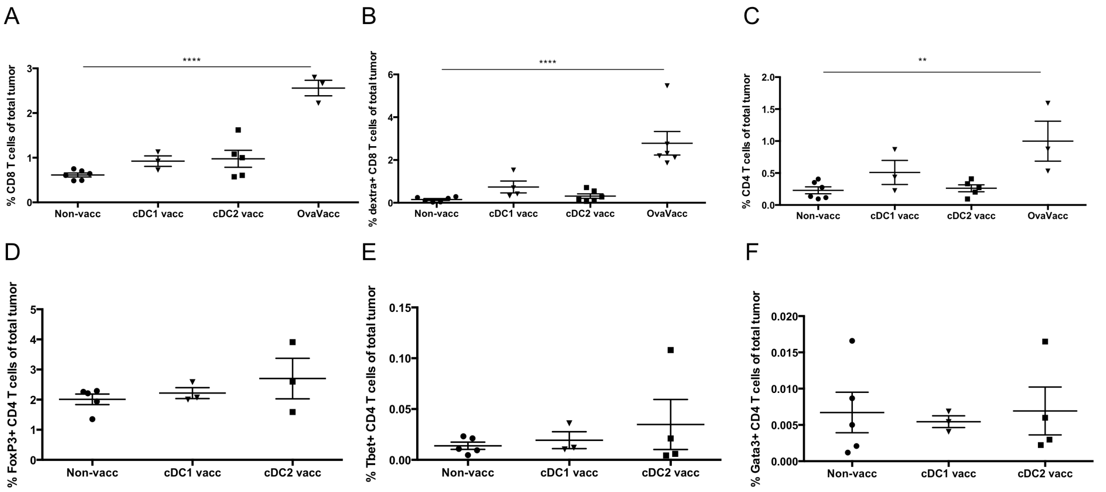

Supplementary figure 9, related to main figure 8. cDC2 vaccination is more beneficial than cDC1 vaccination in LLC-OVA tumor-bearing mice and repolarizes CD4$^+$ T cells to a Th17 phenotype

(A-F) Percentages of CD8$^+$ T cells (A), OVA-specific CD8$^+$ T cells (B), CD4$^+$ T cells (C), Foxp3$^+$ CD4$^+$ T cells (D), Tbet$^+$ CD4$^+$ T cells (E), Gata3$^+$ CD4$^+$ T cells (F), in LLC-OVA tumors after vaccination with LLC-OVA TADC subsets following the protocol depicted in (Figure 8A). For all experiments, results are representative of 2 independent experiments with n = 4-15 tumors. Statistical analysis by one-way ANOVA. *, $p < 0.05$; **, $p < 0.01$; ***, $p < 0.001$; ****, $p < 0.0001$.

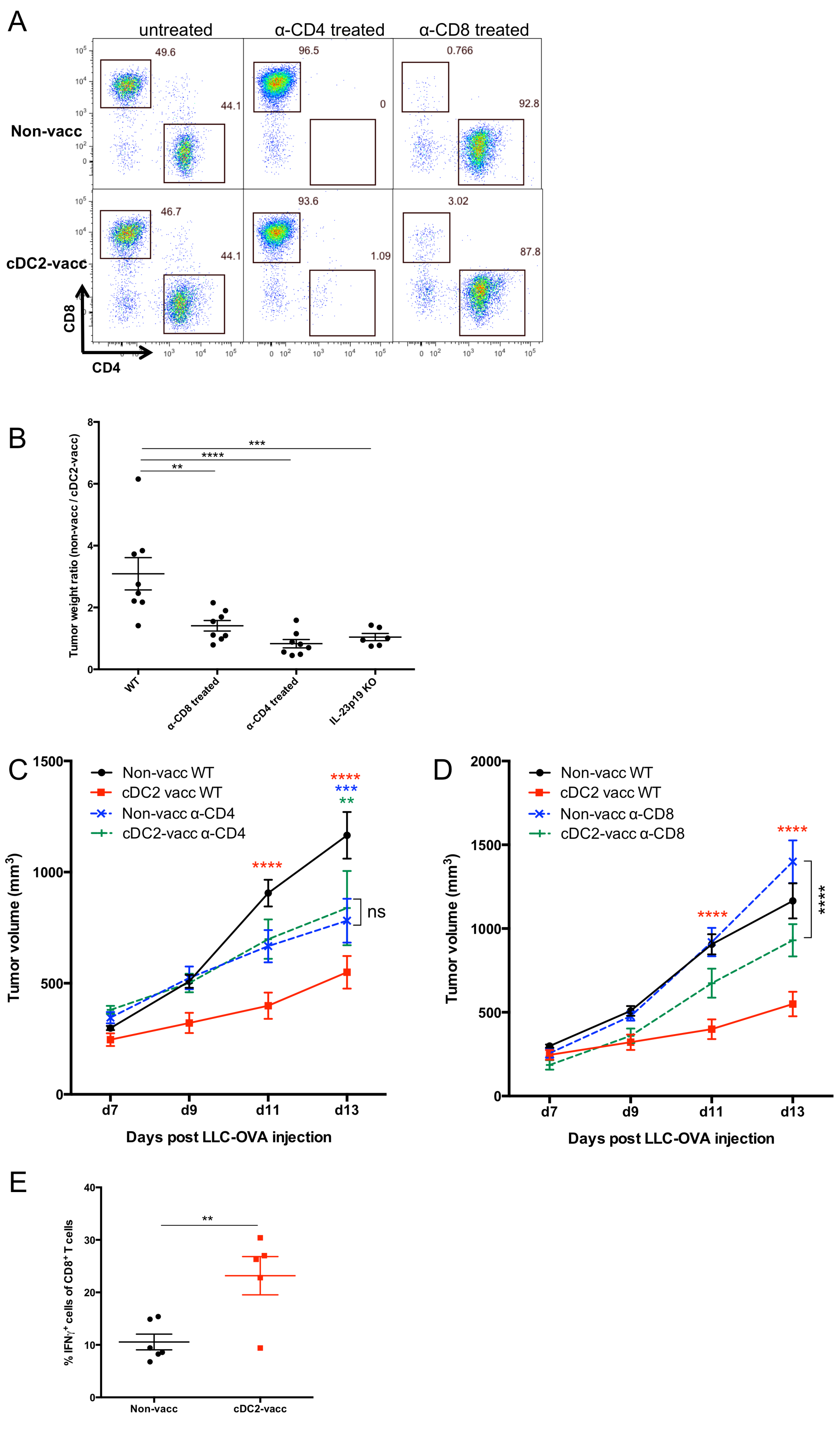
A
untreated
α-CD4 treated
α-CD8 treated
49.6
44.1
96.5
0
0.766
92.8
Non-vacc
46.7
44.1
93.6
1.09
3.02
87.8
cDC2-vacc
CD8
CD4
B
Tumor weight ratio (non-vacc / cDC2-vacc)
**
****
***
WT
α-CD8 treated
α-CD4 treated
IL-23p19 KO
C
Non-vacc WT
cDC2 vacc WT
Non-vacc α-CD4
cDC2-vacc α-CD4
ns
Tumor volume (mm3)
Days post LLC-OVA injection
d7
d9
d11
d13
D
Non-vacc WT
cDC2 vacc WT
Non-vacc α-CD8
cDC2-vacc α-CD8
Tumor volume (mm3)
Days post LLC-OVA injection
E
% IFNγ+ cells of CD8+ T cells
**
Non-vacc
cDC2-vacc

Supplementary figure 10, related to main figure 8. The anti-tumor effects of cDC2 vaccination depend mainly on the induction of Th17 cells

(A) Non-vaccinated or cDC2-vaccinated mice were treated every 2-3 days as from the day before tumor inoculation with anti-CD4 or anti-CD8 depleting Ab. Representative plots of blood of 13-day old LLC-OVA tumor-bearing mice gated on $CD45^+$ $Tcrb^+$ cells show the amount of $CD4^+$ and $CD8^+$ T cells. (B) Tumor weight ratio (non-vacc / cDC2-vacc) of LLC-OVA tumors after vaccination with LLC-OVA-derived cDC2 in WT, anti-CD8 treated, anti-CD4 treated and IL-23p19 KO mice. (C-D) Growth curve of LLC-OVA tumors after vaccination with LLC-OVA-derived cDC2 in untreated, anti-CD4 (C) or anti-CD8 (D) treated mice. (E) Percentages of $IFN\gamma^+$ $CD8^+$ T cells in LLC-OVA tumors after vaccination with LLC-OVA-derived cDC2 following the protocol depicted in (Figure 8A). $n \geq 6$. Statistical analysis by one-way ANOVA. *, $p < 0.05$; **, $p < 0.01$; ***, $p < 0.001$; ****, $p < 0.0001$.

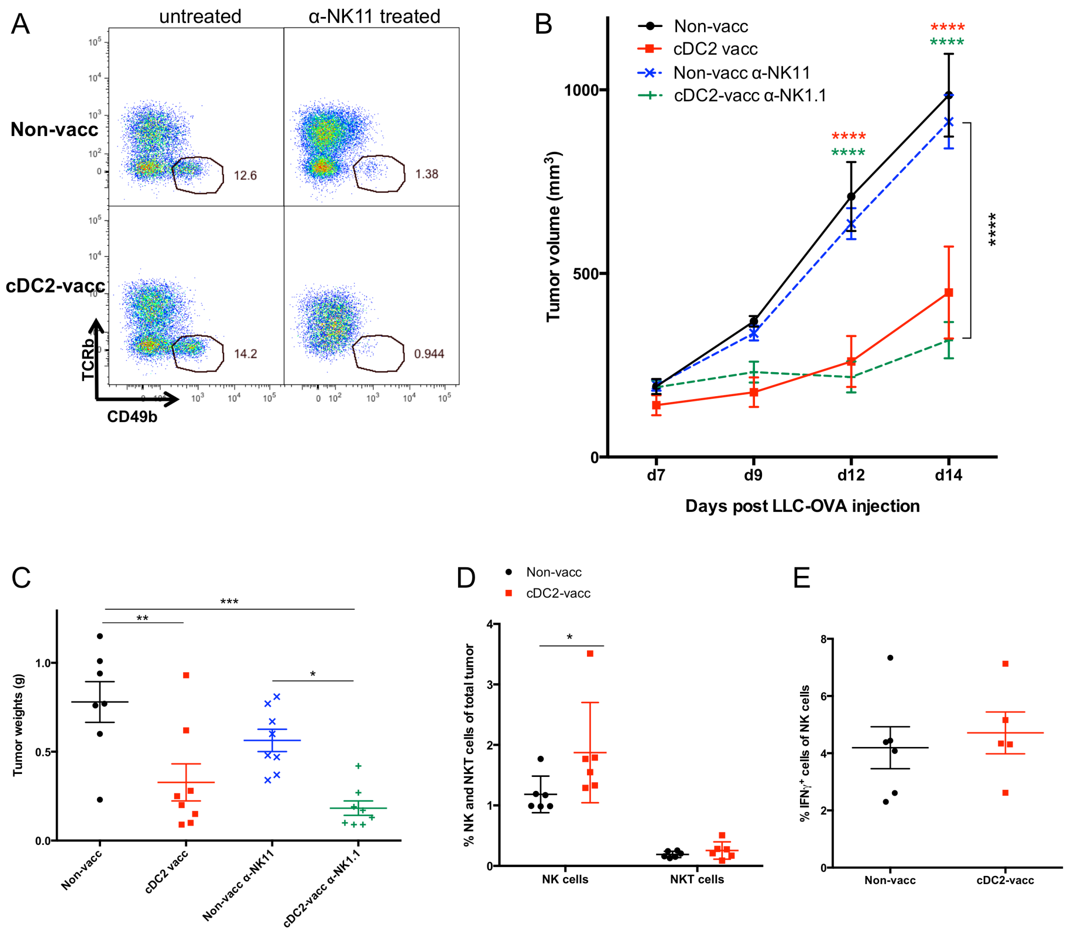

Supplementary figure 11, related to main figure 8. NK cells are not involved in the anti-tumor protection conferred by cDC2 vaccination

(A) Non-vaccinated or cDC2-vaccinated mice were treated every 2-3 days as from the day before tumor inoculation with anti-NK1.1 depleting Ab. Representative plots of 13-day old LLC-OVA tumors gated on CD45^{+} CD11b^{-} cells show the amount of NK cells. (B-C) Growth curve (B) and tumor weights (C) of LLC-OVA tumors after vaccination with LLC-OVA derived cDC2 in untreated or anti-NK1.1 treated mice. (D-E) Percentages of NK and NKT cells (D) and IFNγ^{+} NK cells (E) in LLC-OVA tumors after vaccination with LLC-OVA-derived cDC2 following the protocol depicted in Figure 8A. n ≥ 6. Statistical analysis by one-way ANOVA. *, $p < 0.05$; **, $p < 0.01$; ***, $p < 0.001$; ****, $p < 0.0001$.

SUPPLEMENTARY EXPERIMENTAL PROCEDURES

Supplementary table 1: Antibodies for flow cytometry (FACS)

Marker	Dilution	Clone	Source
7AAD	1:100	-	BD Biosciences
B220	1:600	Ra3-6B2	BD Biosciences
BDCA-1 Biotin	1:10	AD5-8E7	Miltenyi
BDCA-2 PE	1:50	AC144	Miltenyi
BDCA3-APC	1:10	AD5-14H12	Miltenyi
CCR2 PE	1:40	475301	R&D systems
CCR3 PE	1:40	83101	R&D systems
CCR7 Biotin	1:100	4B12	eBioscience
CD1d PE	1:600	1B1	BD Biosciences
CD3 FITC	1:50	UCHT1	eBioscience
CD4 PerCP-Cy5.5 / Pe-Cy7 / PE / APC	1:600	RM4-5 / GK1.5 / RM4-5 / RM4-5	BD Biosciences / eBioscience
CD8a PE / PE / APC	1:600	YTS169.4 / 16-10A1/ 53-6.7	Life technologies / eBioscience
CD11b BV605 / PerCP-Cy5.5 / AF488 / PE-Cy7	1:200 1:600	M1/70	BD Biosciences / eBioscience
CD11c PerCP-Cy5.5 / FITC / PE-Cy7 / PE-Cy7 (anti-human) / PE / APC	1:600 1:20	N418 / N418 / HL3 / 3.9 / HL3	BD Biosciences / eBioscience
CD14 PB	1:100	TuK4	Invitrogen
CD16 PE-TexasRed	1:100	3G8	Invitrogen
CD19 FITC / PE	1:20 / 1:600	HIB19 / 1D3	BD Biosciences
CD24 PE / PE-Cy7	1:600	M1/69	eBioscience
CD25 FITC	1:600	3C7	Biolegend
CD40 FITC	1:600	3/23	BD Biosciences
CD45 APC-Cy7 / BV421 /	1:600	30-F11 / 30-F11 /	Biolegend / BD
CD45.1 FITC	1:600	A20	eBioscience
CD45.2 AF700	1:600	104	eBioscience
CD49b APC	1:600	DX5	eBioscience
CD56 FITC	1:10	MEM188	eBioscience
CD64 PE / APC	1:600	Y54-5/7.1	Biolegend
CD80 PE	1:600	16-10A1	BD Biosciences
CD86 PE	1:600	Gl-1	BD Biosciences
CD103 biotin	1:600	M290	BD Biosciences

CD127 eFluor450	1:600	A7R34	eBioscience
CD172a PE / APC	1:600	P84	BD Biosciences/
DEC-205 PE	1:600	205yekta	eBioscience
Donkey anti-goat IgG AF647	1:1000	Polyclonal	Invitrogen
F4/80 PE / FITC / AF488	1:200	CI:A3-1	Biorad
FcεR1 Biotin	1:600	MAR-1	eBioscience
Fixable Live/Dead stain	1:1000	/	eBioscience
Foxp3 PerCP-Cy5.5	1:100	FJK-16s	eBioscience
Gata-3 AF647	1:100	TWAJ	eBioscience
Goat anti-rabbit	1:40	Polyclonal IgG	Life Technologies
Goat IgG control biotin	1:100	Polyclonal goat IgG	R&D systems
IA/IE BV421 / PerCP-Cy5.5 / APC-eFluor780	1:600	M5/114.15.2	BD Biosciences / Biolegend/
IFN-γ PE / APC	1:100	XMG1.2	BD Biosciences /
iso hamster IgG1, λ1 PE	1:600	G235-2356	BD Biosciences
Iso mouse IgG1 k eFluor660	1:100	eP3.6.2.8.1	eBioscience
Iso Rat IgG1 k PE / APC	1:600	R3-34 / eBRG1	BD Biosciences/
iso rat IgG2a Biotin / FITC / PE / APC	1:600	eBR2a / R35-95 / R35-95 / eBR2a	BD Biosciences / eBioscience
iso rat IgG2b, κ, PE / AF647	1:600	A95-1 / eB149/10H5	BD Biosciences /
IRF4 unlabeled	1:400	M-17	Santa Cruz
IRF8 PerCP-Cy5.5	1:400	V3GYWCH	eBioscience
HLA-DR APC-Cy7	1:100	LN3	eBioscience
Ly6C AF488 / AF647	1:600	ER-MP20	Serotec
Ly6G FITC / PE	1:600	1A8	BD Biosciences
MerTK Biotin	1:100	Polyclonal goat IgG	R&D systems
MitoSOX™ Red (MitO2)	1:2000	/	Life Technologies
NK1.1 FITC/PE	1:600	PK136	eBioscience
NOS2 unlabeled	1:40	M17	Santa Cruz
PD-L1 (B7-H1) PE	1:600	MIH5	BD Biosciences
PD-L2 (B7-DC) PE	1:600	TY25	eBioscience
Rabbit IgG unlabeled	1:400	Polyclonal rabbit IgG	Santa Cruz
RORγt APC	1:100	AFKJS-9	eBioscience
SiglecF PE	1:600	E50-2440	BD Biosciences
SIINFEKL Biotin	1:100	eBio25-D1.16	eBioscience
SIINFEKL class I MHC dextramer APC	1:5	/	Immudex
Streptavidin BV786/ PE	1:200	Streptavidin	BD Biosciences
T-bet eFluor660	1:100	eBio4B10	eBioscience
TCRb BV421 / FITC	1:600	H57-597	BD Biosciences /
TNF-α PE	1:100	MP6-XT22	eBioscience

XCR1 PE	1:600	ZET	Biolegend

Supplementary table 2: Primer sequences for qRT-PCR.

Gene	Forward Primer	Reverse Primer
Arg1	TCACCTGAGCTTTGATGTCG	TTATGGTTACCCTCCCGTTG
Il1b	GTGTGGATCCAAAGCAATAC	GTCTGCTCATTCATGACAAG
Il4ra	GCAGATGGCTCATGTCTGAA	CTCTGGGAAGCTGGGTGTAG
Il12b	TCAGGGACATCATCAAACCA	CTACGAGGAACGCACCTTTC
MMP9	TGAATCAGCTGGCTTTTGTG	GTGGATAGCTCGGTGGTGTT
Mrc1 (MMR)	GCAAATGGAGCCGTCTGTGC	CTCGTGGATCTCCGTGACAC
Nos2 (iNOS)	GCTTCTGGTCGATGTCATGAG	TCCACCAGGAGATGTTGAAC
OVAL (of Gallus gallus)	CGTGGATTCTCAAACTGCAA	CACCAACATGCTCATTGTCC
Ptgs2 (COX2)	CAGGCTGAACTTCGAAACAG	CAGCTACGAAAACCCAATCA
Stab1	ACGGGAAACTGCTTGATGTC	ACTCAGCGTCATGTTGTCCA
Tnf	CCTTCACAGAGCAATGACTC	GTCTACTCCCAGGTTCTCTTC
