# Supplementary material for: The tumour microenvironment harbours ontogenically distinct dendritic cell populations with opposing effects on tumour immunity
Source: Nat Commun. 2016 Dec 23;7:13720. doi: 10.1038/ncomms13720 (PMC5196231; doi:10.1038/ncomms13720)
Supplement: Peer Review [file ncomms13720-s2.pdf]

PEER REVIEW FILE

Reviewers' comments:

Reviewer #1 (Remarks to the Author):

Laoui and colleagues present in this manuscript a comprehensive analysis of dendritic cells and monocyte derived APC within tumor microenvironments. Using a large number of surface markers they phenotype these cells with a high degree of precision is a variety of both mouse and human tumors. They found a fair degree of heterogeneity and that monocyte derived DC efficiently acquire antigen but have poor T cell priming capacity apparently due to their production of NO. They go on to show that DC subsets in tumors efficiency migrate and can prime CTL (cDC1) or Th17 (cDC2) responses. Finally, both cDC1 and cDC2 can partially vaccinate mice against a subsequent tumor challenge with cDC2 promoting a reduction in MDSC and cDC1 promoting expansion of anti-tumor CTL. Overall, this manuscript contains many nicely executed experiments. It is well written. DC phenotyping can be treacherous waters, especially in non homeostatic conditions, but the authors have done a nice job here. The phenotyping of DC from a variety of tumor types is also an important contribution.

My concerns are as follows:

1. Priming of naïve cells to develop an anti-tumor response likely occurs prior to the development of observable tumors. Thus, the experiments demonstrating that cDC from tumors can prime an anti-tumor immune response would seem less relevant than their ability to expand/activate established effectors that are recruited into tumors. In addition, I am also concerned that though the vaccination data does show the function of cDC subsets, the effect was fairly minor.
2. The significance of Th17 differentiation in the context of anti-tumor immune responses is unclear. I believe the effort was to completely phenotype DC function in this context, but the biological relevance is unclear.
3. The numbers of tumor DC in Figure 1c in mice with CCR2-/- Flt3L CM-CSFR-/- is very clever but I would have anticipated a much larger effect. Are these DC subsets in non-tumor tissues affected to a greater extent?
4. The flow plots in Figure 1A shows a larger number of MHC-II-mid CD11c+ cells that are absent in the plot in Figure 1B. Why were these cells not gated in figure 1A and where did they go in Figure 1B?
5. In Figure 3, why was CD141 (BCDA3) not included in the analysis? IRF8 may be a substitute, but CD141 is more commonly found in the literature.
6. In Figure 6, can the addition of moDC inhibit the response?

7. In figure 8, were DC injected at the same site as tumor inoculation? Have resident memory T cells been created?

Reviewer #2 (Remarks to the Author):

"The tumor microenvironment harbors ontogenically distinct dendritic cell populations with opposing effects on tumor immunity" by Damya Laoui et al.

In this manuscript, using murine and human tumor models, the authors demonstrated that tumors harbored ontogenically discrete TADC subpopulations. Monocyte-derived TADC were prominent in TA processing, but were not strong T-cell stimulators due to NO-mediated immunosuppression. Pre-cDC-derived TADC displayed the ability to migrate to the lymph nodes, while cDC1 activated CD8 T cells and cDC2 induced Th17 cells. Mice vaccinated with cDC2 demonstrated an inhibited tumor growth with a reprogramming of pro-tumor TAM and a reduction of MDSC. Vaccination with cDC1 induced antitumor CTL. The authors concluded targeting specific TADC subsets or their precursors might be important for improved therapeutic interventions.

The authors have used interesting and elegant experiments to determine TADC origin in mice. For instance, utilization of CCR2-deficient mice is a great idea and confirmed the existence of ontogenically different TADS populations. In line with this, utilization of Flt3L-KO and GM-CSFR-KO mice is also a strength of the manuscript. As a result, the authors unraveled the coexistence of ontogenically and functionally distinct TADC subsets and determined their effect on T cells in vitro and the effect on tumor growth in vivo. In vivo analysis of DC vaccines in different tumor models is a magnificent part of the manuscript as it additionally adds the involvement of TAM and MDSC. This part can be an individual paper. The minor concern is that the use of term "therapeutic effect" (or similar) is misleading. The authors did not use the 'therapeutic' tumor models, but utilized 'prophylactic' tumor models where DC were injected prior to tumor cell administration. Thus, only protective effect of DC vaccines was determined, not a therapeutic effect. It will be helpful if the authors improve this part of Results and correct Discussion accordantly. It seems somehow inappropriate to discuss therapeutic DC vaccines without presenting any related data in the manuscript.

Overall, the results are very interesting, innovative and may have clinical significance. There are a lot of results and are very well presented, although all figures look quite busy. The only question is about immunosuppressive/tolerogenic TADC which were described in numerous publications. The authors did not reveal any DC subsets with strong immunosuppressive properties: NO blockage experiments are not related since immunosuppression was not revealed

(no experiments analyzed the effect of DC on pre-activated T cells). Non-functional or functionally deficient TADC have been repeatedly described, but have not been seen in these studies. All TADC subsets were functionally active, although affect different targets. It will be very helpful if this important issue is explained in the manuscript.

Inclusion of human tumor specimens and human TADC isolation is an interesting part of the manuscript, but this direction has not been developed and one can suggest omitting this part from the manuscript. This is just a suggestion.

Minor comments:
- Description of DC ontogenesis in Introduction does not, even briefly, explain differences between murine and human DC subsets.
- The overall characterization of TADC is based on the not-widely-accepted classification of macrophages, which does not utilize CD11c expression. In addition, it has been recently reported that tolerogenic TADC may differentiate from MDSC and therefore express Gr1. There is not doubts that different classifications of DC and TADC exists, and it will be helpful to justify selection of either one.
- It is not quite correct to conclude about the antigen uptake ability of DC utilizing a single phagocytosis assay, since different DC subsets display differential ability for phagocytosis, pinocytosis and receptor-mediated endocytosis (all of these may be involved in Ag uptake). One can suggest a careful re-phrasing of this part of Results. Of note: this issue is of importance since in the next part of Results, the authors used DQ-ovalbumin, which is taken up by pinocytosis and has nothing to do with bead phagocytosis.
- What was the level of DQ-ovalbumin uptake by different TADC subsets? May be this can answer the previous concern? Can the differences in DQ-ovalbumin processing be explained by the differences in DQ-ovalbumin uptake?
- To suggest that any TADC subset can inhibit T cell proliferation, may be the direct way is to mix TADC with pre-activated proliferating T cells.
- It is quite hard to understand the activating/inhibitory activity of TADC subsets without comparing the results with a "classic gold standard" - bone marrow-derived DC. Is this possible?
- Did the authors used soluble "1 μg/ml anti-CD3 and 2 μg/ml CD28" Abs for T cell stimulation? Did it work? Isn't it important to use bead- or surface-coated Abs?
-Introduction of TADC might be slightly extended to introduce different TADC subsets and function (just a suggestion).

Reviewer #3 (Remarks to the Author):

The first part of this manuscript contains a detailed phenotypic and precursor analysis of 3 different subpopulations of tumor-associated Dendritic Cells, demonstrated to be present both in different mouse tumor models and in human lung and colorectal cancers. The analysis appears as carefully performed, and the fact that these 3 distinct subsets can be defined at various frequencies in several different tumor types is of interest particularly to those in the DC field. But the biological implications of this finding, particularly when it comes to therapeutic effects for tumor vaccination, is less convincing, as commented on below.

Many of the observations, although of considerable interest, are quite superficial and would need a more thorough analysis in order to have a more profound impact on our knowledge of DC subsets. For example, to what extent does the variation of the different subsets shown in Fig 1C depend on their different ability to migrate to the tumor, as compared to their capacity to survive there ?

The tumor protection data after vaccination with the different DC subsets as shown in Fig 8 B and Fig 10 shows that the protection is not very impressive, particularly when compared to the effect of vaccination with Ova. Also they must analyze this tumor protection more in detail, e.g. if this is a CD8 T cell mediated effect, by performing depletion of CD8 cells in vivo. DC cells are known to have effects on NK cells and it cannot be excluded that the observed effects are NK mediated.

Also the discordance they notice between the in vivo protection data, where the cDC2 subset is superior to the cDC1 subset, and the multimer staining for CTLs where cDC1 subset induced higher %, also requires a more thorough analysis, e.g. studying the effect on the innate immune rejection mechanisms such as NK cells.

Minor comment;
The colors and shapes of the symbols in Fig 8 B are mixed up.

Response to all reviewers

We thank the reviewers for their evaluation of our manuscript and advice. While we appreciated the overall positive assessment of our manuscript, we have taken the outstanding criticisms seriously and addressed them with a substantial amount of new experiments. We hope that the reviewers will concur with us that the new data and revisions have improved the paper and strengthened its conclusions. To highlight several new very relevant findings and to make the figures less busy, we moved some figure panels to supplemental figures. To make the reviewing process easier, all replies and changes in the manuscript are written in blue.

Point-by-point replies to the reviewers' comments:

Reviewer #1:

Laoui and colleagues present in this manuscript a comprehensive analysis of dendritic cells and monocyte derived APC within tumor microenvironments. Using a large number of surface markers they phenotype these cells with a high degree of precision is a variety of both mouse and human tumors. They found a fair degree of heterogeneity and that monocyte derived DC efficiently acquire antigen but have poor T cell priming capacity apparently due to their production of NO. They go on to show that DC subsets in tumors efficiency migrate and can prime CTL (cDC1) or Th17 (cDC2) responses. Finally, both cDC1 and cDC2 can partially vaccinate mice against a subsequent tumor challenge with cDC2 promoting a reduction in MDSC and cDC1 promoting expansion of anti-tumor CTL. Overall, this manuscript contains many nicely executed experiments. It is well written. DC phenotyping can be treacherous waters, especially in non homeostatic conditions, but the authors have done a nice job here. The phenotyping of DC from a variety of tumor types is also an important contribution.

My concerns are as follows:

1. Priming of naïve cells to develop an anti-tumor response likely occurs prior to the development of observable tumors. Thus, the experiments demonstrating that cDC from tumors can prime an anti-tumor immune response would seem less relevant than their ability to expand/activate established effectors that are recruited into tumors. In addition, I am also concerned that though the vaccination data does show the function of cDC subsets, the effect was fairly minor.

Reply: The reviewer correctly points out that the prime function of TADC residing in the tumor may be to expand or activate established effectors that are recruited to the tumor site. However, since cDC1 and cDC2 migrate to the tumor draining LN (tdLN) (Figure 7 in the manuscript), we believe that their capacity to stimulate naive T cells may also be of considerable importance.
Nevertheless, to assess whether TADC could re-activate *in vivo* primed T cells, we first aimed to co-culture TADC and $CD4^+$ or $CD8^+$ tumor-infiltrating lymphocytes (TIL) sorted from LLC-OVA tumors. It turned out to be technically unfeasible to purify

tumor-derived cDC1, CD4$^+$ or CD8$^+$ TIL in sufficient quantities and with acceptable purities to perform the experiment.

Next, we sorted TADC from LLC-OVA tumors and co-cultured them with CD4$^+$ or CD8$^+$ T cells purified from the tdLN of 12-day old LLC-OVA bearing mice. In these tdLN, the presence of antigen-experienced T cells can be anticipated. Interestingly, freshly isolated cDC2 and Mo-DC (insufficient cDC1 could be purified for this experiment) were able to restimulate *in vivo* primed tdLN-derived CD4$^+$ or CD8$^+$ T cells, whereby cDC2 were significantly better in triggering T-cell proliferation than Mo-DC (Figures R1A and R1B, new figure S8C in the manuscript). To confirm the T-cell restimulating capacity of cDC2 in an alternative setting, we co-cultured freshly isolated cDC2 with purified CD4$^+$ or CD8$^+$ T cells from the spleen of mice that were vaccinated with OVA in Complete Freund's Adjuvant (CFA). Again, cDC2 were shown to restimulate these *in vivo* primed T cells (Figures R1C and R1D, for the attention of the reviewer only).

Together, these data illustrate that at least tumor-associated cDC2 have the capacity to re-activate antigen-primed CD4$^+$ and CD8$^+$ T cells. Mo-DC are much less efficient in doing this, in line with their immunosuppressive properties.

Concerning the efficacy of the TADC vaccination, it should be stressed that the immunizations were performed with very low amounts of TADC (10^4 cells), which have not been manipulated in vitro (for example, no additional uploading with tumor antigen, no addition of cytokines or maturation signals). We believe our current data provide solid evidence that tumor-associated cDC1 and cDC2 are immunogenic, providing a base for further optimization of the protocol (dose, regimen of administration, route of administration) to maximize anti-tumor effects.

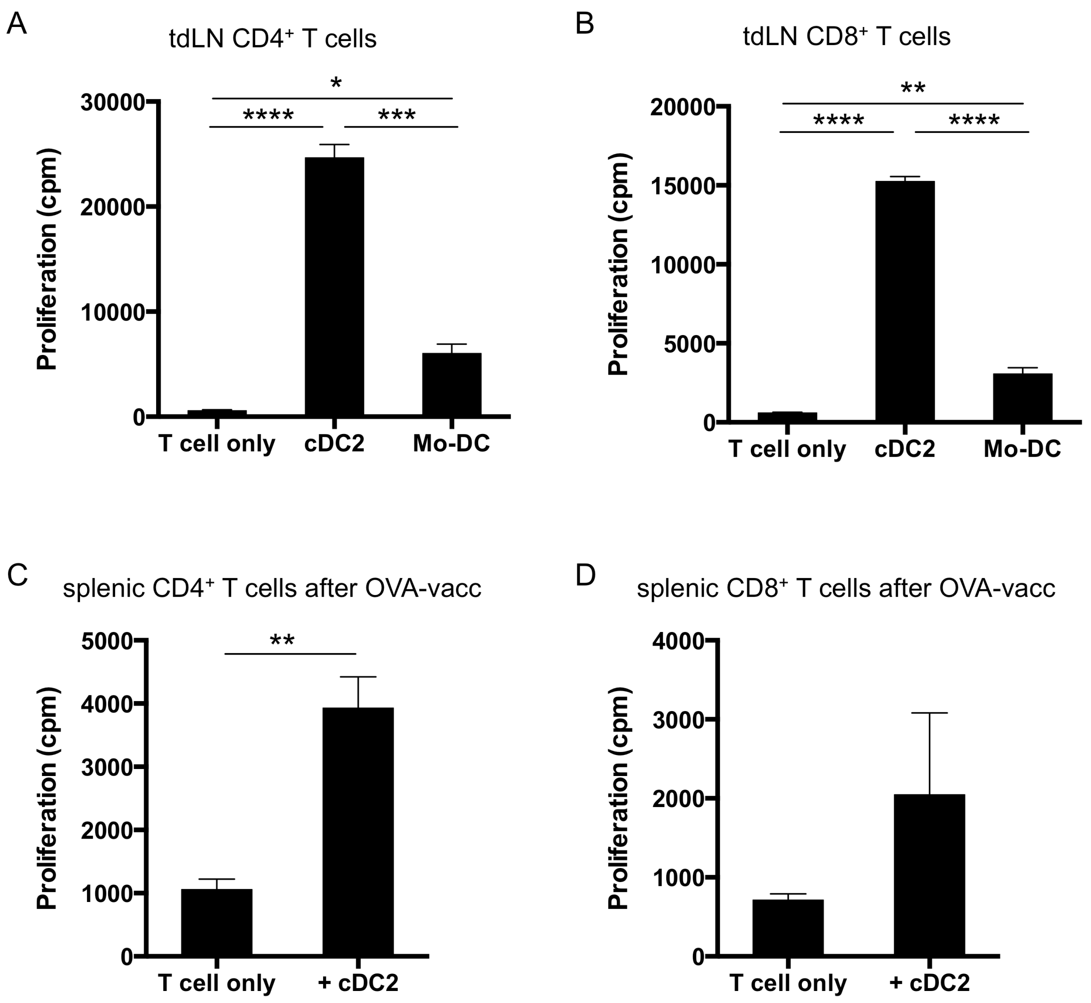

Figure R1. Tumor-derived cDC2 can reactivate *in vivo* primed T cells.
(A-B) $CD4^+$ (A) or $CD8^+$ (B) T cells of tdLN of 12-day old LLC-OVA bearing mice were co-cultured for 72h with TADC sorted from LLC-OVA tumors at a 1/2 TADC/T cell ratio. (C-D) Mice were vaccinate with OVA+CFA as depicted in Figure 8A in the manuscript. Splenic $CD4^+$ (C) or $CD8^+$ (D) T cells of 12-day old LLC-OVA bearing vaccinated mice were co-cultured for 72h with cDC2 sorted from LLC-OVA tumors at a 1/2 cDC2/SPC ratio. For all experiments, the proliferation of T cells was measured via ^{3}H-thymidine incorporation (cpm). n= pool of 12 tumors. Statistical analysis by one-way ANOVA. *, $p < 0.05$; **, $p < 0.01$; ***, $p < 0.001$; ****, $p < 0.0001$.

2. The significance of Th17 differentiation in the context of anti-tumor immune responses is unclear. I believe the effort was to completely phenotype DC function in this context, but the biological relevance is unclear.

Reply: By performing additional experiments, we provided evidence that $CD4^+$ T cells, and in particular Th17 cells, are needed for the anti-tumor effect of cDC2 vaccination.

1) First, antibody-mediated depletion of $CD4^+$ T cells (Figure R2A, new figure S10A in the manuscript) was shown to revert the retarded tumor growth upon cDC2

vaccination, suggesting that CD4$^+$ T cells are protective in this setting (Figure R2B, new figure S10C in the manuscript. This is remarkable, since CD4$^+$ T-cell depletion lowers tumor growth in non-vaccinated mice, indicating that cDC2 vaccination switches the CD4$^+$ T-cell pool from mainly pro-tumoral to mainly anti-tumoral.

2) To directly assess the significance of Th17 induction in retarding tumor growth upon cDC2 vaccination, we compared the vaccination effect in WT versus IL-23p19-deficient mice. IL-23 is a crucial cytokine for the induction of Th17 cells, so IL-23p19-deficient mice lack Th17 responses[1]. Without cDC2 vaccination, tumors grow equally well in WT and IL-23p19-deficient mice (Figure R2C, new figure 8E in the manuscript). However, upon cDC2 vaccination, tumors only grow slower in WT mice, while the vaccination protocol is totally ineffective in an IL-23p19-deficient background. These data indicate that Th17 cells play a non-redundant role in the anti-tumor protection conferred by cDC2 vaccination.

These new data are incorporated in the Results section (p15) and shown in Figure S10 and Figure 8E.

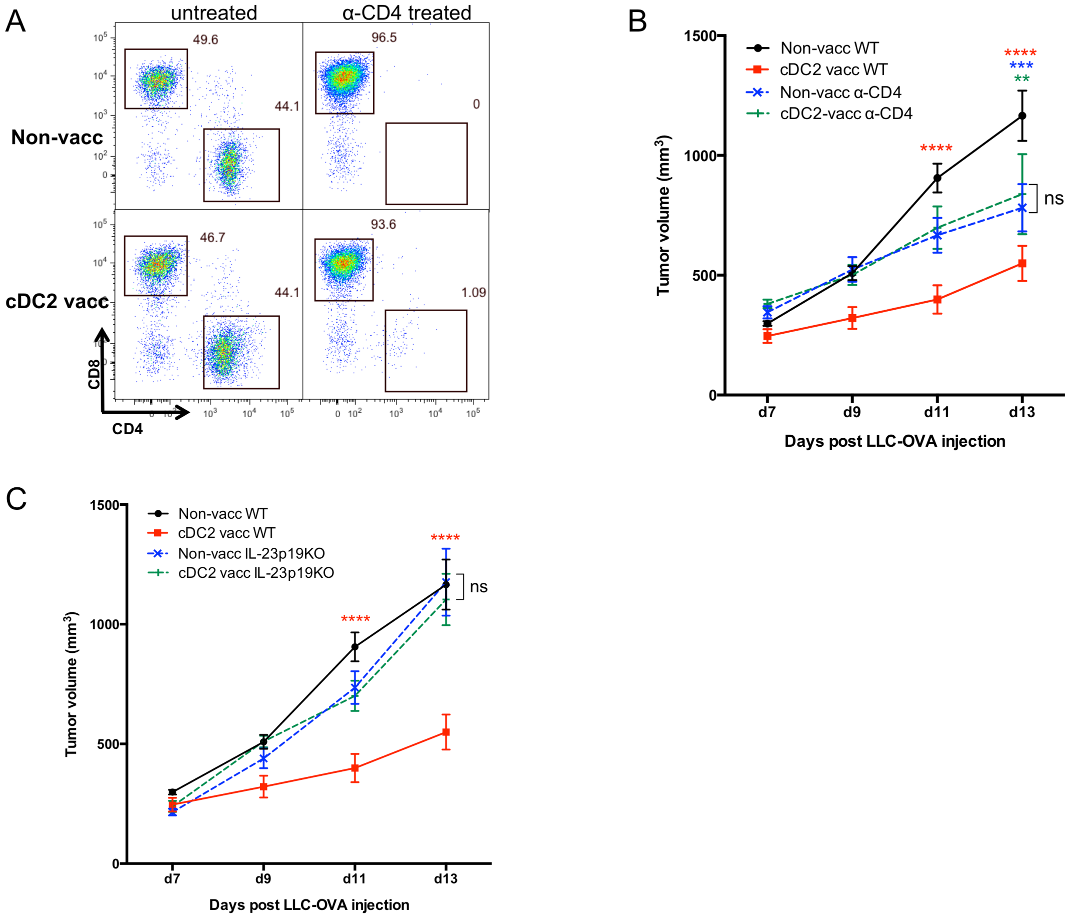

[1] Ghilardi N, Kljavin N, Chen Q, Lucas S, Gurney AL, de Sauvage FJ. Compromised Humoral and Delayed-Type Hypersensitivity Responses in IL-23-Deficient Mice. *The Journal of Immunology* 2004, **172**(5): 2827-2833.

Figure R2: The anti-tumor effects of cDC2 vaccination depend mainly on the induction of Th17 cells.
(A) Non-vaccinated or cDC2-vaccinated mice were treated every 3 days as from tumor inoculation with anti-CD4 or anti-CD8 depleting Ab. Representative plots of blood of 13-day old LLC-OVA tumor-bearing mice gated on $CD45^+$ $Tcrb^+$ cells show the amount of CD4 and CD8 T cells. (B) Growth curve of LLC-Ova tumors after vaccination with LLC-OVA derived cDC2 in untreated or anti-CD4 treated mice. (C) Growth curve of LLC-Ova tumors after vaccination with LLC-OVA derived cDC2 in WT or IL-23pKO mice. $n \geq 6$. Statistical analysis by one-way ANOVA. *, $p < 0.05$; **, $p < 0.01$; ***, $p < 0.001$; ****, $p < 0.0001$.

3. The numbers of tumor DC in Figure 1c in mice with CCR2-/- Flt3L CM-CSFR-/- is very clever but I would have anticipated a much larger effect. Are these DC subsets in non-tumor tissues affected to a greater extent?

Reply: We believe that the effect of CCR2-deficiency is clear-cut, with no alterations in the presence of tumor-associated cDC1 nor cDC2, but a nearly complete absence of monocyte-derived DC. The implication of Flt3L- and GM-CSFR-deficiency on the presence of cDC populations in tumors is indeed subtler. This is fully in line with existing, published data in non-tumor tissues, as properly discussed in our manuscript. Greter *et al.*[2] (reference 17 in our manuscript) already indicated that $CD103^+$ DC (cDC1) in non-lymphoid tissues are more dependent on GM-CSFR signaling than $CD11b^+$ DC (cDC2) under homeostatic conditions. Moreover, Kingston *et al.*[3] (reference 29 in our manuscript) reported a greater deficiency in non-lymphoid tissue cDC in Flt3L/GM-CSFR-double deficient animals as compared to Flt3L-single deficient animals. These published data suggest an intricate interplay between Flt3L and GM-CSF for the generation and maintenance of non-tumor cDC. Since these data are available in literature, we prefer to highlight them in the Discussion (as we already did, p18-19), rather than performing additional experiments.

4. The flow plots in Figure 1A shows a larger number of MHC-II-mid CD11c+ cells that are absent in the plot in Figure 1B. Why were these cells not gated in figure 1A and where did they go in Figure 1B?

Reply: The $MHC\text{-}II^{mid}$ $CD11c^+$ population in Figure 1A corresponds to the tumor-associated macrophages, which have previously been described by us [4, 5]

[2] Greter M, Helft J, Chow A, Hashimoto D, Mortha A, Agudo-Cantero J, *et al.* GM-CSF controls nonlymphoid tissue dendritic cell homeostasis but is dispensable for the differentiation of inflammatory dendritic cells. *Immunity* 2012, **36**(6): 1031-1046.

[3] Kingston D, Schmid MA, Onai N, Obata-Onai A, Baumjohann D, Manz MG. The concerted action of GM-CSF and Flt3-ligand on in vivo dendritic cell homeostasis. *Blood* 2009, **114**(4): 835-843.

[4] Movahedi K, Laoui D, Gysemans C, Baeten M, Stange G, Van den Bossche J, *et al.* Different tumor microenvironments contain functionally distinct subsets of macrophages derived from Ly6C(high) monocytes. *Cancer Res* 2010, **70**(14): 5728-5739.

[5] Laoui D, Van Overmeire E, Di Conza G, Aldeni C, Keirsse J, Morias Y, *et al*. Tumor hypoxia does not drive differentiation of tumor-associated macrophages but rather fine-tunes the M2-like macrophage population. *Cancer Res*. 2014; 74(1): 24-30

(references 25 and 26 in the manuscript). Laoui et al[4] (reference 26, Figures 1C and S1 in the manuscript) clearly demonstrated that the tumor-associated MHC-II^{hi} cells in this tumor model expressed lower levels of the macrophage markers F4/80, CD64 and MerTK, and higher levels of the costimulatory molecules CD80, CD86, PD-L1, PD-L2, and CD40. Moreover, only the MHC-II^{hi} cells strongly stimulate naive CD4^{+} and CD8^{+} T cells in a Mixed Leukocyte Reaction, while MHC-II^{mid} cells do not. In addition, employing the same gating strategy as shown in Figure 1A, we now showed that the MHC-II^{mid}CD11c^{+} cells express high levels of CD11b and markedly higher levels of the macrophage markers CD64 and F4/80 as compared to the MHC-II^{hi}CD11c^{+} cells (Figure R3, for the attention of the reviewer only). For these reasons, we strictly gated on MHC-II^{hi}CD11c^{+} cells to further study TADC in this manuscript.

The difference between the plots in Figure 1A and Figure 1B is mainly due to different tumor volumes, with a 12-day old tumor in Figure 1A and a 10-day old tumor in Figure 1B. This is now clarified in the Figure legend. We previously showed via kinetic analyses that smaller tumors contain less TAM[3], explaining the reduced presence of the MHC-II^{-mid} CD11c^{+} population in Figure 1B. Of note, we chose to perform the adoptive transfer experiments (Figure 1B in the manuscript) in mice carrying somewhat smaller tumors as it makes it easier to trace back larger amounts of transferred cells (the labeled cells are less “diluted” with other cells).

A second reason why the plots look different relies on the fact that the experiments were carried out on different flow cytometers using different cytometer settings, resulting in a more compressed view for both CD11c and MHC-II in Figure 1B.

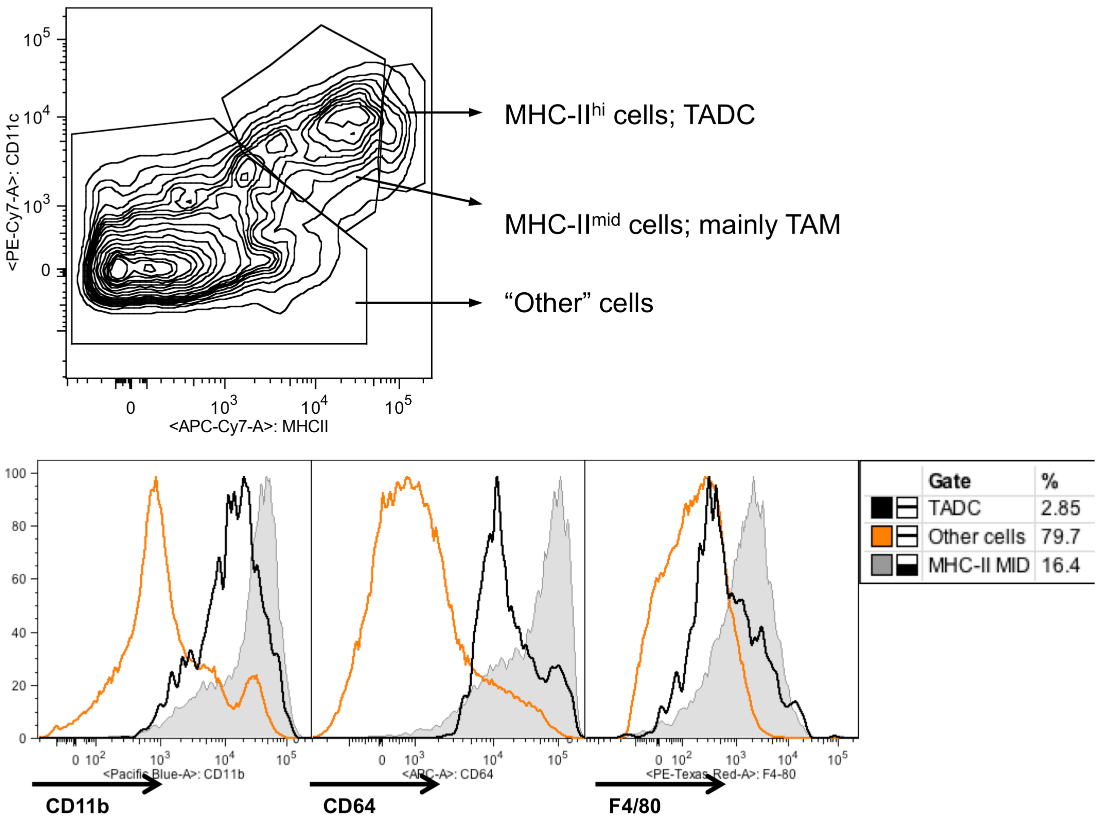

Figure R3. Marker expression of MHC-II^{mid} cells.

12-day old 3LL-R tumor single cell suspensions were pregated on Live/dead$^-$ CD45$^+$ Ly6G$^-$ CD3$^-$ NK1.1$^-$ CD19$^-$cells and subdivided based on the expression of CD11c and MHC-II in (i) MHC-II^{hi} TADC (black line), (ii) MHC-II^{mid} TAM (shaded histogram) and (iii) other cells (orange line). Histogram overlays compares the expression of the TAM markers CD11b, CD64 and F4/80 in the 3 populations. n=6.

5. In Figure 3, why was CD141 (BCDA3) not included in the analysis? IRF8 may be a substitute, but CD141 is more commonly found in the literature.

Reply: To identify the TADC subsets in human tumor biopsies, we indeed stained for both the cDC1-associated markers BDCA3 and IRF8. Within the CD45$^+$ CD3$^-$ CD19$^-$ CD56$^-$ BDCA2$^-$ CD16$^-$ CD11c^{high} HLA-DR$^+$ live cells, all the IRF8^{hi} cells are also BDCA3^{hi}, illustrating that human tumor cDC1 are double positive for BDCA3 and IRF8 (Figure R4A, new figure S3B in the manuscript). Importantly, no BDCA3$^+$ IRF8$^-$ nor BDCA3$^-$ IRF8$^+$ subsets were retrieved, demonstrating the interchangeability of both markers for identifying the cDC1 subset within the TADC compartment. These data are now also included in the Results section (p9) and shown in Figure S3B.
Notably, IRF8 expression provided the most clear-cut separation between cDC1 and other CD45$^+$ CD3$^-$ CD19$^-$ CD56$^-$ BDCA2$^-$ CD16$^-$ CD11c^{high} HLA-DR$^+$ cells (Figure R4B and R4C, for the attention of the reviewer only) and was hence preferred as the human cDC1 marker to be included in Figure 3 in the main text.

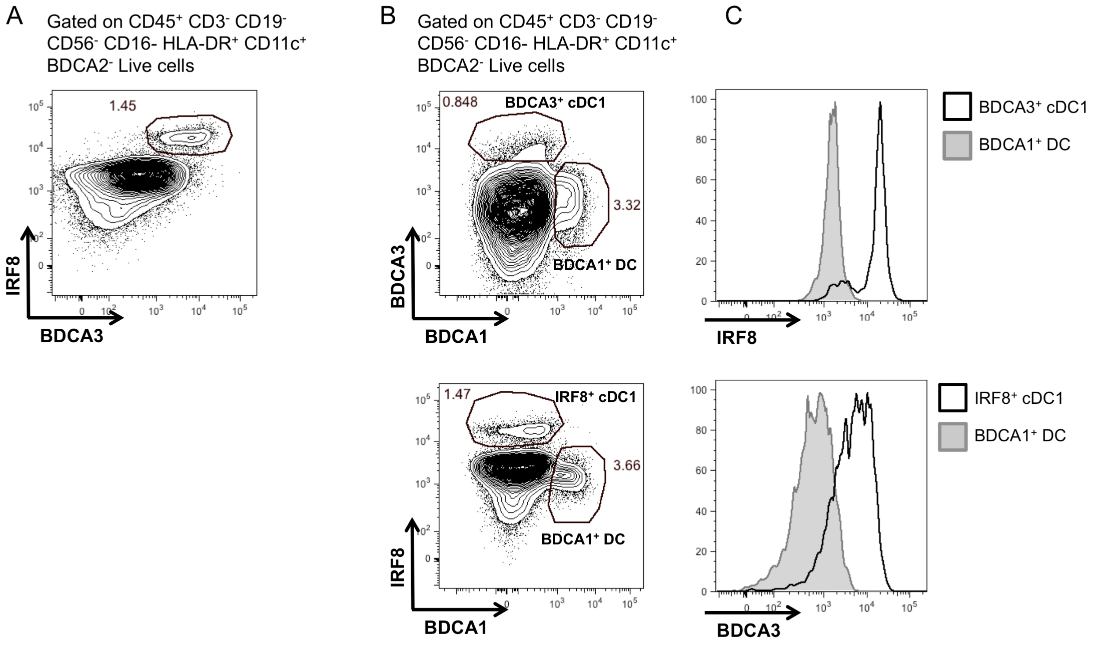

Figure R4: BDCA3 and IRF8 expression within the total human TADC population are interchangeable for the identification of the cDC1 TADC subset.
(A) Human non-small cell lung carcinoma (NSCLC) tumor biopsies were pre-gated on CD45$^+$ CD3$^-$ CD19$^-$ CD56$^-$ BDCA2$^-$ CD16$^-$ CD11c^{high} HLA-DR$^+$ live cells containing a distinct BDCA-3$^+$ IRF8$^+$ double positive cDC1 subset. (B) IRF8 expression provided the most clear-cut separation between cDC1 and other CD45$^+$ CD3$^-$ CD19$^-$ CD56$^-$ BDCA2$^-$ CD16$^-$ CD11c^{high} HLA-DR$^+$ live cells. (C) Histogram overlays are shown for the subsets delineated in (B). Black line = expression of IRF8 (upper panel) or BDCA3 (lower panel) on the cDC1 subset; shaded

histogram = expression of IRF8 (upper panel) or BDCA3 (lower panel) on the $BDCA1^+$ DCs. n = 4. IRF8 expression clearly shows the least overlap between cDC1 and other $CD45^+$ $CD3^-$ $CD19^-$ $CD56^-$ $BDCA2^-$ $CD16^-$ $CD11c^{high}$ $HLA\text{-}DR^+$ live cells.

6. In Figure 6, can the addition of moDC inhibit the response?

Reply: To assess whether tumor-derived Mo-DC have the capacity to suppress T-cell responses, we in first instance polyclonally stimulated naive syngeneic splenocytes in the presence of increasing amounts of Mo-DC. As shown in Figure R5A and R5B (new figures 6C and S7C in the manuscript), Mo-DC dose-dependently suppressed T-cell proliferation, while cDC2 sorted from tumors or splenic cDC did not. These data are described in the Results section, p 12. In addition, we demonstrated that the Mo-DC-mediated suppression of polyclonal T-cell proliferation is to a large extent dependent on the production of NO by these cells (Figure R5C, new figure 6E in the manuscript).
To assess Mo-DC's T-cell suppressive capacity in an antigen-specific system, we co-cultured tumor-derived cDC2 with purified, CellTrace-labeled TCR transgenic $CD4^+$ OT-II T cells in the presence of 250µg/ml Ovalbumin. cDC2 cultured for 3 days with OVA-stimulated OT-II T cells at a cDC2/OT-II ratio of 1/10 induced up to 43,2% of the $CD4^+$ T cells to proliferate (Figure R6, new figure 6D in the manuscript). However, addition of tumor-derived Mo-DC to these cultures strongly inhibited the cDC2-driven $CD4^+$ T-cell proliferation in a dose-dependent way, again demonstrating their suppressive phenotype. Notably, Mo-DC alone were not able to induce OT-II T-cell proliferation at any concentration tested, ranging from 1/5 to 1/40 Mo-DC/OT-II T cell ratios.

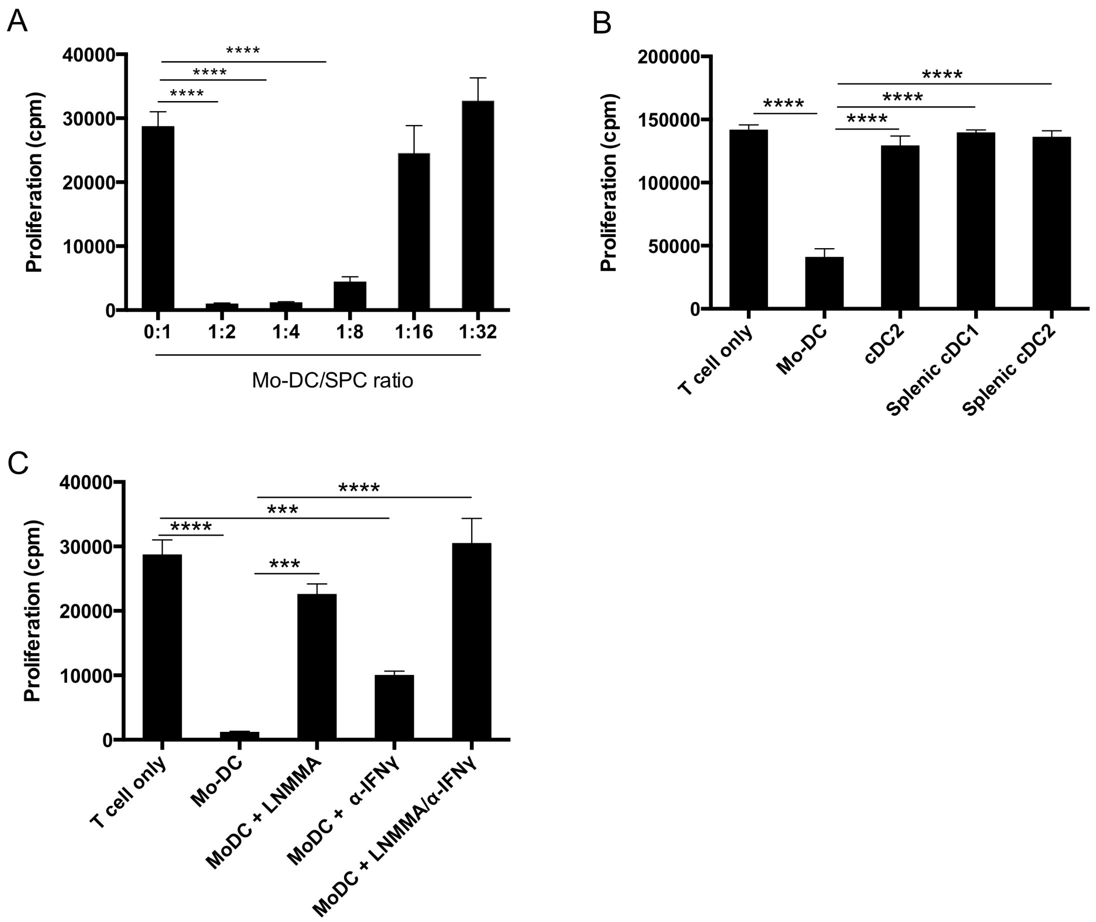

Figure R5: Mo-DC suppress polyclonally activated T-cell proliferation.

(A) Mo-DC were sorted from 12-day old 3LL-R tumor single cell suspensions and added at different ratios to splenocytes stimulated with anti-CD3/CD28-stimulated during 42h and the proliferation T cells was measured via ^{3}H-thymidine incorporation (cpm). Results are representative of 2 independent experiments with n = pool of 12 tumors. Statistical analysis by one-way ANOVA, ****, $p < 0.0001$. (B) Mo-DC and cDC2 sorted from 12-day old 3LL-R tumor single cell suspensions and cDC1 and cDC2 sorted from naive spleens were to splenocytes stimulated with anti-CD3 and anti-CD28 at a DC/SPC ratio of 1/4 during 42h and the T-cell proliferation was measured via ^{3}H-thymidine incorporation (cpm). n = pool of 12 tumors. Statistical analysis by one-way ANOVA. ****, $p < 0.0001$. (C) Sorted Mo-DC were co-cultured with OVA-stimulated splenocytes at a Mo-DC/SPC ratio of 1/4 with or without iNOS inhibitor (LNMMA) or α–IFNγ. T-cell proliferation was measured via ^{3}H-thymidine incorporation after 42h (cpm). n = pool of 12 tumors. Statistical analysis by one-way ANOVA, ***, $p < 0.001$; ****, $p < 0.0001$.

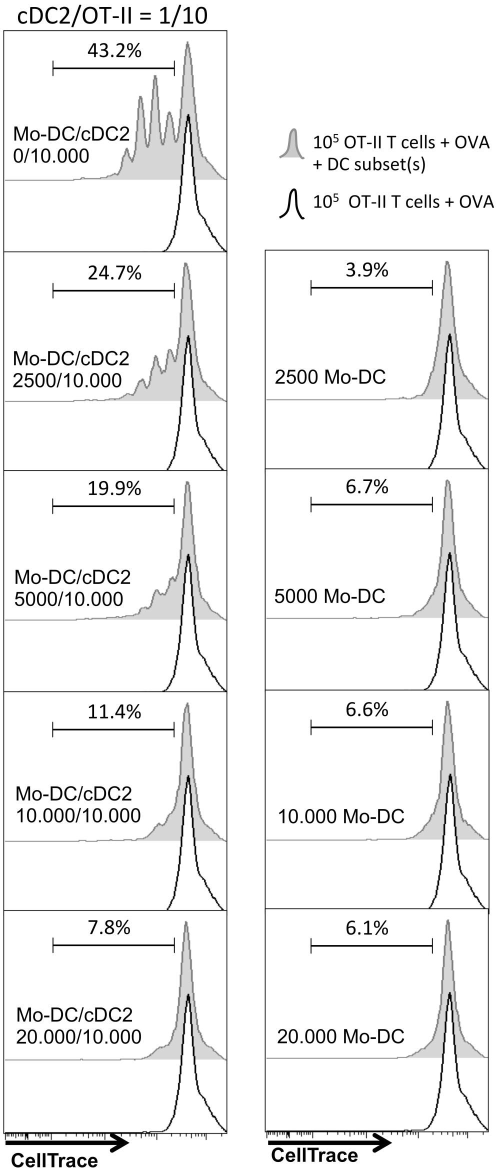

Figure R6: Mo-DC inhibit cDC2 driven CD4$^+$ T-cell proliferation.
Sorted Mo-DC were added at increasing concentrations to 10^5 OVA-stimulated OT-II T cells in the presence (left panels) or absence (right panels) of sorted cDC2 at a cDC2/OT-II ratio of 1/10. The histograms represent CellTrace dilution, indicative for T-cell proliferation. Black line = OVA-stimulated T cells without TADC; shaded histogram = OVA-stimulated T cells in the presence of TADC. n = pool of 12 tumors.

7. In figure 8, were DC injected at the same site as tumor inoculation? Have resident memory T cells been created?

Reply: Since the tumor-derived DC were injected subcutaneously, hence at the same site as tumor inoculation, and not in any particular organ, it will be impossible to assess the presence of antigen-specific resident memory T cells at this rather undefined site.

Reviewer #2:
In this manuscript, using murine and human tumor models, the authors demonstrated that tumors harbored ontogenically discrete TADC subpopulations. Monocyte-derived TADC were prominent in TA processing, but were not strong T-cell stimulators due to NO-mediated immunosuppression. Pre-cDC-derived TADC displayed the ability to migrate to the lymph nodes, while cDC1 activated CD8 T cells and cDC2 induced Th17 cells. Mice vaccinated with cDC2 demonstrated an inhibited tumor growth with a reprogramming of pro-tumor TAM and a reduction of MDSC. Vaccination with cDC1 induced antitumor CTL. The authors concluded targeting specific TADC subsets or their precursors might be important for improved therapeutic interventions.

The authors have used interesting and elegant experiments to determine TADC origin in mice. For instance, utilization of CCR2-deficient mice is a great idea and confirmed the existence of ontogenically different TADC populations. In line with this, utilization of Flt3L-KO and GM-CSFR-KO mice is also a strength of the manuscript. As a result, the authors unraveled the coexistence of ontogenically and functionally distinct TADC subsets and determined their effect on T cells in vitro and the effect on tumor growth in vivo. In vivo analysis of DC vaccines in different tumor models is a magnificent part of the manuscript as it additionally adds the involvement of TAM and MDSC. This part can be an individual paper. The minor concern is that the use of term "therapeutic effect" (or similar) is misleading. The authors did not use the 'therapeutic' tumor models, but utilized 'prophylactic' tumor models where DC were injected prior to tumor cell administration. Thus, only protective effect of DC vaccines was determined, not a therapeutic effect. It will be helpful if the authors improve this part of Results and correct Discussion accordantly. It seems somehow inappropriate to discuss therapeutic DC vaccines without presenting any related data in the manuscript.

Reply: We fully agree with the reviewer that the set-up of our vaccination experiments is prophylactic and not therapeutic. Therefore, all references to “therapeutic effects” have been removed from titles, from the Results section and from the Discussion. In the Discussion, we still speculate on the potential relevance of our prophylactic findings for future therapeutic settings.

- Overall, the results are very interesting, innovative and may have clinical significance. There are a lot of results and are very well presented, although all figures look quite busy.

Reply: We agree with the reviewer’s remark. To make the figures less busy and to highlight only the most important findings, some figure panels were moved to supplemental figures. This was done for Figures 2, 4, 5, 6 and 8.

- The only question is about immunosuppressive/tolerogenic TADC which were described in numerous publications. The authors did not reveal any DC subsets with strong immunosuppressive properties: NO blockage experiments are not related since immunosuppression was not revealed (no experiments analyzed the effect of DC on pre-activated T cells). Non-functional or functionally deficient TADC have been repeatedly described, but have not been seen in these studies. All TADC

subsets were functionally active, although affect different targets. It will be very helpful if this important issue is explained in the manuscript.

Reply: To assess whether tumor-derived Mo-DC have the capacity to suppress T-cell responses, we in first instance polyclonally stimulated naive syngeneic splenocytes in the presence of increasing amounts of Mo-DC. As shown in Figures R5A and R5B (new figures 6C and S7C in the manuscript), Mo-DC dose-dependently suppressed T-cell proliferation, while cDC2 sorted from tumors or splenic cDC did not. These data are described in the Results section, p 12. In addition, we demonstrated that the Mo-DC-mediated suppression of polyclonal T-cell proliferation is to a large extent dependent on the production of NO by these cells (Figure R5C, new figure 6E in the manuscript).
To assess Mo-DC's T-cell suppressive capacity in an antigen-specific system, we co-cultured tumor-derived cDC2 with purified, CellTrace-labeled TCR transgenic $CD4^+$ OT-II T cells in the presence of 250µg/ml Ovalbumin. cDC2 cultured for 3 days with OVA-stimulated OT-II T cells at a cDC2/OT-II ratio of 1/10 induced up to 43,2% of the $CD4^+$ T cells to proliferate (Figure R6, new figure 6D in the manuscript). However, addition of tumor-derived Mo-DC to these cultures strongly inhibited the cDC2-driven $CD4^+$ T-cell proliferation in a dose-dependent way, again demonstrating their suppressive phenotype. Notably, Mo-DC alone were not able to induce OT-II T-cell proliferation at any concentration tested, ranging from 1/5 to 1/40 Mo-DC/OT-II T cell ratios.
Together, these new data firmly establish tumor-associated Mo-DC as cells with immunosuppressive properties.

- Inclusion of human tumor specimens and human TADC isolation is an interesting part of the manuscript, but this direction has not been developed and one can suggest omitting this part from the manuscript. This is just a suggestion.

Reply: We are in favor of keeping the human data as part of the manuscript. We believe that inclusion of human data strengthens the clinical relevance of our findings. Since the TADC subsets identified in human tumor biopsies were similar to the TADC that were functionally characterized in mice, our findings might prove important for future therapeutic interventions targeted at specific TADC subsets.

Minor comments:
- Description of DC ontogenesis in Introduction does not, even briefly, explain differences between murine and human DC subsets.

Reply: We now included a brief comparison between murine and human DC subsets in the Introduction. Following text was added (p4):
"Importantly, transcriptomic analysis of mouse and human DC subsets revealed that human $CD141(BDCA3)^+$ DC are related to mouse cDC1, whereas human

CD1c(BDCA1)$^+$ DC are more related to mouse cDC2 [6] (reference 20 in the manuscript). Human CD141$^+$ DCs express Batf3 and IRF8 and lack expression of IRF4, akin to mouse cDC1. Moreover, the differentiation of human hematopoietic progenitors into CD141$^+$ DCs occurs only when Flt3L is added to the cultures, and inhibition of Batf3 in these cultures abolishes the differentiation of CD141$^+$ DC but not of CD1c$^+$ DC, suggesting that CD141$^+$ DC are indeed developmentally related to mouse cDC1."

- The overall characterization of TADC is based on the not-widely-accepted classification of macrophages, which does not utilize CD11c expression. In addition, it has been recently reported that tolerogenic TADC may differentiate from MDSC and therefore express Gr1. There is not doubts that different classifications of DC and TADC exists, and it will be helpful to justify selection of either one.

Reply: The reviewer correctly points out that different DC classification systems and DC nomenclatures can be found in literature. We are in favor of a classification system based on the ontogeny of the cells, as proposed by Guilliams *et al*[7] (reference 15 in the manuscript). The presence of ontogenically distinct DC subsets, having cDC progenitors or monocytes as precursor, in the tumor microenvironment is one of the main messages of the manuscript, justifying the use of this classification system.
cDC1 have also been termed CD8α^+-like or CD103$^+$ conventional DC, whereas cDC2 are also known as CD11b$^+$-like conventional DC. We mention the various names in the Introduction and stick to the cDC1/cDC2 nomenclature throughout the manuscript.
We now point out the existence of distinct DC classification systems and justify the use of this classification system in the Introduction.

- It is not quite correct to conclude about the antigen uptake ability of DC utilizing a single phagocytosis assay, since different DC subsets display differential ability for phagocytosis, pinocytosis and receptor-mediated endocytosis (all of these may be involved in Ag uptake). One can suggest a careful re-phrasing of this part of Results. Of note: this issue is of importance since in the next part of Results, the authors used DQ-ovalbumin, which is taken up by pinocytosis and has nothing to do with bead phagocytosis.

Reply: We agree with the reviewer's comment. To unravel which TADC subset had the highest OVA uptake capacity, we added fluorescently labeled OVA-AF488 *in vitro* to 3LL-R tumor single cell suspensions at 37°C (active Ag-uptake) or at 4°C (Figures

[6] Merad M, Sathe P, Helft J, Miller J, Mortha A. The dendritic cell lineage: ontogeny and function of dendritic cells and their subsets in the steady state and the inflamed setting. *Annual review of immunology* 2013, **31:** 563-604.

[7] Guilliams M, Ginhoux F, Jakubzick C, Naik SH, Onai N, Schraml BU*, et al.* Dendritic cells, monocytes and macrophages: a unified nomenclature based on ontogeny. *Nature reviews Immunology* 2014, **14**(8)**:** 571-578

R7A and R7B, new figures 4A and 4B in the manuscript). All TADC subsets were able to ingest OVA at 37°C. However, when compared to the total TADC population, the proportion of cells within the OVA$^+$ TADC population only increased for the Mo-DC subset, signifying that this population had, besides a higher inherent phagocytic capacity (as shown via bead uptake), also a higher Ag uptake capacity via pinocytosis than both cDC types (Figure R7B, new figure 4B in the manuscript). Hence, the results of the Ag uptake assay were very similar to the results of the phagocytosis assay (Figure R7C and R7D, figure S4A and S4B in the manuscript) and suggest that the Mo-DC are better in scavenging several types of antigens compared to the cDC subsets.

As we believe that the OVA uptake is more relevant in the context of tumor antigens than the general phagocytic capacity of DC, these new results were added in main Figure 4 and the text in the manuscript was adapted accordingly, while the phagocytosis assays were moved to the Supplementary Information (Figures S4A, S4B and S4C in the manuscript).

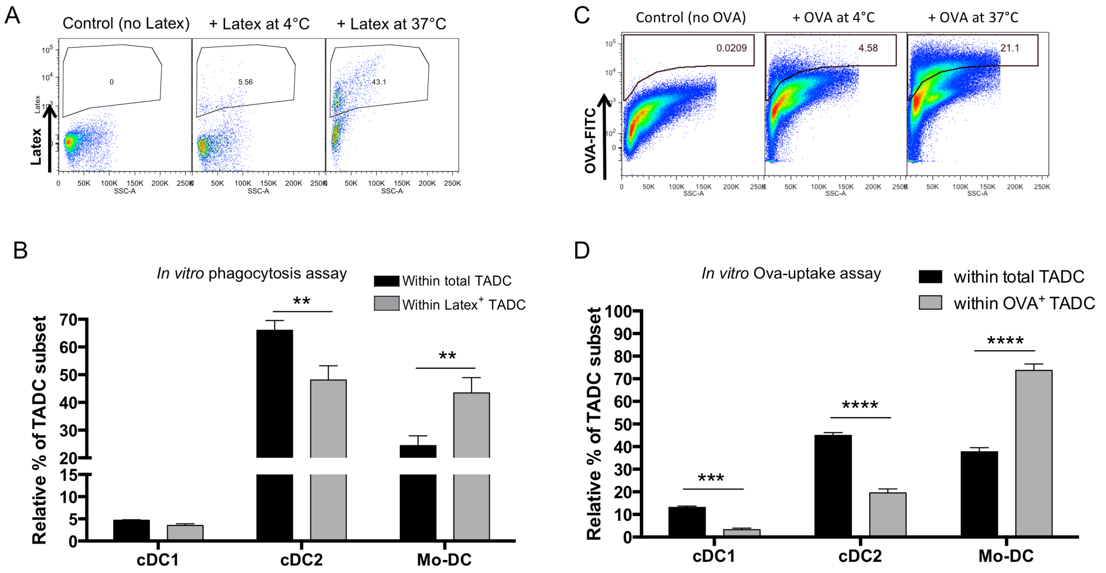

Figure R7: Mo-DC have a higher phagocytic and OVA-uptake capacity

(A-B) *In vitro* phagocytosis assay. (A) Single cell suspensions of 12-day old 3LL-R tumors were cultured *in vitro*, in the absence (control) or presence of latex beads for 40 minutes at 4°C or 37°C. (B) The percentage of the distinct TADC subsets within the total TADC gate or within the Latex$^+$ TADC gate are given. n=3 pools of 4 tumors. Statistical analysis by one-way ANOVA. **, $p < 0.01$. (C-D) *In vitro* OVA-uptake assay. (C) Single cell suspensions of 12-day old 3LL-R tumors were cultured *in vitro*, in the absence (control) or presence of OVA-AF488 for 15 minutes at 4°C or 37°C. (D) The percentage of the distinct TADC subsets within the total TADC gate or within the OVA$^+$ TADC gate are given. Results are representative of 2 independent experiments with n≥4. Statistical analysis by one-way ANOVA. ***, $p < 0.001$; ****, $p < 0.0001$.

- What was the level of DQ-ovalbumin uptake by different TADC subsets? May be this can answer the previous concern? Can the differences in DQ-ovalbumin processing be explained by the differences in DQ-ovalbumin uptake?

Reply: In the antigen uptake assay, the percentage of fluorescently labeled cells, which correspond to the level of fluorescently labeled OVA (OVA-AF488) taken up after 15 min of incubation, were 73,7% ± 6,9% for the Mo-DC; 3,3% ± 1,6% for the cDC1 and 19,5% ± 4,3% for the cDC2 (Figure R7B, new figure 4B in the manuscript). To assess the rate of antigen processing, 3LL-R tumor single cell suspensions were first incubated with DQ-ovalbumin without washing. During the first 15 min (15+0 on the graph), the percentage of fluorescently labeled cells is a measure for the combined effect of Ag uptake and Ag processing by the TADC subsets in that timeframe. These percentages were 62,7% ± 3,5% for the Mo-DC; 1,5% ± 0,9% for the cDC1 and 5,3% ± 1,3% for the cDC2 (Figure 4C in the manuscript).
Interestingly, these percentages were in the same range as the percentages seen with the OVA-AF488 uptake assay, suggesting indeed that an enhanced Ag uptake by Mo-DC may contribute significantly to the enhanced DQ-OVA “processing” by these cells during the first 15 min of the assay.
The conclusions in the manuscript were adapted accordingly, whereby focus is put on the enhanced antigen uptake capacity of Mo-DC. References to the enhanced antigen processing capacity of these cells were omitted.

- To suggest that any TADC subset can inhibit T cell proliferation, may be the direct way is to mix TADC with pre-activated proliferating T cells.

Reply: We clearly demonstrated the immunosuppressive/anti-proliferative capacity of Mo-DC, as outlined in a previous answer (cfr Figure R5).

In response to Reviewer 1, who wondered about the capacity of TADC to stimulate pre-activated T cells, we sorted TADC from LLC-OVA tumors and co-cultured them with $CD4^+$ or $CD8^+$ T cells purified from the tdLN of 12-day old LLC-OVA bearing mice. In these tdLN, the presence of antigen-experienced T cells can be anticipated. Interestingly, freshly isolated cDC2 and Mo-DC (insufficient cDC1 could be purified for this experiment) were able to restimulate *in vivo* primed tdLN-derived $CD4^+$ or $CD8^+$ T cells, whereby cDC2 were significantly better in triggering T-cell proliferation than Mo-DC (Figures R1A and R1B, new figure S8C in the manuscript). To confirm the T-cell restimulating capacity of cDC2 in an alternative setting, we co-cultured freshly isolated cDC2 with purified $CD4^+$ or $CD8^+$ T cells from the spleen of mice that were vaccinated with OVA in Complete Freund’s Adjuvant (CFA). Again, cDC2 were shown to restimulate these *in vivo* primed T cells (Figures R1C and R1D, for the attention of the reviewer only).
Together, these data illustrate that at least tumor-associated cDC2 have the capacity to re-activate antigen-primed $CD4^+$ and $CD8^+$ T cells. Mo-DC are much less efficient in doing this, in line with their immunosuppressive properties.

- It is quite hard to understand the activating/inhibitory activity of TADC subsets without comparing the results with a "classic gold standard" - bone marrow-derived DC. Is this possible?

Reply: We decided to use conventional splenic DC as a “classic gold standard”, rather than bone marrow-derived DC, since the latter were recently shown to be a heterogeneous group of cells that comprises conventional DC and monocyte-derived macrophages[8].
cDC1 and cDC2 from naive spleens were discriminated using the gating strategy depicted in figure R8A (new figure S6A in the manuscript) and sorted to high purity. These cells were shown to express high levels of the costimulatory molecules CD80, CD86, PDL1 and PDL2 (Figure R8B, new figure S6B in the manuscript).
The manuscript already contained data showing that all TADC subsets could activate naive Balb/c $CD4^+$ and $CD8^+$ T-cell proliferation in a mixed leukocyte reaction, to the same extent as the control splenic $CD11c^{hi}MHC\text{-}II^{hi}B220^-Ly6C^-$ cDC population (Figures S5B and S5C in the manuscript). In a side-by-side comparison, we now co-cultured cDC1 and cDC2 sorted from LLC tumors or naive splenic cDC1 and cDC2 with CellTrace-labeled TCR transgenic $CD8^+$ OT-I T cells or $CD4^+$ OT-II T cells in the presence of ovalbumin protein. At a DC/OT-I ratio of 1/10, tumor-derived cDC subsets could induce $CD8^+$ OT-I T-cell proliferation to the same extent as their splenic cDC counterparts (Figure R8C, new figure S6C in the manuscript). Interestingly, in the case of $CD4^+$ OT-II T-cells, the highest proliferation was measured when T cells were co-cultured with tumor-derived cDC2 (Figure R8D, new figure S6D in manuscript). Moreover, the Th17 inducing cytokine IL-23 and the Th17 product IL-17 were mainly detected in the supernatans of the co-cultures containing tumor-derived cDC2 (Figure R8E, Figure S6E in the manuscript).
In conclusion, tumor-derived cDC1 are as potent as “classic” splenic cDC1 in stimulating $CD8^+$ T-cell activation, while tumor-derived cDC2 are even superior to splenic cDC for activating $CD4^+$ T cells and are unique in their Th17-inducing capacity.

[8] Helft J, Bottcher J, Chakravarty P, Zelenay S, Huotari J, Schraml BU, *et al.* GM-CSF Mouse Bone Marrow Cultures Comprise a Heterogeneous Population of CD11c(+)MHCII(+) Macrophages and Dendritic Cells. *Immunity* 2015, **42**(6): 1197-1211.

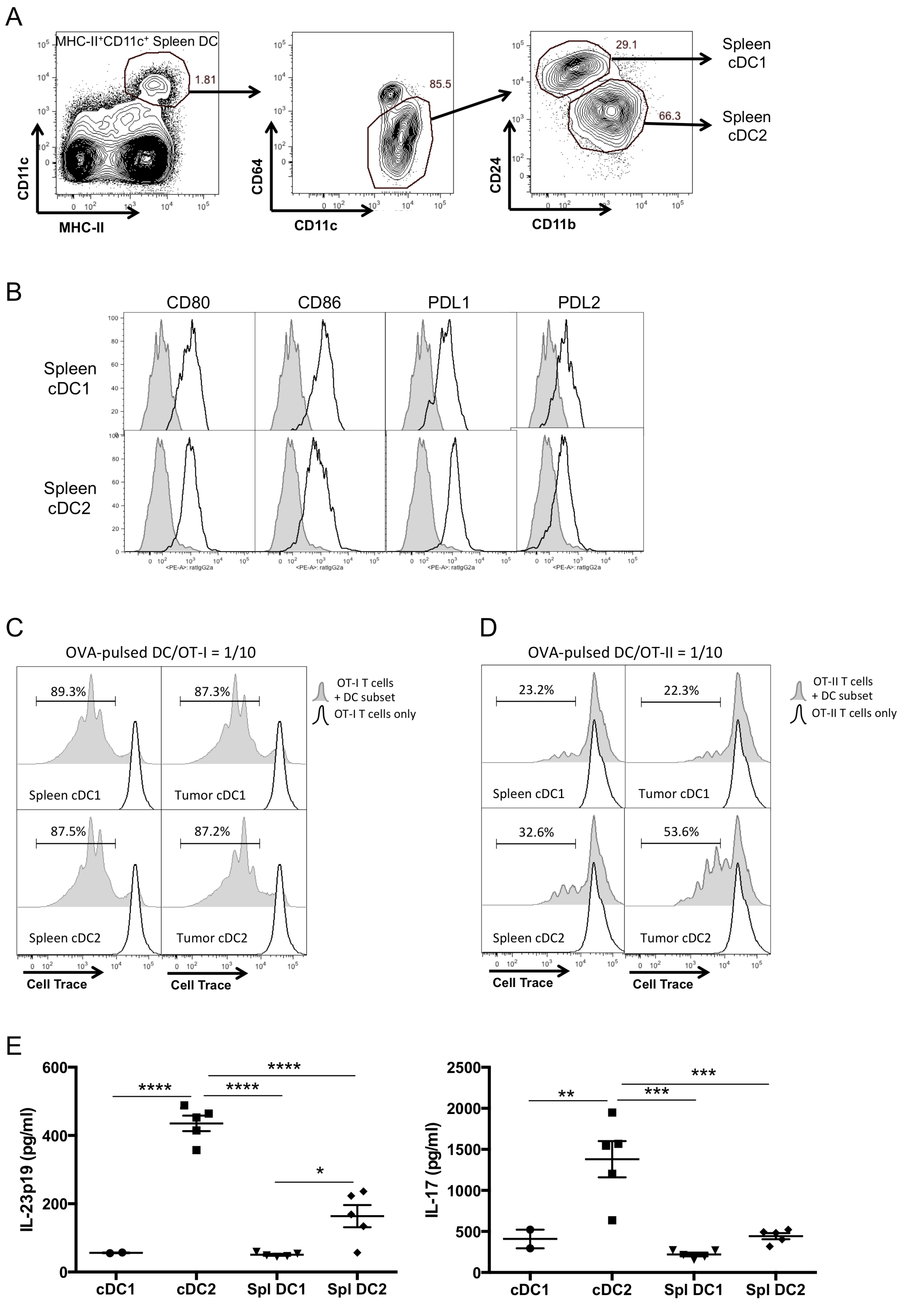

Figure R8: Tumor-derived cDC display equal to higher T-cell activating capacities as compared to splenic cDC.

(A) Splenic single cell suspensions of naive mice were gated for CD64^{lo} MHC-II^{hi} CD11c^{hi} cells and subdivided in (i) CD24^{pos} CD11b^{lo} cDC1 and (ii) CD24^{low} CD11b^{pos} cDC2. (B) Single cell suspensions of naive spleens were stained for the indicated markers and histogram overlays are shown. Black line = expression of the indicated marker; shaded histogram =

isotype control. n=4. (C-D) Sorted TADC or splenic cDC subsets were co-cultured with OT-I (C) or OT-II (D) T cells for 3 days at a DC/T-cell ratio of 1/10 in the presence of OVA. The histograms represent CellTrace dilution, indicative for T-cell proliferation. Black line = non-stimulated T cells without DC; shaded histogram = T cells in the presence of DC. Results are representative of 2 independent experiments with n = pool of 12 tumors. (E) Supernatants of co-cultures of TADC or splenic DC subsets and OT-II T cells (DC/OT-II = 1/10) were tested for the presence of IL-23 and IL-17 by luminex. n=5 . Statistical analysis by one-way ANOVA. *, $p < 0.05$; **, $p < 0.01$; ***, $p < 0.001$; ****, $p < 0.0001$.

- Did the authors used soluble "1 µg/ml anti-CD3 and 2 µg/ml CD28" Abs for T cell stimulation? Did it work? Isn't it important to use bead- or surface-coated Abs?

Reply: Most commercially available anti-CD3 antibodies do not stimulate T-cell proliferation optimally when used in solution and in most cases polyclonal T-cell proliferation is enhanced when the plates are coated overnight with anti-CD3 Ab before adding the T cells.

For this manuscript, we used in-house purified Ab from an anti-CD3 producing hybridoma (clone 145-2C11). This Ab provides the same results when added in the medium together with the T cells or when pre-coated on the plates.

The anti-CD28 Ab (clone 37.51) was purchased from eBioscience (cat N° 16-0281-85) and gives similar results when used surface-coated or in solution.

-Introduction of TADC might be slightly extended to introduce different TADC subsets and function (just a suggestion).

Reply: We now significantly extended the Introduction with a comparison between mouse and human DC populations. Published work on the regulation and functions of TADC was already incorporated in the Introduction of the manuscript, albeit briefly (references 4, 9, 10; p4).

Due to space restrictions, we included only one additional sentence to the Introduction, hinting to the existence of distinct TADC populations: “In addition, tumor-associated DCs (TADCs) may favor tumor progression by mediating genomic damage, supporting neovascularization and stimulating cancerous cell growth and spreading, features that may be attributed to the existence of distinct TADC populations.”

Reviewer #3:

The first part of this manuscript contains a detailed phenotypic and precursor analysis of 3 different subpopulations of tumor-associated Dendritic Cells, demonstrated to be present both in different mouse tumor models and in human lung and colorectal cancers. The analysis appears as carefully performed, and the fact that these 3 distinct subsets can be defined at various frequencies in several different tumor types is of interest particularly to those in the DC field. But the biological implications of this finding, particularly when it comes to therapeutic effects for tumor vaccination, is less convincing, as commented on below.

- Many of the observations, although of considerable interest, are quite superficial and would need a more thorough analysis in order to have a more profound impact on our knowledge of DC subsets. For example, to what extent does the variation of the different subsets shown in Fig 1C depend on their different ability to migrate to the tumor, as compared to their capacity to survive there?

Reply: For the current manuscript, our main interest was to prove the presence and immunogenicity of ontogenically distinct DC in the tumor microenvironment through adoptive transfer of committed precursors (cDC precursors or monocytes) and the use of mice which lack important regulators of DC differentiation. We consider the detailed study of DC dynamics and fine-tuning as part of a future investigation.
CCR2-KO mice have strongly reduced monocyte numbers in their circulation, resulting in a paucity of monocyte-derived cells in these mice. Hence, the lack of Mo-DC, but not cDC, in CCR2-KO mice is due to a lack of monocyte attraction to the tumor site.
Flt3L-KO mice were used since these mice contain strongly reduced $CD8^+$ cDC (cDC1). In addition, similar to cDC1, $CD11b^+$ cDC (cDC2) proliferate *in situ* in response to Flt3L, and are reduced in Flt3L-deficient mice, albeit to a lesser extent than cDC1[9]. Our data are consistent with these findings, with a full dependence of tumor-associated cDC1 on Ftl3L and only a partial dependence for cDC2. Unexpectedly, also the presence of Mo-DC is heavily dependent on Flt3L, which either means that Flt3L directly contributes to Mo-DC differentiation in tumors or that Mo-DC generation depends on the presence of cDC.
GM-CSFR-KO mice were relevant for this study, since the survival of cDC1 and to a lesser extent also cDC2 depends on GM-CSFR signaling, while inflammatory Mo-DC were reported to be GM-CSFR-independent[10]. This is again reflected in our data, whereby tumor-associated cDC1 are nearly absent in GM-CSFR-KO mice and cDC2 are only partially affected. In the tumor context, inflammatory Mo-DC are also partially dependent on GM-CSFR or, as suggested above, on the on the presence of cDC.
Overall, we believe to have clearly and unequivocally demonstrated the presence of ontogenically distinct DC subsets at the tumor site. Though further issues on the dynamics of these DC populations are very interesting, we consider those beyond the scope of the current manuscript.

- The tumor protection data after vaccination with the different DC subsets as shown in Fig 8 B and Fig 10 shows that the protection is not very impressive, particularly when compared to the effect of vaccination with Ova.

---

[9] Merad M, Sathe P, Helft J, Miller J, Mortha A. The dendritic cell lineage: ontogeny and function of dendritic cells and their subsets in the steady state and the inflamed setting. *Annual review of immunology* 2013, **31:** 563-604.

[10] Greter M, Helft J, Chow A, Hashimoto D, Mortha A, Agudo-Cantero J, *et al.* GM-CSF controls nonlymphoid tissue dendritic cell homeostasis but is dispensable for the differentiation of inflammatory dendritic cells. *Immunity* 2012, **36**(6)**:** 1031-1046

Reply: Immunizations with a rather high dose of a pure immunodominant antigen (OVA) admixed with a particularly potent adjuvant such as CFA (an adjuvant that is forbidden for human use due to its too high potency) is expected to induce very strong anti-tumor T-cell responses. Therefore, we used this condition as a positive control in our assays.
TADC vaccinations make us of a very low amount (only 10^4 cells!) of freshly purified cDC1 or cDC2 from the tumor microenvironment. These cells were not further matured *in vitro*, nor did they receive an additional loading with the tumor antigen OVA. The aim was indeed to evaluate the TADC's inherent capacity to induce relevant anti-tumor T-cell responses, rather than to generate an optimal anti-cancer vaccine at this point. We believe that the anti-tumor effects of the TADC vaccinations are quite significant, given the low inoculum and the fact that they remained unmanipulated. That being said, we are aware that future research will be required to optimize the dosage, regimen of administration, route of administration, etc to further enhance the efficacy of TADC vaccines.

- Also they must analyze this tumor protection more in detail, e.g. if this is a CD8 T cell mediated effect, by performing depletion of CD8 cells in vivo. DC cells are known to have effects on NK cells and it cannot be excluded that the observed effects are NK mediated.

Reply: To unravel which cellular mediators are implicated in the reduced tumor growth following TADC vaccination, we vaccinated mice with cDC2 and depleted $CD8^+$ T cells (using anti-CD8 mAb), $CD4^+$ T cells (anti-CD4 mAb) and NK/NKT cells (anti-NK1.1 mAb) *in vivo* starting from tumor inoculation.

First, antibody-mediated depletion of $CD4^+$ T cells (Figure R2A, new figure S10A in the manuscript) was shown to revert the retarded tumor growth upon cDC2 vaccination, suggesting that $CD4^+$ T cells are protective in this setting (Figure R2B, new figure S10C in the manuscript. This is remarkable, since $CD4^+$ T-cell depletion lowers tumor growth in non-vaccinated mice, indicating that cDC2 vaccination switches the $CD4^+$ T-cell pool from mainly pro-tumoral to mainly anti-tumoral.
Since cDC2 vaccination induced a $CD4^+$ Th17 T-cell response (Figure 8D in the manuscript), we assessed the specific involvement of Th17 cells in the anti-tumor response generated by cDC2-vaccination. To this end, IL-23p19-deficient mice, which lack Th17 cells[11] (IL-23 being a crucial cytokine for Th17 induction), were vaccinated. The slower growth rate induced by cDC2 vaccination was totally abrogated in IL-23p19-deficient mice, indicating that Th17 cells play a non-redundant role in the anti-tumor protection conferred by cDC2 vaccination (Figure R2C, new figure 8E in the manuscript).
When depleting $CD8^+$ T cells, the tumor growth rate was unaltered in non-vaccinated mice. However, after cDC2-vaccination, the tumor growth rate partially recovered in CD8-depleted mice, albeit to a lesser extent than upon $CD4^+$ T-cell depletion

[11] Ghilardi N, Kljavin N, Chen Q, Lucas S, Gurney AL, de Sauvage FJ. Compromised Humoral and Delayed-Type Hypersensitivity Responses in IL-23-Deficient Mice. *The Journal of Immunology* 2004, **172**(5): 2827-2833

(Figures R2B, R9A, R9C and R9C, new figure S10A, S10B, S10C and S10D in the manuscript). These data may suggest that CD8$^+$ T-cell activity state (as shown by enhanced IFNγ production, Figure R9D, new figure S10E in the manuscript) becomes more prominent after cDC2 vaccination, which is in accordance with the reprogramming of immunosuppressive M2-like TAM to less immunosuppressive M1-like TAM (Figures 9E, 9F and 9G in the manuscript).
We next assessed whether NK or NKT cells were implicated in the anti-tumor effect of cDC2 vaccination. The amount of NK cells was slightly increased in tumors from cDC2-vaccinated mice, while NKT cell numbers remained unaffected (Figure R10A, new figure S11D in the manuscript). However, the NK cells present in tumors from cDC2-vaccinated mice produced very low levels of IFN-γ, as was the case for non-vaccinated mice, suggesting only a marginal activation of NK cells in LLC-OVA tumors (Figure R10B, new figure S11E in the manuscript). Moreover, the tumor growth rate of cDC2-vaccinated mice was unaltered when depleting NK and NKT cells using anti-NK1.1 Ab, suggesting that NK and NKT cells were not involved in the anti-tumor effect of cDC2 vaccination (Figures R10C, R10D, R10E, new figures S11A, S11B and S11C in the manuscript).

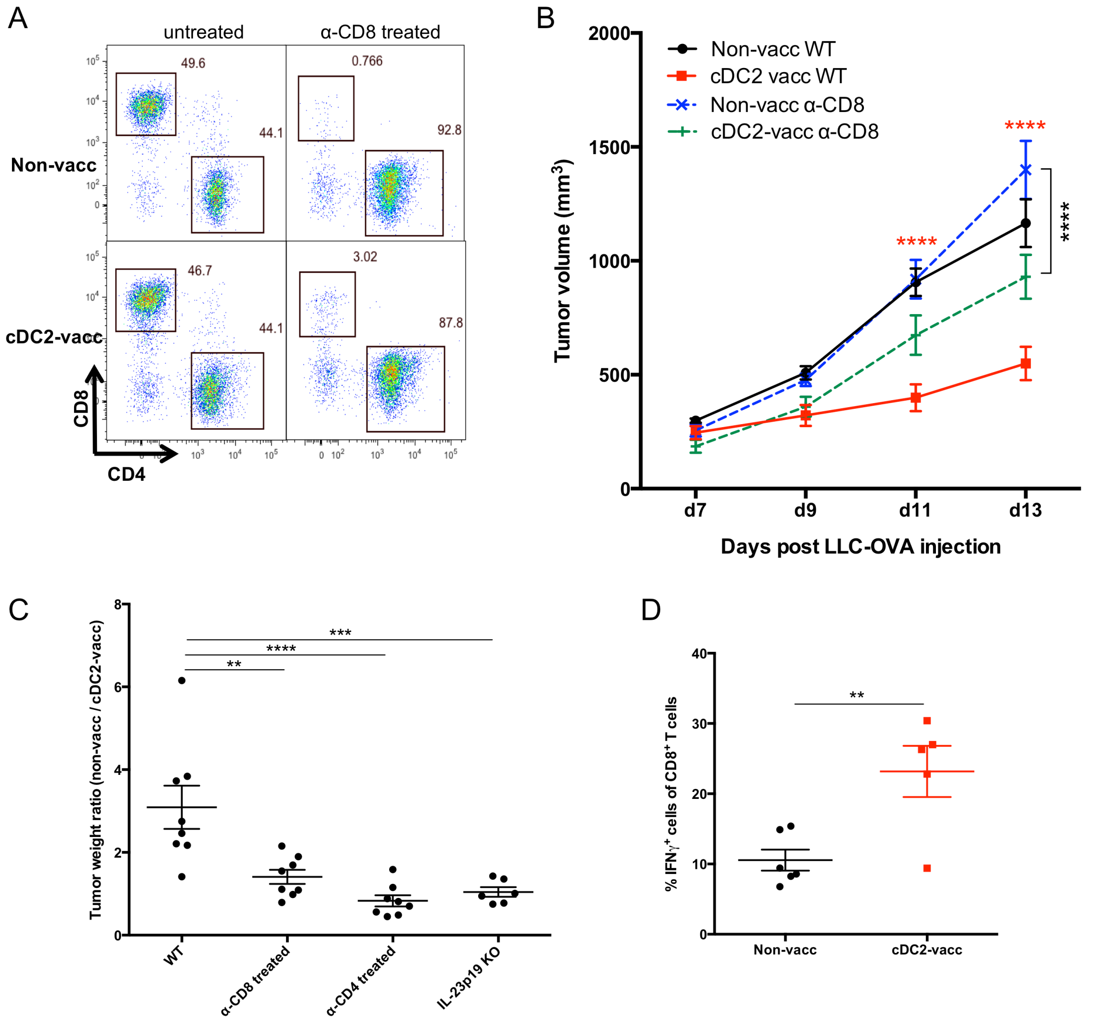

Figure R9: Effect of cDC2 vaccination after CD8 T-cell depletion.

(A) Non-vaccinated or cDC2-vaccinated mice were treated every 2-3 days as from the day before tumor inoculation with anti-CD8 depleting Ab. Representative plots of blood of 13-day old LLC-OVA tumor-bearing mice gated on $CD45^{+}$ $Tcrb^{+}$ cells show the amount of CD8 T cells. (B) Growth curve of LLC-OVA tumors after vaccination with LLC-OVA-derived cDC2 in untreated and anti-CD8 treated mice. (C) Tumor weight ratio (non-vacc / cDC2-vacc) of LLC-OVA tumors after vaccination with LLC-OVA-derived cDC2 in WT, anti-CD8 treated, anti-CD4 treated and IL-23p19 KO mice. (D) Percentages of $IFN\gamma^{+}$ $CD8^{+}$ T cells in LLC-OVA tumors after vaccination with LLC-OVA-derived cDC2 following the protocol depicted in (Figure 8A). n ≥ 6. Statistical analysis by one-way ANOVA. *, $p < 0.05$; **, $p < 0.01$; ***, $p < 0.001$; ****, $p < 0.0001$.

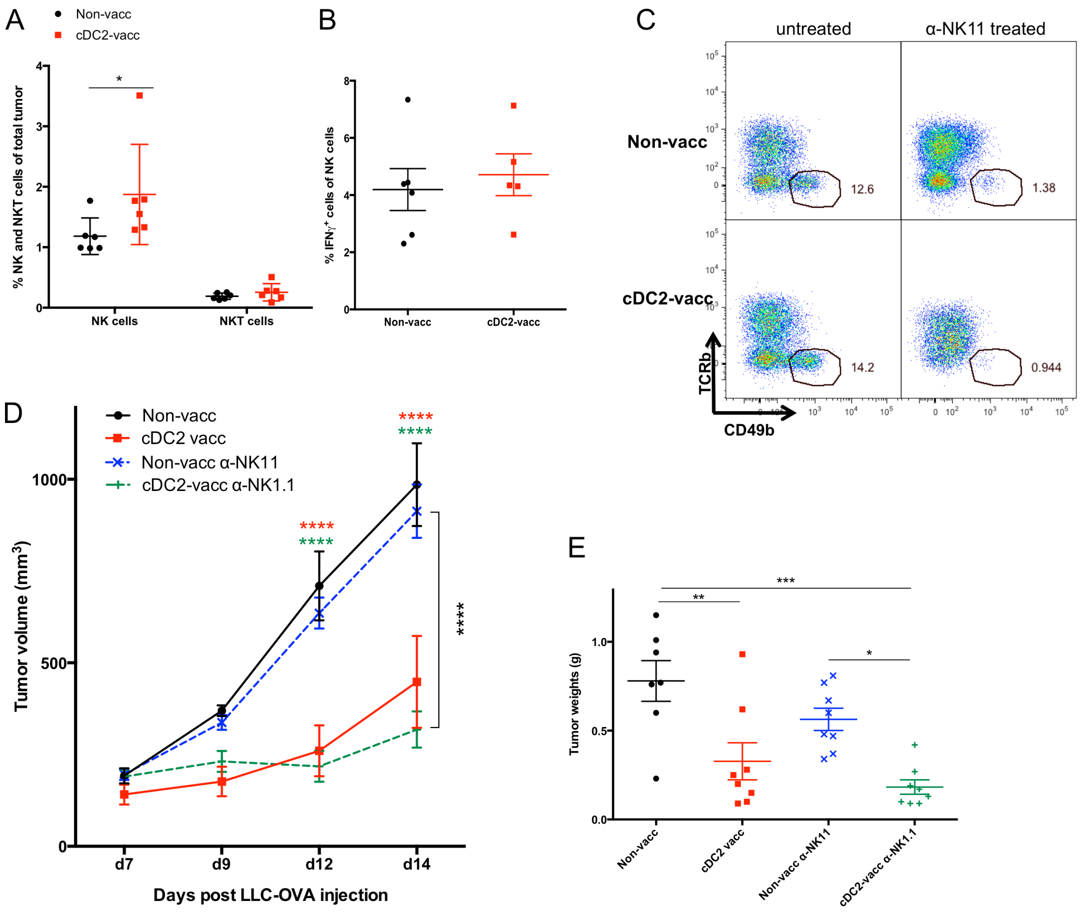

Figure R10: NK cells are not involved in the anti-tumor protection conferred by cDC2 vaccination.

(A-B) Percentages of NK and NKT cells (A) and $IFN\gamma^{+}$ NK cells (B) in LLC-OVA tumors after vaccination with LLC-OVA-derived cDC2 following the protocol depicted in Figure 8A in the manuscript. (C) Non-vaccinated or cDC2-vaccinated mice were treated every 2-3 days as from the day before tumor inoculation with anti-NK1.1 depleting Ab. Representative plots of 13-day old LLC-OVA tumors gated on $CD45^{+}$ $CD11b^{-}$cells show the amount of NK cells. (D-E) Growth curve (D) and tumor weights (E) of LLC-OVA tumors after vaccination with LLC-OVA derived cDC2 in untreated or anti-NK1.1 treated mice. n ≥ 6. Statistical analysis by one-way ANOVA. *, $p < 0.05$; **, $p < 0.01$; ***, $p < 0.001$; ****, $p < 0.0001$.

Also the discordance they notice between the in vivo protection data, where the cDC2 subset is superior to the cDC1 subset, and the multimer staining for CTLs where cDC1 subset induced higher %, also requires a more thorough analysis, e.g. studying the effect on the innate immune rejection mechanisms such as NK cells.

Reply: Vaccination with the cDC2 subset is superior to protect against subsequent LLC-OVA tumor growth despite the superior CTL-inducing capacity of cDC1. This is not a general rule, since in the case of B16-OVA, cDC1 vaccination is more efficient.

New experiments have clearly indicated a non-redundant role for Th17 cells in the protective effect of cDC2 vaccination against LLC-OVA tumor growth. Th17 cells are only induced by tumor-associated cDC2. Moreover, LLC tumor growth is not only regulated by tumor-infiltrating lymphocytes, but also by the phenotype of tumor-associated myeloid cells such as TAM, whereby M2-oriented TAM promote tumor progression. Overall CD11b^{hi} Ly6C^{lo} Ly6G^{neg} TAM numbers only showed a trend towards a reduction in cDC2-vaccinated mice (Figure 9D in the manuscript). Importantly however, within the TAM compartment, cDC2 vaccination caused a shift towards more M1-like MHC-II^{high} TAM (i.e. a lower MHC-II^{low}/MHC-II^{high} TAM ratio) (Figures 9E and 9F in the manuscript). In addition, these MHC-II^{high} TAM had a more pronounced M1 phenotype as compared to those from non-vaccinated animals. These data show that the myeloid compartment of LLC-OVA tumors from cDC2-vaccinated mice is dominated by strongly M1-oriented TAM, which is likely a prerequisite for anti-tumor T-cell cytotoxicity to occur in this tumor model.
Of note, B16-OVA tumors are much less infiltrated by TAM, resulting in a less suppressive tumor microenvironment. This likely explains the higher efficiency of cDC1 vaccination in this model, since activated CTL can kill cancer cells without a prior need for reconversion of the TAM population.

Minor comment;
The colors and shapes of the symbols in Fig 8 B are mixed up.

Reply: We thank the reviewer for noticing this error and corrected the symbols in figure 8B and 8C according to the figure legend.

<u>Reviewers' Comments:</u>

Reviewer #1 (Remarks to the Author):

My concerns have been addressed.

Reviewer #2 (Remarks to the Author):

The authors perfectly addressed all critical questions and comments. A lot of additional experiments were performed and the manuscript was improved significantly. Importantly, the authors now have critical results in the text and all secondary and control results are now in supplementary section. This will definitely helps readers to analyse this interesting and quite loaded manuscript. There are no additional comments or suggestions.

Reviewer #3 (Remarks to the Author):

The authors clearly have vested a large amount of work on this new version of the manuscript, also adding new experiments. From this new data and from their responses to my comments, which are long and very detailed (sometimes unnecessarily detailed and long), it seems that they can in satisfactory way respond to my comments and critique. My concern that the effect of the DC vaccination is not very impressive however remains. But given the fact that these DCs are freshly isolated, and have not received any additional antigen loading, the results are significant and could be meaningful. Also, they have now added an extensive amount of work, analyzing the mechanism of protection in vivo by antibody depletion experiments. The finding that particularly CD4+ Th17 T cells are important in the cDC2 induced protection has added importance to the manuscript.

Response to all reviewers

We thank the reviewers for re-evaluating our adapted manuscript and appreciate their positive assessment.

Reviewer #1 (Remarks to the Author):

My concerns have been addressed.

Reviewer #2 (Remarks to the Author):

The authors perfectly addressed all critical questions and comments. A lot of additional experiments were performed and the manuscript was improved significantly. Importantly, the authors now have critical results in the text and all secondary and control results are now in supplementary section. This will definitely helps readers to analyse this interesting and quite loaded manuscript. There are no additional comments or suggestions.

Reviewer #3 (Remarks to the Author):

The authors clearly have vested a large amount of work on this new version of the manuscript, also adding new experiments. From this new data and from their responses to my comments, which are long and very detailed (sometimes unnecessarily detailed and long), it seems that they can in satisfactory way respond to my comments and critique. My concern that the effect of the DC vaccination is not very impressive however remains. But given the fact that these DCs are freshly isolated, and have not received any additional antigen loading, the results are significant and could be meaningful. Also, they have now added an extensive amount of work, analyzing the mechanism of protection in vivo by antibody depletion experiments. The finding that particularly CD4+ Th17 T cells are important in the cDC2 induced protection has added importance to the manuscript.
